# Supplementary material for: A model for cis-regulation of transcriptional condensates and gene expression by proximal lncRNAs
Source: Biophys J. 2023 Jun 5;122(13):2757–72. doi: 10.1016/j.bpj.2023.05.032 (PMC10397817; doi:10.1016/j.bpj.2023.05.032)
Supplement: Document S1. Figures S1–S9 and Tables S1 and S2 [file mmc1.pdf]

Biophysical Journal, Volume *122*

Supplemental information

A model for *cis*-regulation of transcriptional condensates and gene expression by proximal lncRNAs

Pradeep Natarajan, Krishna Shrinivas, and Arup K. Chakraborty

A model for *cis*-regulation of transcriptional condensates and gene expression by proximal lncRNAs

Pradeep Natarajan[1], Krishna Shrinivas[2], and Arup K. Chakraborty[1,3,4,5,6,*]

[1]Department of Chemical Engineering, Massachusetts Institute of Technology, Cambridge MA 02139, USA
[2]NSF-Simons Center, Harvard University, Cambridge MA 02139, USA
[3]Department of Physics, Massachusetts Institute of Technology, Cambridge MA 02139, USA
[4]Institute of Medical Engineering and Science, Massachusetts Institute of Technology, Cambridge MA 02139, USA
[5]Ragon Institute of Massachusetts General Hospital, Massachusetts Institute of Technology and Harvard University, Cambridge MA 02139, USA
[6]Department of Chemistry, Massachusetts Institute of Technology, Cambridge MA 02139, USA
[*]Correspondence: arupc@mit.edu

Supporting Material

CONTENTS

S1 Typical distance between lncRNA loci (RL) and binding loci (BL) 1

S2 Model description 2
S2.1 Free-energy of interactions . . . 2
S2.1.1 Protein-Protein and Protein-BL interactions . . . 3
S2.1.2 Protein-RNA interactions . . . 4
S2.1.3 lncRNA-lncRNA and lncRNA-RL interactions . . . 6
S2.1.4 Parameters associated with the free energy . . . 7
S2.2 Dynamical equations . . . 7
S2.2.1 Dynamics of condensate formation . . . 7
S2.2.2 Dynamics of active transcription . . . 8
S2.2.3 Parameters associated with dynamics . . . 9
S2.3 Formulae to calculate different quantities to analyze simulation results . . . 9

S3 Condensate dynamics and mRNA transcription in the absence of actively transcribing lncRNAs 10

S4 Supplemental figures 11

S1 TYPICAL DISTANCE BETWEEN LNCRNA LOCI (RL) AND BINDING LOCI (BL)

lncRNA-coding loci are usually within a genomic distance of 100 kb from the promoter of protein-coding genes (1). Super-enhancers are typically located at a distance of around 250 kb from the promoters (2). Therefore, lncRNA-coding loci are usually located within a genomic distance of 350 kb of promoters, enhancers, and super-enhancers which we collectively call the binding loci (BL). To translate this genomic distance into a spatial distance, we use the worm-like chain model of chromatin described by Beltran et al. (3) that takes into account how nucleosome heterogeneity and different linker DNA lengths. The typical length of linker DNA in euchromatin of human cells is around 50 bp (4), and the Kuhn length of chromatin with this linker length is 37.8 nm (3).

Using the size of a nucleotide as 0.34 nm, the number of base pairs present in this Kuhn segment = 37.8 nm/0.34 nm × (146 bp in nucleosome + 50 bp in linker DNA)/50 bp linker DNA = 436 bp.

Therefore, a 1 kb separation along the genome corresponds to ≈ 2 Kuhn segments. Similarly, 350 kb corresponds to ≈ 800 Kuhn segments. Using a random polymer model as a crude approximation, end-end distance = (# of Kuhn segments)$^{1/2}$×Kuhn length, the average spatial distance between lncRNA-coding loci and the BL comes out to be ≈ 1 μm. Super-resolution microscopy studies reveal that transcriptional condensates that form at super-enhancers have a diameter in the range of 200-600 nm (5). Thus, the spatial distance between lncRNA-coding loci and promoters/super-enhancers is of the same order as the size of transcriptional condensates.

S2 MODEL DESCRIPTION

As mentioned in the paper, the model contains two components: the free energy of interactions between the species and the dynamical equations. We take a deeper look at the free-energy expression and its associated parameters in section S2.1. We take a deeper look at the dynamical equations and their associated parameters in section S2.2

S2.1 Free-energy of interactions

The free energy of interactions between the different species is given by the below expression:

$$F[\phi_P, \phi_R, \phi_M, \vec{r}] = F_{FH}[\phi_P, \phi_R, \phi_M] + F_{BL}[\phi_P, \vec{r}] + F_{RL}[\phi_R, \vec{r}] + F_{surf}[\phi_P] \tag{1}$$

- Here, $F_{FH}[\phi_P, \phi_R, \phi_M]$ is a Flory-Huggins free energy that captures the self and cross interactions between transcriptional proteins, lncRNA, and the mRNA.

$$F_{FH}[\phi_P, \phi_R, \phi_M] = \underbrace{\frac{\phi_P}{N_P}\log\phi_P + \frac{\phi_R}{N_R}\log\phi_R + \frac{\phi_M}{N_M}\log\phi_M + (1-\phi_P-\phi_R-\phi_M)\log(1-\phi_P-\phi_R-\phi_M)}_{\text{Entropy}}$$
$$- \underbrace{\chi_P\phi_P^2}_{\text{Protein-Protein}} - \underbrace{\chi_{PR}\phi_P(\phi_R+\phi_M)}_{\text{Protein-RNA}} + \underbrace{\chi_R(\phi_R+\phi_M)^2}_{\text{RNA-RNA}} \tag{2}$$

Here, ϕ_P, ϕ_R, and ϕ_M are the volume fractions of protein, lncRNA, and mRNA species in the solution. N_P, N_R, and N_M are the lengths of the protein, lncRNA, and mRNA species in units of solvent volume. The parameters χ_P, χ_R, and χ_{PR} correspond to the Flory-Huggins χ parameters that capture the mean-field pairwise interaction strength between protein-protein, RNA-RNA, and protein-RNA species respectively. We assume that the lncRNA and mRNA species interact with the same strength χ_{PR} with the proteins and χ_R with each other, as they have similar lengths and charges that are not too different (6). Since the volume fraction of the proteins can be converted to protein concentrations by a multiplicative scaling factor, we will be using volume fractions and concentrations as semantically equivalent while keeping this distinction in mind.

- The term $F_{BL}[\phi_P, \vec{r}]$ captures the interaction free energy of transcriptional proteins with the BL.

$$F_{BL}[\phi_P, \vec{r}] = -c_P e^{-\frac{|\vec{r}-\vec{r}_{BL}|^2}{\sigma_{BL}^2}}\phi_P \tag{3}$$

Here, c_P is a strength of interaction between the BL and transcriptional proteins, σ_{BL} represents the spatial extent of these interactions, $\vec{r}_{BL}$ is the position of the BL in space, and $\vec{r}$ is the position vector.

- The term $F_{RL}[\phi_R, \vec{r}]$ captures the interaction free energy of lncRNAs with the RL.

$$F_{RL}[\phi_R, \vec{r}] = -c_R e^{-\frac{|\vec{r}-\vec{r}_{RL}|^2}{\sigma_{RL}^2}}\phi_R \tag{4}$$

Here, c_R is a strength of interaction between the RL and lncRNAs, σ_{RL} represents the spatial extent of these interactions, and $\vec{r}_{RL}$ is the position of the RL in space.

- Finally, the term $F_{surf}[\phi_P] = \frac{\kappa}{2}|\phi_P|^2$ is a surface tension term that penalizes sharp gradients in protein concentration, with κ being the strength of this energy penalty.

We take a deeper look at the cooperative effects that can arise when these interactions are combined together in the subsequent sections. Specifically, section S2.1.1 looks at the cooperative effects of protein-protein and protein-BL interactions and maps out parameter regimes where the protein phase separates. The choice of parameters χ_P and c_P used in this study shows qualitatively similar behavior to experiments (Figure S2). The section S2.1.3 describes how the free energy $F_{RL}[\phi_R, \vec{r}]$ leads to the localization of lncRNA near the RL. Section S2.1.2 describes the RNA-Protein phase diagram that arises due to the Flory-Huggins interaction free energy between these species. The parameters χ_R and χ_{PR} used in this study qualitatively match the re-entrant phase diagram observed in experiments S5.

S2.1.1 Protein-Protein and Protein-BL interactions

Interactions between intrinsically disordered regions of transcriptional proteins can promote phase separation (7, 8). These protein-protein interactions that favor phase separation can be qualitatively captured using a mean-field free energy expression from the Flory-Huggins theory of polymer solutions:

$$F_{FH}[\phi_P] = \frac{\phi_P}{N_P}\log\phi_P + (1-\phi_P)\log(1-\phi_P) - \chi_P\phi_P^2 \tag{5}$$

Here, ϕ_P is the volume fraction of protein in the solution, N_P is the length of the protein in units of solvent volume, and χ_P is the Flory-Huggins interaction parameter that captures the magnitude of attractive interactions between protein molecules. Since the volume fraction of the proteins can be converted to protein concentrations by a multiplicative scaling factor, we will be using volume fractions and concentrations as semantically equivalent while keeping this distinction in mind.

For this study, we coarse-grained the proteins as having $N_P = 5$ beads. To study the phase separation of this protein, the interaction strength χ_P has to be large enough to support phase separation into two phases for some range of concentrations. According to the Flory-Huggins theory, for a polymer having N beads, $\chi > 0.5 + 1/\sqrt{N}$ results in phase separation into a dense and light phase. We chose a value of $\chi_P = 1.1$ (which is $> 0.5 + 1/\sqrt{5}$) for this study. The corresponding plot of chemical potential with the spinodal and binodal boundaries marked are depicted in figure S1A. Other than this, the particular numerical values of χ_P and N_P do not really affect the qualitative phase diagram. They only affect the concentration thresholds of the binodal and spinodal boundaries.

In addition to protein-protein interactions, there is also a surface tension associated with the protein molecules, and the protein molecules are attracted to BL through the free energy $F_{BL}[\phi_P, \vec{r}]$. The combined effect of surface tension, protein-protein, and protein-BL interactions is captured by the below free energy expression:

$$F_P[\phi_P, \vec{r}] = F_{FH}[\phi_P] + F_{BL}[\phi_P, \vec{r}] + F_{surf}[\phi_P] = \\ \phi_P\log\phi_P + (1-\phi_P)\log(1-\phi_P) - \chi_P\phi_P^2 - c_P e^{-|\vec{r}|^2/\sigma^2}\phi_P + \frac{\kappa}{2}|\nabla\phi_P|^2 \tag{6}$$

In the above, we have set $\vec{r}_{BL} = 0$ meaning that the BL is at the origin. We set the value of the surface tension parameter to be small ($\kappa << 1$), enough to ensure that the condensate stays spherical while F_{FH} and F_{BL} dominate the free energy expression and dictate the phase diagrams. The chemical potential associated with this free energy is:

$$\mu_P[\phi_P, \vec{r}] = \frac{\delta \int F_P dV}{\delta\phi_P} = \frac{1+\log\phi_P}{N_P} - (1+\log(1-\phi_P)) - 2\chi_P\phi_p - c_P e^{-|\vec{r}|^2/\sigma^2} \tag{7}$$

Figure S1A plots this chemical potential as a function of ϕ_P for different values of distance r from the center of the region in space containing DNA binding sites. When the average protein concentration in the system ϕ_P is within the spinodal boundary, the free energy F_P is concave function of ϕ_P with $\frac{\partial^2 F_P}{\partial\phi_P^2} = \frac{\partial\mu_P}{\phi_P} < 0$. The system is unstable and its free energy can be minimized by splitting into a dense phase rich in protein and a light phase depleted in protein with their respective compositions ϕ_P given by the binodal boundary.

We obtained the equilibrium profiles $\phi_P(r)$ as a function of the radial position r in a circular domain by starting with a spatially uniform protein concentration and simulating the relaxation using Model B dynamics till steady-state (9):

$$\frac{\partial\phi_P(\vec{r},t)}{\partial t} = \vec{\nabla}.\left(D_P\phi_P\left(\vec{\nabla}\mu_P\right)\right) \tag{8}$$

The steady-state solution of the above equations is also the solution to the equation $\mu_p[\phi_P^{eq}, \vec{r}] =$ constant, which is the criterion for chemical equilibrium. The constant is specified if we fix the average protein concentration in the system to a fixed value i.e. $1/V\int\phi_P^{eq}(\vec{r})dV = \phi_P^{avg}$. Expanding out the equation:

$$\mu_P[\phi_P^{eq}, \vec{r}] = \frac{1+\log\phi_P^{eq}}{N_P} - (1+\log(1-\phi_P^{eq})) - 2\chi_P\phi_P^{eq} - c_P e^{-|\vec{r}|^2/\sigma^2} = \text{constant} \tag{9}$$

S1B and S1C depict the profiles $\phi_p(r)$ as a function of the radial position r for different values of ϕ_P^{avg} and c_P. Consider the system having average protein concentration ϕ_P^{avg} well outside the binodal region. The spatially varying free energy benefit $F_{BL}[\phi_P, \vec{r}]$ conferred by the protein-BL interactions leads to an accumulation of protein at the BL. Beyond a threshold value of ϕ_P^{avg} or c_P, the local protein concentration at the BL $\phi_P^{eq}(r=0)$ can cross the spinodal boundary and start forming a dense

phase of protein at $r = 0$. In figure S1C, we observe that a small increase in c_P from 0.05 to 0.1 results in a large increase in $\phi_P^{eq}(r=0)$. In the same way, for every value of ϕ_P^{avg}, sufficiently strong protein-DNA interactions with $c_P > c_P^*(\phi_P^{avg})$ can result in the formation of a dense phase of protein at much lower protein concentrations (ϕ_P^{avg}) well below the spinodal boundary. $c_P^*(\phi_P^{avg})$ is defined graphically in figure S1A.

Figure S2A plots contours of the condensate area as we vary ϕ_P^{avg} and c_P in the simulations. We define a transcriptional condensate as a region in space having a protein concentration $\phi_P > 0.3$, which represents the center of the spinodal region. When we have a sufficient amount of protein ϕ_P^{avg} in the system or sufficiently strong magnitude c_P of the protein-DNA interactions, a condensate of non-zero area is nucleated. The white dotted line represents the curve $c_P = c_P^*(\phi_P^{avg})$. The simulations agree with our theoretical prediction that a condensate nucleates when $c_P > c_P^*(\phi_P^{avg})$. We note that the phase diagram in figure S2B is qualitatively similar to the experimentally measured phase diagrams S2B reported in (7).

We chose the protein concentration in the system $\phi_P^{avg} = 0.04$, which is well below the binodal boundary shown in figure S1A. This was chosen to be consistent with the biological observation that *in vivo* transcriptional protein concentrations are much lower than the binodal concentrations (7). Simulations for results in the main manuscript are done with a c_P value close to the critical boundary $c_P = c_P^*(\phi_P^{avg})$, to illustrate how the presence of other RNA species can alter the condensate formation process. Therefore, simulation results in the main manuscript are done for the parameters $\phi_P^{avg} = 0.04, c_p = c_P^*(\phi_P^{avg} = 0.04) \approx 0.2$ unless stated otherwise.

S2.1.2 Protein-RNA interactions

The disordered regions of many transcriptional proteins contain a net positive charge. They can attract negatively charged RNAs via screened electrostatic interactions (10). Prior studies have shown that the qualitative features of phase diagrams of charged polymers in solutions interacting via screened electrostatic interactions can be qualitatively captured via a mean-field Flory Huggins free energy expression (11). For a Protein-RNA solution, the free energy expression can be written down to be:

$$F_{FH}[\phi_P, \phi_R] = \frac{\phi_P}{N_P}\log\phi_P + \frac{\phi_R}{N_R}\log\phi_R + (1-\phi_P-\phi_R)\log(1-\phi_P-\phi_R) - \chi_P\phi_P^2 - \chi_{PR}\phi_P\phi_R + \chi_R\phi_R^2 \tag{10}$$

Here, χ_P captures the magnitude of protein-protein attractive interactions, χ_{PR} captures the RNA-protein attractive interactions and χ_R captures the magnitude of the screened RNA-RNA electrostatic repulsion. N_R is the coarse-grained length of the RNA species. The typical length of disordered regions of transcriptional proteins is not more than 1000 amino acids (10). On the other hand, lncRNAs and mRNAs have a length of the order of magnitude $\sim$ 10000 base pairs, around 10 times longer than proteins(6). Therefore, we set the length of the RNA polymers as $N_R = N_M = 50$ beads, 10 times the size of the protein polymer $N_P = 5$.

The solvent entropy term in equation 10 i.e. $(1-\phi_P-\phi_R)\log(1-\phi_P-\phi_R)$ can be rearranged as:

$$\begin{aligned}(1-\phi_P-\phi_R)\log(1-\phi_P-\phi_R) &= (1-\phi_P-\phi_R)\left[\log(1-\phi_P) + \log\left(1-\frac{\phi_R}{1-\phi_P}\right)\right] \\ &= (1-\phi_P)\log(1-\phi_P) + (1-\phi_P)\log\left(1-\frac{\phi_R}{1-\phi_P}\right) - \phi_R\log(1-\phi_P) - \phi_R\log\left(1-\frac{\phi_R}{1-\phi_P}\right)\end{aligned} \tag{11}$$

At dilute RNA and Protein concentrations $\phi_P << 1$ and $\phi_R << 1$, using the expansions $\log(1-x) = -x - x^2/2 + ...$ and $1/(1-x) = 1 + x + x^2 + ...$, the above terms can be expanded to yield the following expression for solvent entropy:

$$(1-\phi_P-\phi_R)\log(1-\phi_P-\phi_R) = (1-\phi_P)\log(1-\phi_P) + \phi_R\phi_P + \frac{\phi_R^2}{2} + \frac{\phi_R^2\phi_P}{2} + \frac{\phi_R\phi_P^2}{2} + \frac{\phi_R^2\phi_P^2}{2} + ... \tag{12}$$

Under this approximation, the free energy in equation 10 gets modified as:

$$\begin{aligned}F_{FH,mod}[\phi_P, \phi_R] = {}& \frac{\phi_P}{N_P}\log\phi_P + (1-\phi_P)\log(1-\phi_P) - \chi_P\phi_P^2 \\ &+ \frac{\phi_R}{N_R}\log\phi_R + (1-\chi_{PR})\phi_P\phi_R + \left(\chi_R + \frac{1}{2}\right)\phi_R^2 + \frac{\phi_R^2\phi_P + \phi_R\phi_P^2 + \phi_R^2\phi_P^2}{2}\end{aligned} \tag{13}$$

The above free energy expression has nicer numerical properties and leads to lower numerical errors compared to the free energy expression 10 when simulating the dynamical equations in sections S2.2.1 and S2.2.2. Therefore, we will use this free energy expression for the rest of the study.

Before jumping to this, let us first establish that both F_{FH} and $F_{FH,mod}$ do not lead to any qualitative difference in the equilibrium RNA-protein phase diagram due to the approximations introduced. From the free energy expressions 10 and 13, we can generate the phase diagram by analyzing the Jacobian matrix of $F_{FH}[\phi_P, \phi_R]$ and $F_{FH,mod}[\phi_P, \phi_R]$ and with respect to the variables ϕ_P and ϕ_R:

$$J_{FH} = \begin{bmatrix} \frac{\partial^2 F_{FH}}{\partial \phi_P^2} & \frac{\partial^2 F_{FH}}{\partial \phi_P \partial \phi_R} \\ \frac{\partial^2 F_{FH}}{\partial \phi_P \partial \phi_R} & \frac{\partial^2 F_{FH}}{\partial \phi_R^2} \end{bmatrix} = \begin{bmatrix} \frac{1}{N_P \phi_P} + \frac{1}{1-\phi_P-\phi_R} - 2\chi_P & \frac{1}{1-\phi_P-\phi_R} - \chi_{PR} \\ \frac{1}{1-\phi_P-\phi_R} - \chi_{PR} & \frac{1}{N_R \phi_R} + \frac{1}{1-\phi_P-\phi_R} + 2\chi_R \end{bmatrix} \tag{14}$$

$$J_{FH,mod} = \begin{bmatrix} \frac{\partial^2 F_{FH,mod}}{\partial \phi_P^2} & \frac{\partial^2 F_{FH,mod}}{\partial \phi_P \partial \phi_R} \\ \frac{\partial^2 F_{FH,mod}}{\partial \phi_P \partial \phi_R} & \frac{\partial^2 F_{FH,mod}}{\partial \phi_R^2} \end{bmatrix} = \begin{bmatrix} \frac{1}{N_P \phi_P} + \frac{1}{1-\phi_P} - 2\chi_P + \phi_R + \phi_R^2 & 1 - \chi_{PR} + \phi_R + \phi_R + 2\phi_P\phi_R \\ 1 - \chi_{PR} + \phi_R + \phi_R + 2\phi_P\phi_R & \frac{1}{N_R \phi_R} + (2\chi_R + 1) + \phi_P + \phi_P^2 \end{bmatrix} \tag{15}$$

The system is unstable and can undergo phase separation in the regions in the $\phi_P - \phi_R$ space where the Jacobian has at least one negative eigenvalue, which corresponds to a region of concavity of the free energy. The brown regions in figure S4A and S4B correspond to this region of spinodal instability. We can see from these figures that the shapes of the spinodal regions are qualitatively similar for both the free energy expression F_{FH} and $F_{FH,mod}$.

When ϕ_P and ϕ_R are within this region, the system splits into two phases: a dense phase rich in RNA and protein and a light phase poor in both RNA and protein. The coexistence compositions $(\phi_P^{dense}, \phi_R^{dense})$ and $(\phi_P^{light}, \phi_R^{light})$ are obtained by solving the equations for equality of chemical potentials and osmotic pressures in the two phases, which form the criteria for multiphase equilibrium:

$$\mu_P(\phi_P^{dense}, \phi_R^{dense}) = \mu_P(\phi_P^{light}, \phi_R^{light}) \tag{16}$$

$$\mu_R(\phi_P^{dense}, \phi_R^{dense}) = \mu_R(\phi_P^{light}, \phi_R^{light}) \tag{17}$$

$$\Pi(\phi_P^{dense}, \phi_R^{dense}) = \Pi(\phi_P^{light}, \phi_R^{light}) \tag{18}$$

where the chemical potentials of protein and RNA are respectively $\mu_P = \frac{\partial F_{FH}}{\partial \phi_P}$, $\mu_R = \frac{\partial F_{FH}}{\partial \phi_R}$, and the osmotic pressure $\Pi = k_B T / V_{solution} \Big(F_{FH} - \mu_P \phi_P - \mu_R \phi_R \Big)$. In addition, the total amount of protein (ϕ_P) and RNA (ϕ_R) constrain the dense and light phase compositions in the following way:

$$\nu \phi_P^{dense} + (1 - \nu)\phi_P^{light} = \phi_P \tag{19}$$

$$\nu \phi_R^{dense} + (1 - \nu)\phi_R^{light} = \phi_R \tag{20}$$

The system of 5 equations 16- 20, need to be solved to get the variables ϕ_P^{dense}, ϕ_P^{light}, ϕ_R^{dense}, ϕ_R^{light}, and ν. Here, ν is the volume fraction of the dense phase.

We can obtain the full phase diagram upon varying ϕ_P and ϕ_R for the two different free energy expressions 10 and 13 using the procedure described above, which are shown in figures S4A and S4B respectively. The dotted lines in figures S4A and S4B show the tie-lines connecting coexistence concentrations between the dense and light phases of protein. The brown regions correspond to the regions of spinodal instability. We can see that the shapes of the spinodal boundaries and the tie-lines are qualitatively the same for both F_{FH} and $F_{FH,mod}$. Therefore, we will use $F_{FH,mod}$ for the rest of the study.

Experimentally measured phase diagrams of RNA with transcriptional proteins exhibits a re-entrant behavior, where the protein partitioning to the dense phase initially increases and then decreases upon increasing the RNA concentration as shown in figure S5B (10). To qualitatively capture this phenomenon, we require χ_{PR} to be attractive so that there is an increase in protein partitioning to the dense phase in the presence of RNA. A value of $\chi_{PR} = 1.2$ worked well for our model. We also require the RNA-RNA repulsion strength to be stronger than the RNA-protein attraction to result in the dissolution of the dense phase of protein at high RNA concentrations. We chose a value of $\chi_R = 2.0 > \chi_{PR} = 1.2$. For this choice of parameters i.e. $\chi_{PR} = 1.2, \chi_R = 2.0$ used in this study, the partition ratio $(\phi_P^{dense}/\phi_P^{light})$ calculated by solving equations 16-20 and varying RNA concentration ϕ_R (figure S5A) qualitatively matches very well with experimentally measured re-entrant phase diagrams for transcriptional coactivators with RNA species as shown in S5B.

We can also gain more insight from the full phase diagram in S4B. As we increase the RNA concentration ϕ_R in the system for a constant $\phi_P = 0.3$, we observe that the horizontal width of the coexistence curve initially increases and then decreases S4B. RNA promotes the partitioning of the protein into the dense phase at low ϕ_R by virtue of its attractive interactions with the protein. At high ϕ_R, the RNA-RNA repulsive interactions and the entropic penalty of excluding the solvent from the dense phase results make the partitioning of protein into a dense phase unfavorable. When the RNA concentrations are high, phase separation is completely suppressed. We can see that beyond a critical value of the RNA concentration i.e. $\phi_R > \phi_R^c = 0.17$, there is no phase separation and formation of a two-phase region (figure S4B).

S2.1.3 lncRNA-lncRNA and lncRNA-RL interactions

Recent studies show that long-non coding RNAs (lncRNAs) tend to be present in locally high concentrations near their coding loci (12), which we term in this study as the RL. Although the mechanisms that cause this localization is poorly understood, it could be a consequence of equilibrium effects such as tethering of lncRNAs to their DNA loci by proteins such as Polymerase II (13) and YY1 (14), or non-equilibrium effects such as localized production of lncRNAs coupled with diffusion (15).

In this section, we will model equilibrium mechanisms that keep lncRNAs bound to their DNA coding loci using similar arguments as section S2.1.1. The free energy of binding of lncRNAs to their RL as described before is:

$$F_{RL}(\phi_R, \vec{r}) = -c_R e^{-|\vec{r}|^2/\sigma^2} \phi_R \tag{21}$$

When we combine this with a Flory-Huggins expression for the lncRNA species that takes into account lncRNA-lncRNA repulsion and entropy of the lncRNA polymer and solvent, we get the below expression for the total free energy of the lncRNA-RL system:

$$F_R[\phi_R, \vec{r}] = \frac{\phi_R}{N_R} \log \phi_R + (1-\phi_R)\log(1-\phi_R) + \chi_R \phi_R^2 - c_R e^{-|\vec{r}|^2/\sigma^2} \phi_R \tag{22}$$

Here, N_R is the length of the RNA polymer, and χ_R is the magnitude of the repulsive strength between the RNA species, whose values are set to $N_R = 50$ and $\chi_R = 2.0$ as justified in the section S2.1.2.

Transcription factors bind to the DNA with a binding affinity in the 1-100 nM range (16) while the binding affinity of lncRNAs with chromatin binding proteins is slightly weaker, around 100-1000 nM (17). Assuming a similar density of binding sites for lncRNA and tethering proteins on the DNA, we expect c_R to be typically much lower than c_P. The strength of the lncRNA-RL interactions was set to a similar value as the protein i.e. $c_R = c_P = 0.2$ for this study, with the understanding that this probably represents an upper limit on the strength of the lncRNA-RL interactions.

The chemical potential associated with this free energy in equation 22:

$$\mu_R[\phi_R, \vec{r}] = \frac{\delta \int F_R dV}{\delta \phi_R} = \frac{1 + \log \phi_R}{N_R} - (1 + \log(1-\phi_R)) + 2\chi_R \phi_R - c_R e^{-|\vec{r}|^2/\sigma^2} \tag{23}$$

We obtained the equilibrium profiles $\phi_R(r)$ in a circular domain by starting with a spatially uniform RNA concentration ϕ_R^{avg} and simulating the Model B dynamics (9) until steady-state:

$$\frac{\partial \phi_R(\vec{r})}{\partial t} = \vec{\nabla}.\left(D_R \phi_R \left(\vec{\nabla}\mu_R\right)\right) \tag{24}$$

The steady-state solution of the above equations is also the solution to the equation $\mu_R[\phi_R^{eq}, \vec{r}] =$ constant, which is the criterion for chemical equilibrium. The constant is chosen in such a way that $1/V \int \phi_R^{eq}(\vec{r}) dV = \phi_R^{avg}$. Expanding out the equation:

$$\mu_R[\phi_R^{eq}, \vec{r}] = \frac{1 + \log \phi_R^{eq}}{N_R} - (1 + \log(1-\phi_R^{eq})) + 2\chi_R \phi_R^{eq} - c_R e^{-|\vec{r}|^2/\sigma^2} = \text{constant} \tag{25}$$

Figure S3A plots this chemical potential as a function of ϕ_R for different values of distance r from the center of the RL. Figure S3B depicts the equilibrium profiles $\phi_R(r)$ in for different values of ϕ_R^{avg}.

For low values of ϕ_R, the dominant ϕ_R-dependent term in the chemical potential is $\frac{\log \phi_R}{N_R}$, which comes from the entropy of RNA in solution. The equilibrium concentration profile is shaped by the balance between lncRNA-RL interactions that attract the lncRNA to the RL and thermal fluctuations that tend to equalize concentrations everywhere. In this regime, the equilibrium profile $\phi_R^{eq}(r)$ is obtained by solving the equation:

$$\mu_R[\phi_R^{eq}, \vec{r}] \approx \frac{1 + \log \phi_R^{eq}}{N_R} - c_R e^{-|\vec{r}|^2/\sigma^2} = \text{constant} \tag{26}$$

For intermediate values of ϕ_R, the dominant ϕ_R-dependent term in the chemical potential is $2\chi_R\phi_R$, which comes from the lncRNA-lncRNA repulsive interactions. The equilibrium concentration profile is shaped by the balance between lncRNA-RL interactions that attract the lncRNA to the RL and RNA-RNA repulsions that tend to equalize concentrations everywhere. In this regime, the equilibrium profile $\phi_R^{eq}(r)$ is obtained by solving the equation:

$$\mu_R[\phi_R^{eq}, \vec{r}] \approx 2\chi_R\phi_R^{eq} - c_R e^{-|\vec{r}|^2/\sigma^2} = \text{constant} \tag{27}$$

In this regime, the free energy penalty imposed by lncRNA-lncRNA repulsion linearly scales with ϕ_R. Therefore, the RL gets saturated with a fixed amount of lncRNA and any additional lncRNA added will get uniformly distributed across the system. This is the reason that the profiles $\phi_R^{eq}(r)$ in figure S3B for intermediate values of $\phi_R^{avg} = 0.005$ and $\phi_R^{avg} = 0.01$ maintain their Gaussian shape while just being shifted up by some constant amount.

S2.1.4 Parameters associated with the free energy

Parameter	Value	Description	Rationalization
N_P	5.0	Length of coarse-grained protein sequence	Section S2.1.1
χ_P	1.1	Protein-protein attraction strength	Section S2.1.1
c_P	0.2	Protein-BL interaction strength	Section S2.1.1
ϕ_P^c	0.3	Protein concentration threshold to determine dense phase/condensate	Figure S4B
N_R	50.0	Length of coarse-grained lncRNA sequence	Section S2.1.3
N_M	50.0	Length of coarse-grained mRNA sequence	Section S2.1.3
χ_R	2.0	RNA-RNA repulsion strength	Section S2.1.2
χ_{PR}	1.2	RNA-protein interaction strength	Section S2.1.2
c_R	0.2	lncRNA-RL interaction strength	Section S2.1.3

Table S1: Table of parameters associated with the free energy expression

S2.2 Dynamical equations

In this section, we will look at how the dynamical equations for the concentration fields of the RNA and protein species are written for the two different cases studied in the paper: (i) condensate formation and (ii) active transcription

S2.2.1 Dynamics of condensate formation

The dynamics of transcriptional condensates happen over time scales of minutes (18). At these time scales, RNAs are not being turned over (19) and the total amount of RNAs in the system can be considered a conserved parameter. The same applies to proteins which are also quite stable over time scales of minutes. Taking into account all the relevant interactions between the proteins, lncRNAs, BL, and RL as described in section S2.1 the overall free energy of this system is:

$$\begin{aligned} F[\phi_P, \phi_R] = & \underbrace{\frac{\phi_P}{N_P}\log\phi_P + \frac{\phi_R}{N_R}\log\phi_R + (1-\phi_P-\phi_R)\log(1-\phi_P-\phi_R)}_{\text{Entropy}} - \underbrace{\chi_P\phi_P^2}_{\text{Protein-Protein}} - \underbrace{\chi_{PR}\phi_P\phi_R}_{\text{Protein-RNA}} + \underbrace{\chi_R\phi_R^2}_{\text{RNA-RNA}} \\ & - \underbrace{c_P e^{-|\vec{r}-\vec{r}_{BL}|^2/\sigma^2}\phi_P}_{\text{Protein-chromatin}} - \underbrace{c_R e^{-|\vec{r}-\vec{r}_{RL}|^2/\sigma^2}\phi_R}_{\text{RNA-DNA}} + \underbrace{\frac{\kappa}{2}|\nabla\phi_P|^2}_{\text{Surface Tension}} \end{aligned} \tag{28}$$

Using the approximation for the solvent entropy (equation 12), the free energy can be rewritten as:

$$F[\phi_P, \phi_R] = \frac{\phi_P}{N_P}\log\phi_P + (1-\phi_P)\log(1-\phi_P) - \chi_P\phi_P^2 + \frac{\phi_R}{N_R}\log\phi_R + (1-\chi_{PR})\phi_P\phi_R + \left(\chi_R + \frac{1}{2}\right)\phi_R^2 + \frac{\phi_R^2\phi_P + \phi_R\phi_P^2 + \phi_R^2\phi_P^2}{2} - c_P e^{-|\vec{r}-\vec{r}_{BL}|^2/\sigma^2}\phi_P - c_R e^{-|\vec{r}-\vec{r}_{RL}|^2/\sigma^2}\phi_R + \frac{\kappa}{2}|\nabla\phi_P|^2 \quad (29)$$

Initially, the lncRNA species are localized at the RL with a concentration profile $\phi_R(\vec{r}, t=0) = \phi_R^{eq}(\vec{r})$, where $\phi_R^{eq}(\vec{r})$ is described in section S2.1.3. The protein concentrations are uniform throughout the domain i.e. $\phi_P(\vec{r}, t=0) = \phi_P^{avg} = constant$. The concentration profiles of the proteins and lncRNA relax to a new equilibrium. The coupled dynamics of relaxation to equilibrium can be captured by simulating the following Model B equations (9) until steady-state:

$$\frac{\partial\phi_P(\vec{r})}{\partial t} = \vec{\nabla}.\left(D_P\phi_P\left(\vec{\nabla}\mu_P\right)\right) \quad (30)$$

$$\frac{\partial\phi_R(\vec{r})}{\partial t} = \vec{\nabla}.\left(D_R\phi_R\left(\vec{\nabla}\mu_R\right)\right) \quad (31)$$

Where $\mu_P = \frac{\delta\int FdV}{\delta\phi_P}$ and $\mu_R = \frac{\delta\int FdV}{\delta\phi_R}$. The steady state of these equations is the new equilibrium profiles of the protein and lncRNA species, which can also be obtained by solving $\mu_P[\phi_P^{eq}, \phi_R^{eq}, \vec{r}]$ = constant and $\mu_R[\phi_P^{eq}, \phi_R^{eq}, \vec{r}]$ = constant. The data in figures 2 and 3 of the main text are generated using these equilibrium protein and lncRNA concentration profiles, except for the plots of the dynamics.

S2.2.2 Dynamics of active transcription

Active transcription and depletion of RNAs can change the RNA concentrations and provide a driving force that pushes the system out of equilibrium. In our model, we make a distinction between two kinds of RNAs - (i) mRNAs which are transcribed from BLs such as promoters of protein-coding genes. The production rates of mRNAs are coupled to the local protein concentrations ϕ_P and (ii) lncRNAS, which are transcribed from nearby DNA present in the vicinity of BL. The production rates of these RNAs in general do not depend on the concentration of transcriptional proteins and their transcription rate is independent of ϕ_P. This generality can be broken if there is reason to believe that the same transcriptional proteins regulate the transcription of both the mRNAs and lncRNA species for specific systems.

To first understand the effect of localized mRNA transcription on the dynamics of transcriptional condensates, we model the dynamics of $\phi_P(\vec{r}, t)$ using Model B dynamics (9) and couple this to a reaction-diffusion model for the dynamics of the concentration field that corresponds to mRNA (ϕ_M):

$$\frac{\partial\phi_P(\vec{r}, t)}{\partial t} = \vec{\nabla}.\left(D_P\phi_P\left(\vec{\nabla}\mu_P\right)\right) \quad (32)$$

$$\frac{\partial\phi_M(\vec{r}, t)}{\partial t} = D_M\nabla^2\phi_M + \underbrace{k_M e^{\frac{-|\vec{r}-\vec{r}_{BL}|^2}{\sigma^2}}}_{k_M(\vec{r})}\phi_P - k_d\phi_M \quad (33)$$

The mRNA transcription rate constant $k_M(\vec{r}) = k_M e^{\frac{-|\vec{r}-\vec{r}_{BL}|^2}{\sigma^2}}$ is assumed to be a Gaussian function, peaked around the BL, motivated by the fact that mRNA transcription starts at the promoter of the protein-coding gene and decays as we progressively move away in space. A Gaussian function models this phenomenon well and captures the relevant biology (15).

From the above equations: (i) the dynamics of the field $\phi_P(\vec{r}, t)$ is coupled to the dynamics of the field $\phi_M(\vec{r}, t)$ via the protein-RNA interactions captured by the free energy F (ii) the dynamics of the field $\phi_M(\vec{r}, t)$ is coupled to the dynamics of the field $\phi_P(\vec{r}, t)$ as the rate of production of the RNA depends on $\phi_P(\vec{r}, t)$. These couplings result in RNA production acting as feedback on the protein transport, resulting in different non-equilibrium steady states depending on the system parameters. We investigate the consequences of these equations in detail in section S3.

Next, we would like to understand how the transcriptional dynamics of lncRNAs produced near transcriptional condensates interferes and affects the dynamics of $\phi_P(\vec{r}, t)$. To study this, we compare the dynamics described by equations 32- 33 with the

below equations in the presence of the second RNA species i.e. lncRNAs, by progressively increasing the lncRNA transcription rate k_M:

$$\frac{\partial \phi_P(\vec{r},t)}{\partial t} = \vec{\nabla}.\left(D_P \phi_P \left(\vec{\nabla}\frac{\delta F}{\delta \phi_P}\right)\right) \tag{34}$$

$$\frac{\partial \phi_M(\vec{r},t)}{\partial t} = D_M \nabla^2 \phi_M + \underbrace{k_M e^{\frac{-|\vec{r}-\vec{r}_{BL}|^2}{\sigma^2}}}_{k_M(\vec{r})} \phi_P - k_d \phi_M \tag{35}$$

$$\frac{\partial \phi_R(\vec{r},t)}{\partial t} = D_R \nabla^2 \phi_R + \underbrace{k_R e^{\frac{-|\vec{r}-\vec{r}_{RL}|^2}{\sigma^2}}}_{k_R(\vec{r})} - k_d \phi_R \tag{36}$$

Here, the rate constants $k_M(\vec{r})$ and $k_R(\vec{r})$ are modeled as spatially dependent Gaussians centered around the BL and the RL. The peak values of these Gaussians are k_M and k_R and their widths are both fixed to be the same value σ for simplicity.

In the above model, the transcription of the proximal lncRNAs can perturb the dynamics of the base system described by equations 32- 33. The extent of this perturbation will depend on the parameter k_R.

S2.2.3 Parameters associated with dynamics

In the model equations D_P, D_R and D_M are the diffusivities of the transcriptional proteins, lncRNAs, and mRNAs respectively in a dilute solution. For lncRNAs and mRNAs that are being actively transcribed, we assume that they are strongly tethered to the chromatin by RNA Polymerase II, and their diffusivity is the same as the diffusivity of the BL or the RL (10). Diffusivity of actively transcribed chromatin loci are of the order $10^{-3} - 10^{-2} \mu m^2/s$ (20) which is about 10-1000 times smaller than the diffusivity of transcriptional proteins which are of the order of $0.1 - 1 \mu m^2/s$ (21). Therefore, we set $D_P = 100$ and $D_R = D_M = 0.1$ for our simulations.

k_{dR} and k_{dM} are the first-order degradation rates of the lncRNA and the protein respectively. The half-lives of RNAs span a range of time scales from minutes to hours. However, the median half-lives are not that different and are of the same order of magnitude for both mRNAs and lncRNAs – both being a few hours (19). Therefore, we set both degradation rate constants to the same value i.e. $k_{dM} = k_{dR}$. We chose a value of $k_{dM} = k_{dR} = k_d = 0.02$ for our simulations. This value was chosen such that the half-life of the RNA species $\ln 2/k_d \approx 35$ is an order of magnitude larger than the protein diffusion time scale $\tau_D = r^2/D_P = 2.25$. This is consistent with biological reality where transcriptional proteins diffuse much faster (21) than RNA half-lives (19).

The parameters k_M and k_R quantify the magnitude of mRNA and lncRNA transcription rates and σ_M and σ_R refer to the spatial extent of these molecules. Since the mean lengths of lncRNAs and mRNAs in the human genome are of the same order of magnitude (22) – around 10 kb, we set $\sigma_M = \sigma_R = \sigma = 5$.

Parameter	Value	Description
D_P	100	Protein diffusivity
D_R	0.1	lncRNA diffusivity
D_M	0.1	mRNA diffusivity
σ_R	5	Spatial extent of lncRNA locus
σ_M	5	Spatial extent of mRNA locus
k_{dR}	0.02	lncRNA degradation rate
k_{dM}	0.02	mRNA degradation rate
‘		

Table S2: Table of parameters associated with dynamical equations

S2.3 Formulae to calculate different quantities to analyze simulation results

In the below expressions, the species s could refer to transcriptional proteins, lncRNA, or mRNA.

- Concentration of a species s at the BL = $\phi_s^{BL} = \frac{\int_{|\vec{r}-\vec{r}_{BL}|<\sigma} \phi_s(\vec{r},t) dV}{\int_{|\vec{r}-\vec{r}_{BL}|<\sigma} dV}$

- Concentration of a species s outside the BL = $\phi_s^{out} = \frac{\int_{|\vec{r}-\vec{r}_{BL}|>\sigma} \phi_s(\vec{r},t)dV}{\int_{|\vec{r}-\vec{r}_{BL}|>\sigma} dV}$
- Average concentration of a species s in the system = $\phi_s^{avg} = \frac{\int \phi_s(\vec{r},t)dV}{\int dV}$
- Protein partitioning to the BL = $\frac{\phi_s^{BL}}{\phi_s^{out}}$
- Chemical potential of species $s = \frac{\delta \int F dV}{\delta \phi_s}$

S3 CONDENSATE DYNAMICS AND MRNA TRANSCRIPTION IN THE ABSENCE OF ACTIVELY TRANSCRIBING LNCRNAS

To get some baseline expectations, we first study the effect of actively transcribing mRNA on the condensate dynamics, in the absence of any active transcription of lncRNAs. We vary the transcription rate constant k_M of mRNAs and map out the nature of the non-equilibrium steady state and the dynamics of approach. This is described in figure S9A.

As we increase the transcription rate constant k_M, the mRNA concentration at the BL locus at steady state increases (Figure S9B). The amount of protein at the BL locus however initially increases and then decreases (Figure S9B). This is consistent with our expectations from the re-entrant phase diagram described in section S2.1.2. At low k_M, the active transcription of mRNA which depends on the local protein concentration $\phi_P(\vec{r})$ couples with the mRNA-protein interactions to result in a positive feedback loop that helps recruit more protein to the BL. At high k_M, a lot of mRNA is produced in the system at steady state and there is a region in space for which it is unfavorable to form a 2-phase system. This corresponds to the case where enough mRNA is produced to locally dissolve the dense phase of protein due to re-entrant transition. From figures S9B and S9C, we can see that the protein recruitment to the BL locus goes down for high $k_M \geq 0.1$ and the dense phase of protein dissolves. It dissolves from the inside-out and the protein in the system accumulates at the periphery of the BL with most of the RNA being present in the center.

The dynamics of protein recruitment to the BL locus again has two regimes (Figure S9C, Figure S9D). At low k_M, increasing k_M increases the recruitment of protein to the BL at steady state and results in the formation of a stable dense phase of protein (Figure S9D). At high k_M, the mRNA concentrations at the BL can cross $\phi_R^{BL} = \phi_R^c = 0.17$, which locally dissolves the dense phase of protein and results in a short-lived condensate (Figure S9D). This is consistent with prior experimental results relating the amount of mRNA transcribed and condensate lifetimes (23).

S4 SUPPLEMENTAL FIGURES

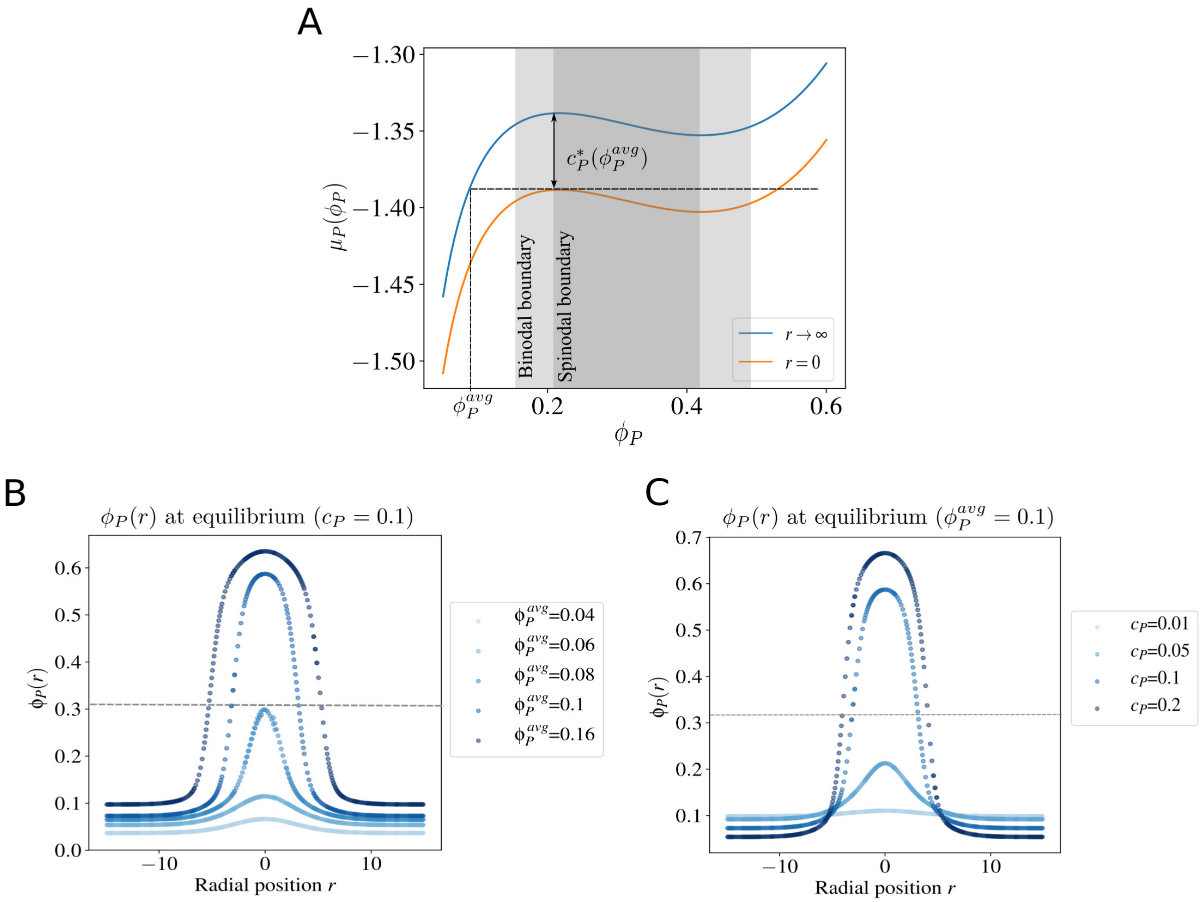

Figure S1: (A) the protein chemical potential μ_P as a function of the protein concentration ϕ_P for $\chi_P = 1.1$, $N_P = 5$ at $r = 0$ and $r \longrightarrow \infty$. The edges of the light gray region represent the values of ϕ_P that correspond to the coexistence concentrations of proteins in the light and dense phase. The dark gray region represents the region of spinodal instability. For a given amount of protein in the system as quantified by ϕ_P^{avg}, $c_P^*(\phi_P^{avg})$ is the depth of the Gaussian chemical potential well required to locally recruit enough protein at the BL at $r = 0$ to cross the spinodal boundary and form a dense phase of protein (B) Protein concentration profile $\phi_P(r)$ along the radial direction for different amounts of protein in the system ϕ_P^{avg} at constant $c_P = 0.1$, obtained by numerically integrating equation 8 till steady state (C) Protein concentration profile of $\phi_P(r)$ along the radial direction at constant $\phi_P^{avg} = 0.1$ for different depths of the chemical potential well c_P, obtained by numerically integrating equation 8 till steady state.

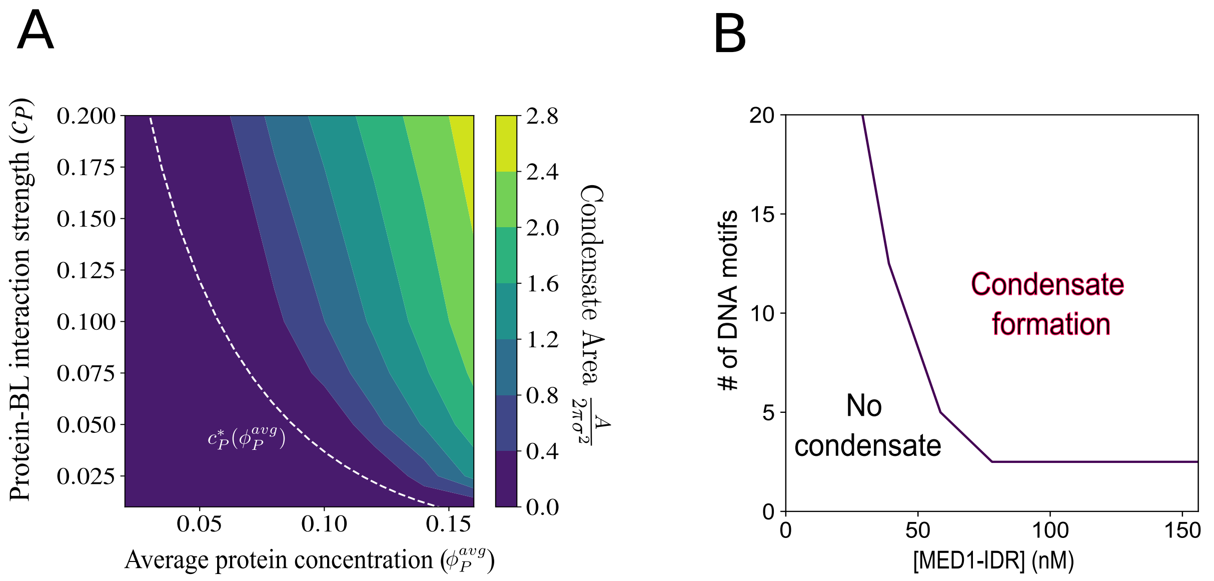

Figure S2: (A) Area of the transcriptional condensate $A/\pi\sigma^2$ at steady state of equation 8 for different amounts of protein in the system (ϕ_P^{avg}) and the strength of protein-BL interaction (c_P). Condensate area A is defined as the area of the region in space where the protein concentration $\phi_P(r) > 0.3$. The free energy parameters used for this plot are $N_P = 5$ and $\chi_P = 1.1$. We can see that the protein does not phase separate for concentrations $\phi_P^{avg} < 0.15$ as these concentrations are below the binodal boundary shown in figure S1A. Increasing c_P promotes condensate formation, resulting in condensate formation for protein concentrations below the binodal boundary. (B) The experimentally measured phase diagram upon varying the number of *Oct4*-DNA motifs and concentration of the transcriptional coactivator Mediator subunit 1 (MED1-IDR) from (7), Figure 4C. The parameters used in this study qualitatively match the experimentally measured phase diagram. MED1-IDR interacts with *Oct4* proteins to form a condensate at regions of chromatin containing *Oct4*-DNA motifs. The concentration of *Oct4* proteins is kept constant for this experiment. Therefore, increasing the number of DNA motifs leads to increased MED1-IDR interaction with the DNA

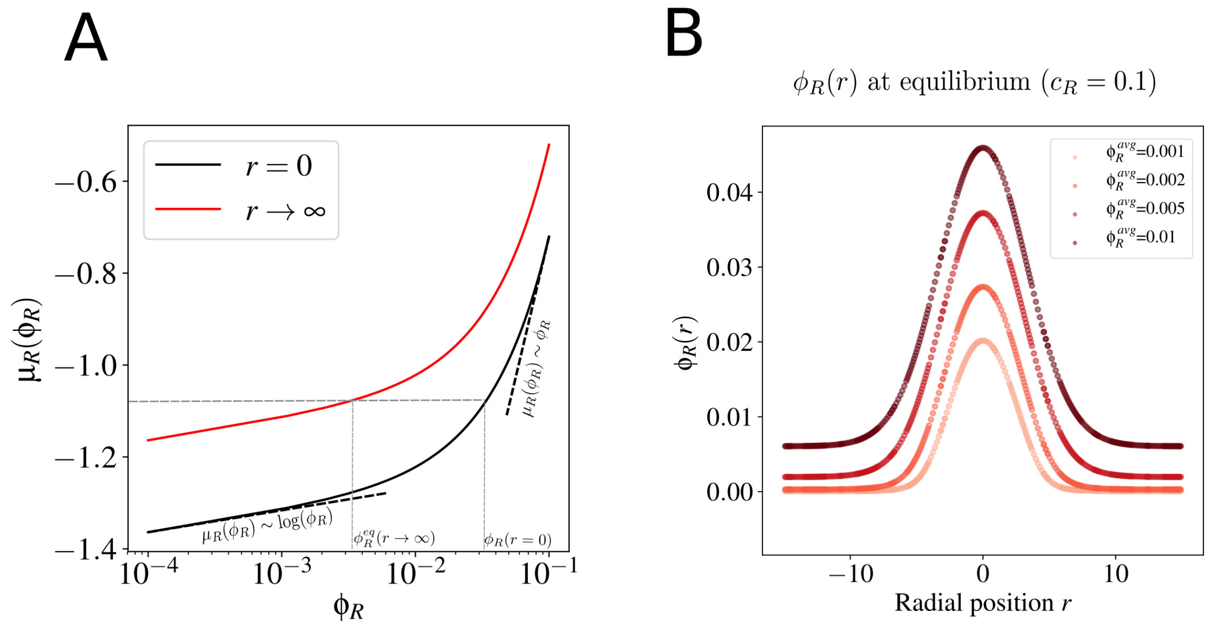

Figure S3: (A) The RNA chemical potential μ_R as a function of the RNA concentration ϕ_R for $c_R = 0.2$, $N_R = 50$, and $\chi_R = 2.0$ at $r = 0$ and $r \to \infty$. The RNA profile at equilibrium $\phi_R^{eq}(r)$ lies within the range $\phi_R^{eq}(r = 0)$ and $\phi_R^{eq}(r \to \infty)$, peaking at $r = 0$ (B) The equilibrium RNA concentration profile $\phi_R^{eq}(r)$ for different average RNA concentrations in the system ϕ_R^{avg} (at fixed $c_R = 0.1$), obtained by numerically integrating equation 24 till steady state

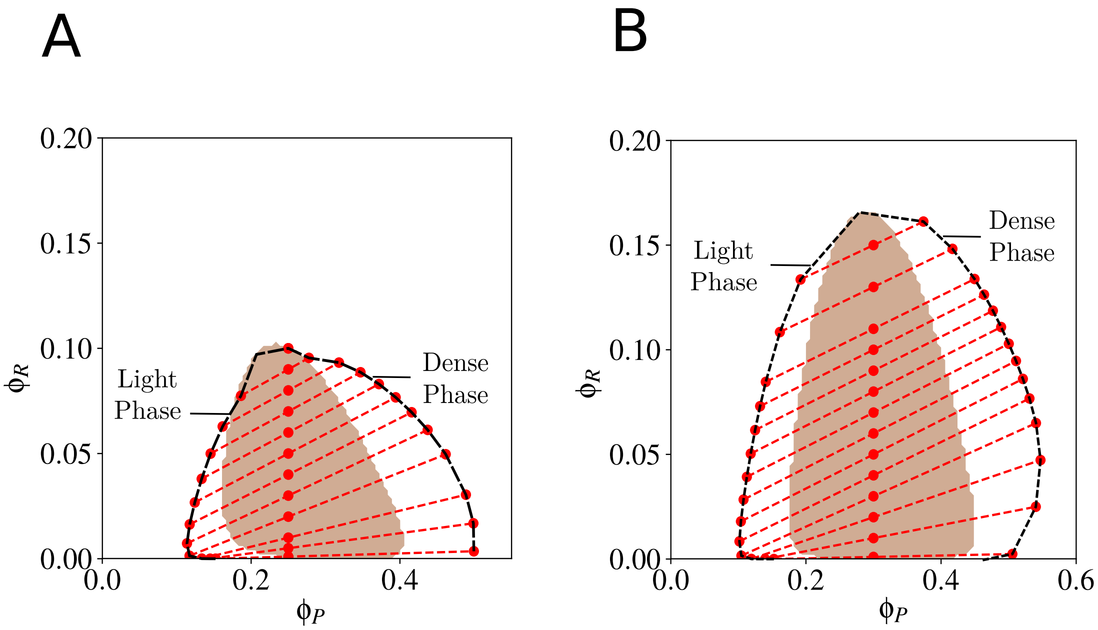

Figure S4: (A) Phase diagram for the Protein-RNA-Solvent ternary system described by $F_{FH}[\phi_P, \phi_R]$ at different protein (ϕ_P) and RNA concentrations (ϕ_R). The parameters used were $N_P = 5, N_R = 50, \chi_P = 1.1, \chi_{PR} = 1.2$ and $\chi_R = 2.0$. The brown region corresponds to the region of spinodal instability. The dotted red lines are coexistence curves that connect a point in the spinodal region to the protein-dense and the protein-light phase compositions (B) Phase diagram for the Protein-RNA-Solvent ternary system described by $F_{FH,mod}[\phi_P, \phi_R]$ using the same set of parameters.

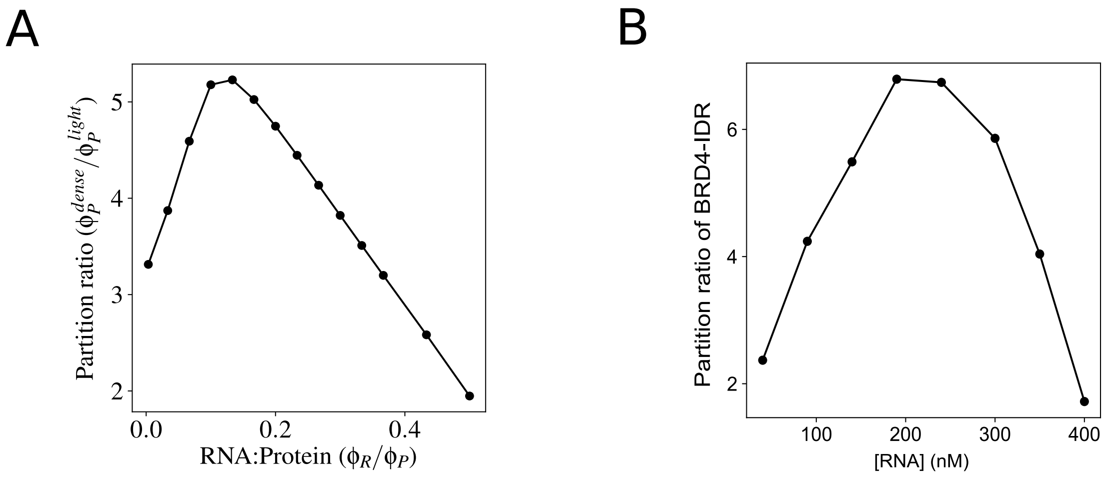

Figure S5: (A) Re-entrant phase diagram representing protein partition ratio $\phi_P^{dense}/\phi_P^{light}$ for a fixed protein concentration ($\phi_P = 0.3$) upon changing the RNA concentration ϕ_R calculated using the free energy $F_{FH,mod}$. The parameters used were $N_P = 5, N_R = 50, \chi_P = 1.1, \chi_{PR} = 1.2$ and $\chi_R = 2.0$. (B) The experimentally measured partition ratio of the transcriptional coactivator BRD4 in the condensate upon varying *Pou5f1* eRNA concentration in (10), figure S2D, exhibiting a re-entrant phase diagram. The parameters used in this study qualitatively match the experimentally measured phase diagram.

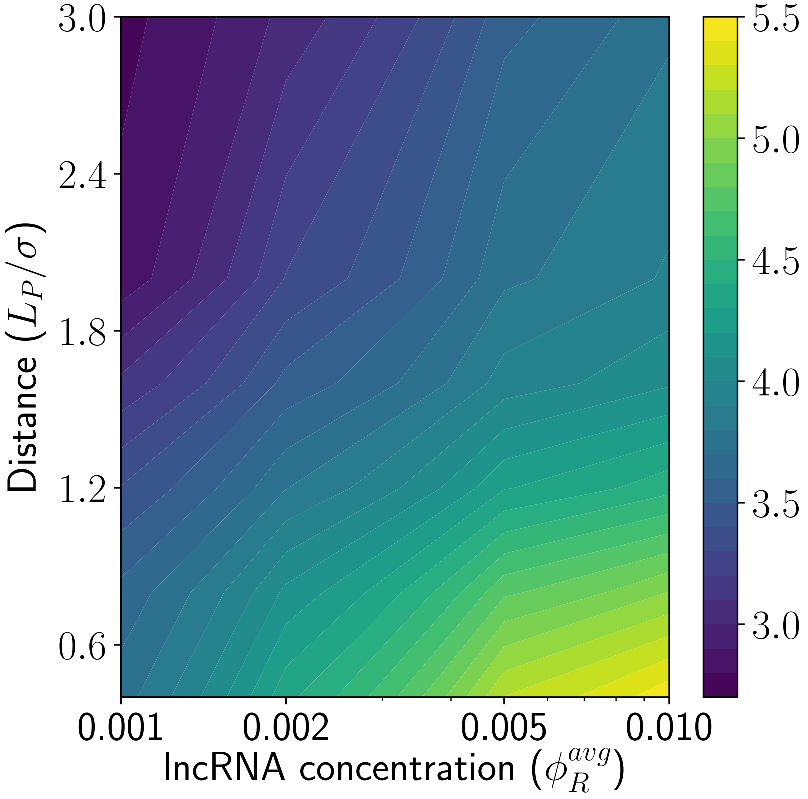

Figure S6: Phase diagram capturing how protein partitioning to the BL varies upon simultaneously varying the distance (L_P/σ) and the amount of lncRNA (ϕ_R^{avg}) in the system. The light white lines indicate contours, where the effects of the lncRNA amounts and the distance can compensate for each other to result in a similar protein partitioning to the BL

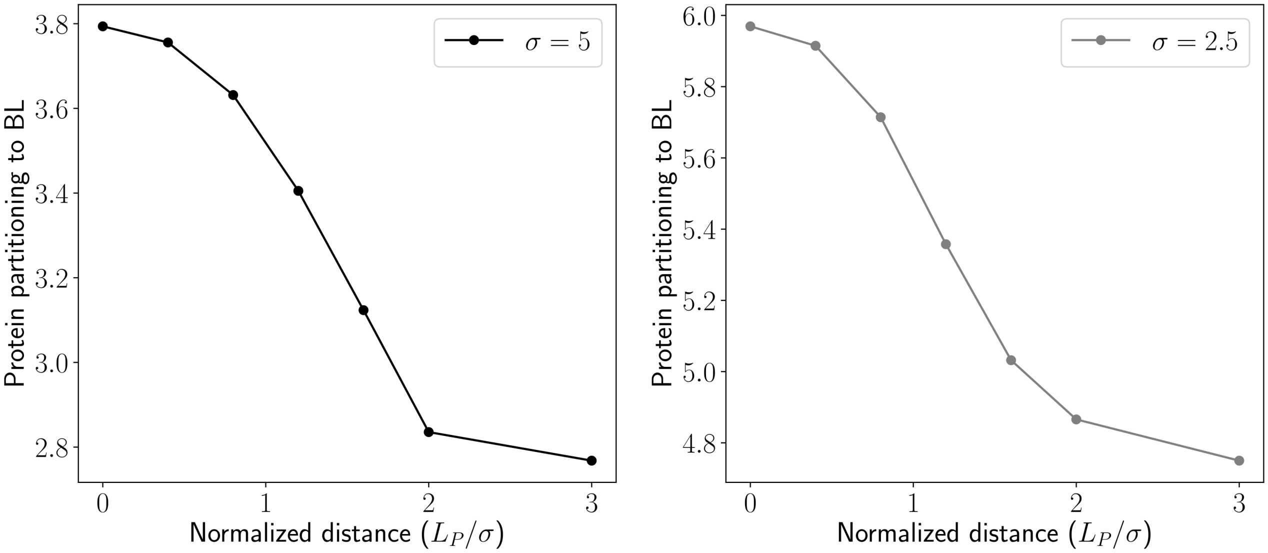

Figure S7: Protein partitioning to the BL upon varying the distance L_P between the BL and RL for two different values of the BL size σ. The trends show a qualitatively similar shape when the distances are graphed as the normalized distance L_P/σ.

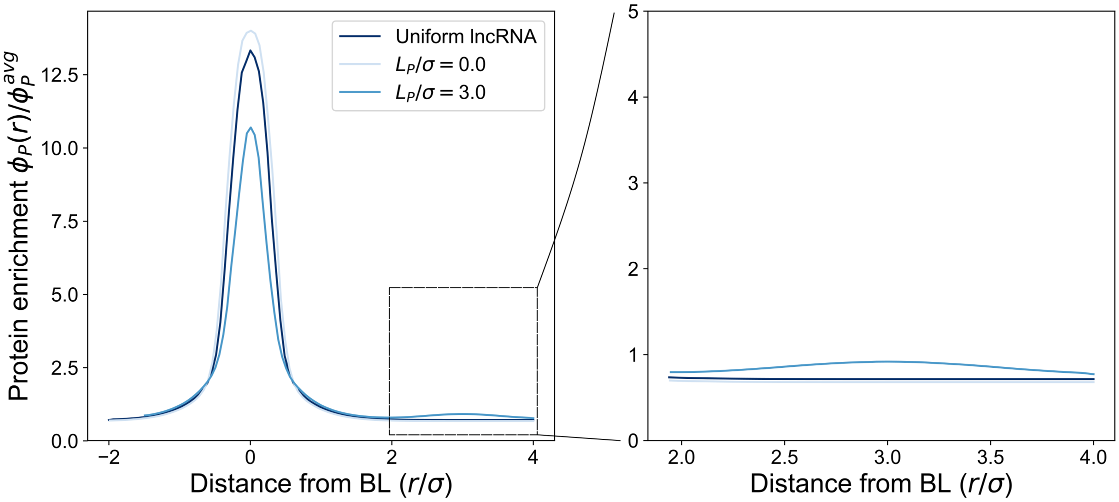

Figure S8: The protein concentration profile along the radial direction in the domain as measured by the protein enrichment $(\phi_P(r)/\phi_P^{avg})$ for three different cases (i) Uniform lncRNA: The are no lncRNA-RL interactions that keep it localized at the RL and it is present initially at a uniform concentration everywhere (ii) lncRNA is localized at the RL which also coincides with the BL (i.e. $L_P/\sigma = 0$) (iii) lncRNA is localized at the RL which is far away from the BL at a distance of $L_P/\sigma = 3$. When the lncRNA is localized at the BL i.e. $L_P/\sigma = 0$, it increases the protein enrichment at the BL compared to the case of uniform lncRNA. However, when the lncRNA at the RL is localized far away from the BL ($L_P/\sigma = 3$), the protein enrichment at the BL is reduced, due to a competition between the BL and the lncRNA accumulated at the RL to recruit the finite amount of available proteins. In this way, lncRNAs present at the RL far away from BLs can act as sponges to sequester away protein.

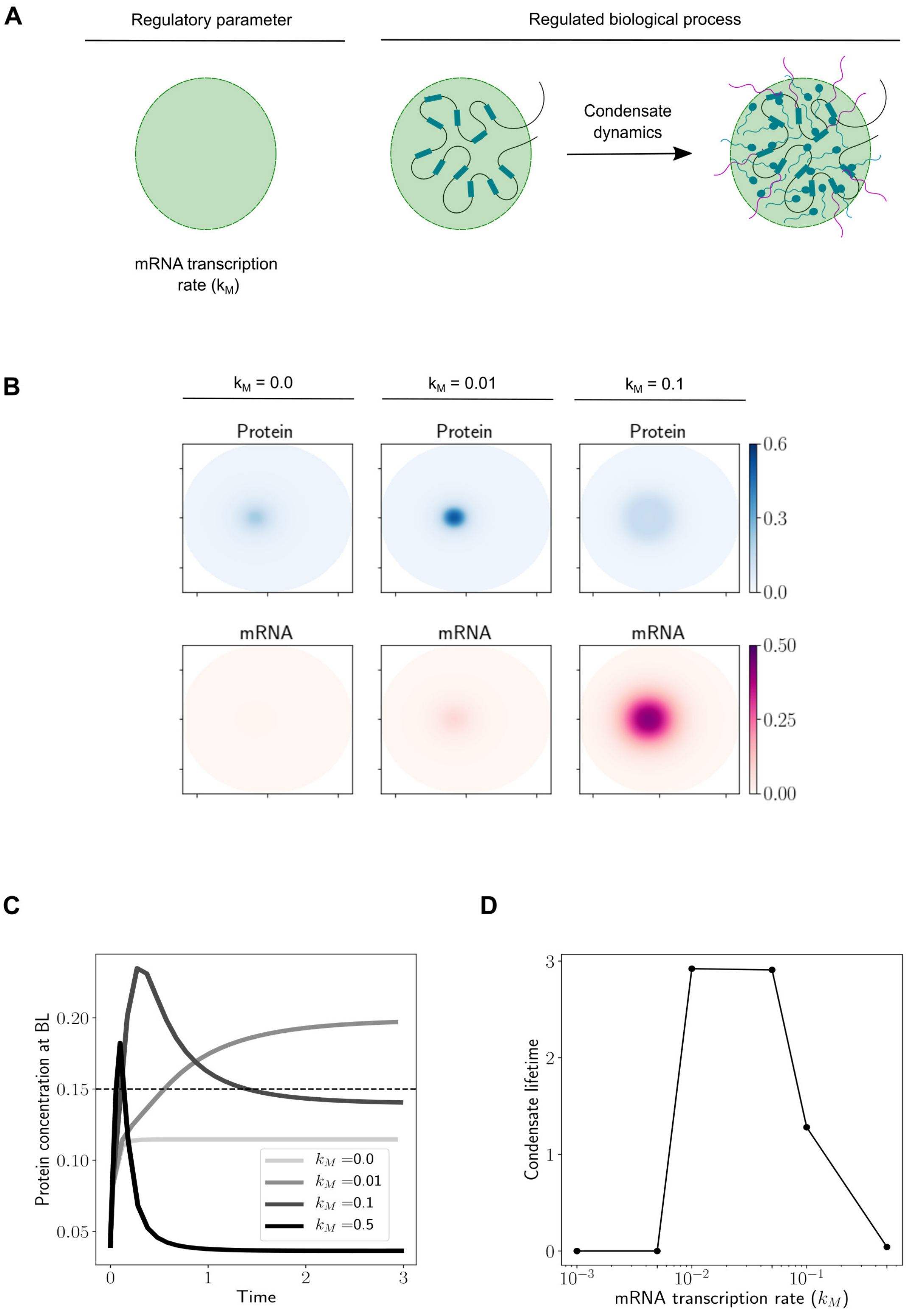

Figure S9: (A) In this figure, we will increase the mRNA transcription rate constant k_M and study how that impacts protein concentration at the BL and condensate lifetimes (B) Steady-state concentration profiles of protein (blue) and RNA (red) at steady state for different values of the transcription rate constant k_M (E) Dynamics of protein concentration at the BL (ϕ_P^{BL}) for different values of k_M. Time is in the dimensionless units of $k_{dM}t$ (D) The dependence of condensate lifetime on k_M. The condensate lifetime is also reported in the dimensionless units $k_{dM}t$, and is defined as the duration of time for which protein concentration at the BL is appreciable i.e. $\phi_P^{BL} > 0.15$.

SUPPORTING REFERENCES

1. Gil, N., and I. Ulitsky, 2020. Regulation of gene expression by cis-acting long non-coding RNAs. *Nature Reviews Genetics* 21:102–117. www.nature.com/nrg.

2. Cai, Z., C. Cao, L. Ji, R. Ye, D. Wang, C. Xia, S. Wang, Z. Du, N. Hu, X. Yu, et al., 2020. RIC-seq for global in situ profiling of RNA–RNA spatial interactions. *Nature* 582:432–437.

3. Beltran, B., D. Kannan, Q. Macpherson, and A. J. Spakowitz, 2019. Geometrical Heterogeneity Dominates Thermal Fluctuations in Facilitating Chromatin Contacts. *Physical Review Letters* 123:208103–208104.

4. Valouev, A., S. M. Johnson, S. D. Boyd, C. L. Smith, A. Z. Fire, and A. Sidow, 2011. Determinants of nucleosome organization in primary human cells. *Nature* 474:516–520.

5. Cho, W. K., J. H. Spille, M. Hecht, C. Lee, C. Li, V. Grube, and I. I. Cisse, 2018. Mediator and RNA polymerase II clusters associate in transcription-dependent condensates. *Science* 361:412–415.

6. Derrien, T., R. Johnson, G. Bussotti, A. Tanzer, S. Djebali, H. Tilgner, G. Guernec, D. Martin, A. Merkel, D. G. Knowles, et al., 2012. The GENCODE v7 catalog of human long noncoding RNAs: analysis of their gene structure, evolution, and expression. *Genome research* 22:1775–1789.

7. Shrinivas, K., B. R. Sabari, E. L. Coffey, I. A. Klein, A. Boija, A. V. Zamudio, J. Schuijers, N. M. Hannett, P. A. Sharp, R. A. Young, and A. K. Chakraborty, 2019. Enhancer Features that Drive Formation of Transcriptional Condensates. *Molecular Cell* 75:549–561.e7. https://doi.org/10.1016/j.molcel.2019.07.009.

8. Boija, A., I. A. Klein, B. R. Sabari, A. Dall'Agnese, E. L. Coffey, A. V. Zamudio, C. H. Li, K. Shrinivas, J. C. Manteiga, N. M. Hannett, B. J. Abraham, L. K. Afeyan, Y. E. Guo, J. K. Rimel, C. B. Fant, J. Schuijers, T. I. Lee, D. J. Taatjes, and R. A. Young, 2018. Transcription Factors Activate Genes through the Phase-Separation Capacity of Their Activation Domains. *Cell* 175:1842–1855.e16. https://doi.org/10.1016/j.cell.2018.10.042.

9. Hohenberg, P. C., and B. I. Halperin, 1977. Theory of dynamic critical phenomena. *Reviews of Modern Physics* 49:435–479.

10. Henninger, J. E., O. Oksuz, K. Shrinivas, I. Sagi, G. LeRoy, M. M. Zheng, J. O. Andrews, A. V. Zamudio, C. Lazaris, N. M. Hannett, et al., 2021. RNA-mediated feedback control of transcriptional condensates. *Cell* 184:207–225.

11. Lin, Y. H., J. P. Brady, J. D. Forman-Kay, and H. S. Chan, 2017. Charge pattern matching as a 'fuzzy' mode of molecular recognition for the functional phase separations of intrinsically disordered proteins. *New Journal of Physics* 19.

12. Quinodoz, S. A., J. W. Jachowicz, P. Bhat, N. Ollikainen, A. K. Banerjee, I. N. Goronzy, M. R. Blanco, P. Chovanec, A. Chow, Y. Markaki, et al., 2021. RNA promotes the formation of spatial compartments in the nucleus. *Cell* 184:5775–5790.

13. Werner, M. S., and A. J. Ruthenburg, 2015. Nuclear Fractionation Reveals Thousands of Chromatin-Tethered Noncoding RNAs Adjacent to Active Genes. *Cell Reports* 12:1089–1098.

14. Knauss, J. L., N. Miao, S.-N. Kim, Y. Nie, Y. Shi, T. Wu, H. B. Pinto, M. E. Donohoe, and T. Sun, 2018. Long noncoding RNA Sox2-ot and transcription factor YY1 co-regulate the differentiation of cortical neural progenitors by repressing Sox2. *Cell death & disease* 9:1–13.

15. Schede, H. H., P. Natarajan, A. K. Chakraborty, and K. Shrinivas, 2022. Organization and regulation of nuclear condensates by gene activity. *bioRxiv* .

16. Jung, C., P. Bandilla, M. von Reutern, M. Schnepf, S. Rieder, U. Unnerstall, and U. Gaul, 2018. True equilibrium measurement of transcription factor-DNA binding affinities using automated polarization microscopy. *Nature communications* 9:1–11.

17. Wu, L., P. Murat, D. Matak-Vinkovic, A. Murrell, and S. Balasubramanian, 2013. Binding interactions between long noncoding RNA HOTAIR and PRC2 proteins. *Biochemistry* 52:9519–9527.

18. Wei, M. T., Y. C. Chang, S. F. Shimobayashi, Y. Shin, A. R. Strom, and C. P. Brangwynne, 2020. Nucleated transcriptional condensates amplify gene expression. *Nature Cell Biology* 22. http://dx.doi.org/10.1038/s41556-020-00578-6.

19. Shi, K., T. Liu, H. Fu, W. Li, and X. Zheng, 2021. Genome-wide analysis of lncRNA stability in human. *PLoS computational biology* 17:e1008918.

20. Gu, B., T. Swigut, A. Spencley, M. R. Bauer, M. Chung, T. Meyer, and J. Wysocka, 2018. Transcription-coupled changes in nuclear mobility of mammalian cis-regulatory elements. *Science* 359:1050–1055.

21. Sabari, B. R., A. Dall'Agnese, A. Boija, I. A. Klein, E. L. Coffey, K. Shrinivas, B. J. Abraham, N. M. Hannett, A. V. Zamudio, J. C. Manteiga, C. H. Li, Y. E. Guo, D. S. Day, J. Schuijers, E. Vasile, S. Malik, D. Hnisz, T. I. Lee, I. I. Cisse, R. G. Roeder, P. A. Sharp, A. K. Chakraborty, and R. A. Young, 2018. Coactivator condensation at super-enhancers links phase separation and gene control. *Science* 361:eaar3958.

22. Muskovic, W., E. Slavich, B. Maslen, D. C. Kaczorowski, J. Cursons, E. Crampin, and M. Kavallaris, 2022. High temporal resolution RNA-seq time course data reveals widespread synchronous activation between mammalian lncRNAs and neighboring protein-coding genes. *Genome Research* 32:1463–1473.

23. Cho, W. K., N. Jayanth, B. P. English, T. Inoue, J. O. Andrews, W. Conway, J. B. Grimm, J. H. Spille, L. D. Lavis, T. Lionnet, and I. I. Cisse, 2016. RNA Polymerase II cluster dynamics predict mRNA output in living cells. *eLife* 5:1–31.
