# Supplementary material for: A model for cis-regulation of transcriptional condensates and gene expression by proximal lncRNAs
Source: Biophys J. 2023 Jun 5;122(13):2757–72. doi: 10.1016/j.bpj.2023.05.032 (PMC10397817; doi:10.1016/j.bpj.2023.05.032)
Supplement: Document S2. Article plus supporting material [file mmc2.pdf]

Biophysical Journal
Article

A model for *cis*-regulation of transcriptional condensates and gene expression by proximal lncRNAs

Pradeep Natarajan,[1] Krishna Shrinivas,[2] and Arup K. Chakraborty[1,3,4,5,6,*]
[1]Department of Chemical Engineering, Massachusetts Institute of Technology, Cambridge, Massachusetts; [2]NSF–Simons Center for Mathematical & Statistical Analysis of Biology, Harvard University, Cambridge, Massachusetts; [3]Department of Physics, Massachusetts Institute of Technology, Cambridge, Massachusetts; [4]Institute of Medical Engineering and Science, Massachusetts Institute of Technology, Cambridge, Massachusetts; [5]Ragon Institute of Massachusetts General Hospital, Massachusetts Institute of Technology and Harvard University, Cambridge, Massachusetts; and [6]Department of Chemistry, Massachusetts Institute of Technology, Cambridge, Massachusetts

ABSTRACT Long noncoding RNAs (lncRNAs) perform several important functions in cells including *cis*-regulation of transcription. Barring a few specific cases, the mechanisms underlying transcriptional regulation by lncRNAs remain poorly understood. Transcriptional proteins can form condensates via phase separation at protein-binding loci (BL) on the genome (e.g., enhancers and promoters). lncRNA-coding genes are present at loci in close genomic proximity of these BL and these RNAs can interact with transcriptional proteins via attractive heterotypic interactions mediated by their net charge. Motivated by these observations, we propose that lncRNAs can dynamically regulate transcription in *cis* via charge-based heterotypic interactions with transcriptional proteins in condensates. To study the consequences of this mechanism, we developed and studied a dynamical phase-field model. We find that proximal lncRNAs can promote condensate formation at the BL. Vicinally localized lncRNA can migrate to the BL to attract more protein because of favorable interaction free energies. However, increasing the distance beyond a threshold leads to a sharp decrease in protein recruitment to the BL. This finding could potentially explain why genomic distances between lncRNA-coding genes and protein-coding genes are conserved across metazoans. Finally, our model predicts that lncRNA transcription can fine-tune transcription from neighboring condensate-controlled genes, repressing transcription from highly expressed genes and enhancing transcription of genes expressed at a low level. This nonequilibrium effect can reconcile conflicting reports that lncRNAs can enhance or repress transcription from proximal genes.

SIGNIFICANCE Long noncoding RNAs (lncRNAs) form a significant part of the human genome but do not code for any proteins. They have many hypothesized functions in the cell, including the regulation of transcription. Transcriptional condensates are assemblies of transcriptional proteins that concentrate at specific genomic sites through phase separation and can regulate transcription. In this study, we propose that lncRNAs can regulate transcription by interacting with proteins in transcriptional condensates to modulate condensate formation. We find that this model can explain some puzzling observations such as conflicting reports of gene activation and repression by lncRNAs, and conservation of genomic distances between lncRNA-coding genes relative to protein-coding genes in metazoans. Experimentally testable predictions that can further explore our model are discussed.

INTRODUCTION

Genes that encode long noncoding RNAs (lncRNAs) outnumber protein-coding genes (PCGs) in the mammalian genome (1,2). lncRNAs are RNAs that have a length of >200 nucleotides and are not translated into any proteins unlike the messenger RNAs (mRNAs). Some well-studied lncRNAs include: NEAT1, which acts as a scaffold in paraspeckles; MALAT1, which regulates the phosphorylation of SR proteins in nuclear speckles; XIST, which is involved in the silencing of the X chromosome; and NORAD, which, promotes genomic stability (3). Except for these and a small number of others, the biological function of the vast majority of lncRNAs is poorly understood.

There is an emerging body of literature that suggests that lncRNAs can regulate transcription in *cis* (4–9). lncRNAs involved in *cis*-regulation usually affect transcription in a

Submitted December 23, 2022, and accepted for publication May 31, 2023.
*Correspondence: arupc@mit.edu
https://doi.org/10.1016/j.bpj.2023.05.032
© 2023 Biophysical Society.
This is an open access article under the CC BY license (http://creativecommons.org/licenses/by/4.0/).

manner that depends on their genomic locus. Transcription of these lncRNAs has a local effect and directly correlates with the transcription of PCGs in genomic and spatial proximity in most cases (10–12). However, recent experiments that perturb lncRNA transcription report conflicting observations on its impact on transcription from neighboring genes. Luo et al. knocked down several divergent lncRNAs in mouse embryonic stem cells using RNAi and observed that gene expression from neighboring PCGs went up in some cases while it went down in others (4). Engreitz et al. suppressed lncRNA transcription in mouse cell lines by knocking out their promoters and reported a similar observation (5). The promoter knockout in some rare cases dramatically decreased gene expression from the neighboring PCG. We do not have a unifying framework to explain these seemingly conflicting observations.

Several experimental studies offer a glimpse into the mechanisms by which lncRNAs regulate transcription in *cis*. lncRNAs can activate gene expression by recruiting the transcriptional coactivator Mediator to neighbor genes (4,9), promote looping between enhancers and promoters (4,8), and recruit histone modifiers to promoter regions of neighboring genes (7). The process of lncRNA transcription has also been hypothesized to activate the transcription of target genes by maintaining enhancers in an active state (13) and by increasing the local concentration of transcription-associated proteins at neighboring promoters (5). The *cis*-regulatory function of lncRNA sequences does not appear to depend strongly on their specific sequences as they are often poorly conserved (6,14) and only weakly selected in humans (15). However, recent evidence suggests that lncRNAs occur at conserved genomic positions relative to orthologous genes (6,16,17). This kind of "positional" conservation rather than sequence conservation motivated us to consider a physical mechanism for *cis*-regulation of gene expression that is agnostic to the specific lncRNA sequence.

Using RNA-DNA SPRITE, Quinodoz et al. demonstrated that mature lncRNAs tend to localize in the vicinity of their coding genomic regions and form their own compartments (18). There is emerging evidence that transcriptional proteins also form their own compartments—called transcriptional condensates—at enhancers and promoters (19–23) and control gene expression from target genes (24,25). These condensates are comprised of biomolecules including transcription factors (19), transcriptional coactivators (20,23), and RNA polymerase II (22,23) that are recruited to enhancers and promoters via a phase-separation mechanism (26). Promoters of PCGs are surrounded mostly by lncRNA-coding genes in their immediate genomic and spatial neighborhood (4,10,11) and many enhancer loci also code for lncRNAs (27,28). The spatial distance between lncRNA-coding loci and promoters and enhancers is of the same order as the size of stable transcriptional condensates (supporting material, section 1). Given this spatial proximity, lncRNAs could interact with components of the transcriptional condensate.

Motivated by these observations, we hypothesized that lncRNAs can regulate transcription in *cis* by interacting with the components of the transcriptional condensate. But what is the nature of this interaction? Recent work suggests that transcriptional coactivators such as Mediator subunit 1 and BRD4 have positively charged disordered domains that can interact with the negatively charged RNA polymer (29) via screened electrostatic interactions. This can result in the condensation of transcriptional proteins driven by the phenomenon of complex coacervation (30–32). A small concentration of RNA promotes condensation driven by electrostatic attraction between the differently charged polymers. However, when the RNA concentrations exceed a value that corresponds to a balance between the total positive and negative charge in the system, this leads to condensate dissolution driven by entropic effects of confining the polymer within the coacervate and electrostatic repulsion between like-charged RNAs (33,34). The nonequilibrium process of RNA transcription can therefore feedback on itself by initially aiding condensate formation and then dissolving it (29). This provides a sequence-agnostic biophysical mechanism that could also be employed by many lncRNAs to control transcription in *cis*.

In this paper, we study how lncRNAs may regulate transcriptional condensates via nonequilibrium phenomena coupled to complex coacervation. We develop a phase-field model for transcriptional regulation by lncRNAs that incorporates known observations about lncRNAs, transcriptional condensates, and interactions between their components, and numerically simulate the model equations. Using this model, we predict that vicinally localized lncRNAs can reduce the threshold protein concentrations required for transcriptional condensate formation and increase protein recruitment to protein-binding loci (BL) on chromatin (e.g., enhancers and promoters). This is a local effect and drops off sharply with the distance between the lncRNA locus (RL) and the BL. Finally, we also predict that local transcription of lncRNAs can aid the formation of transcriptional condensates at PCGs or dissolve it, depending on their level of expression. This in turn has a corresponding effect on transcription from the PCGs. We predict that transcription of proximal lncRNAs enhances transcription from PCGs expressed at a low level, while the same process represses transcription from highly expressed PCGs. Based on these results, we propose that lncRNA transcription can act as a regulatory knob to fine-tune transcription from neighboring genes. Our model provides a mechanistic framework that reconciles conflicting observations about *cis*-regulation of transcription by lncRNAs, provides a possible explanation for how this function can impose genomic constraints on the positions of lncRNA loci, and makes predictions that can be experimentally tested to further explore this mechanism.

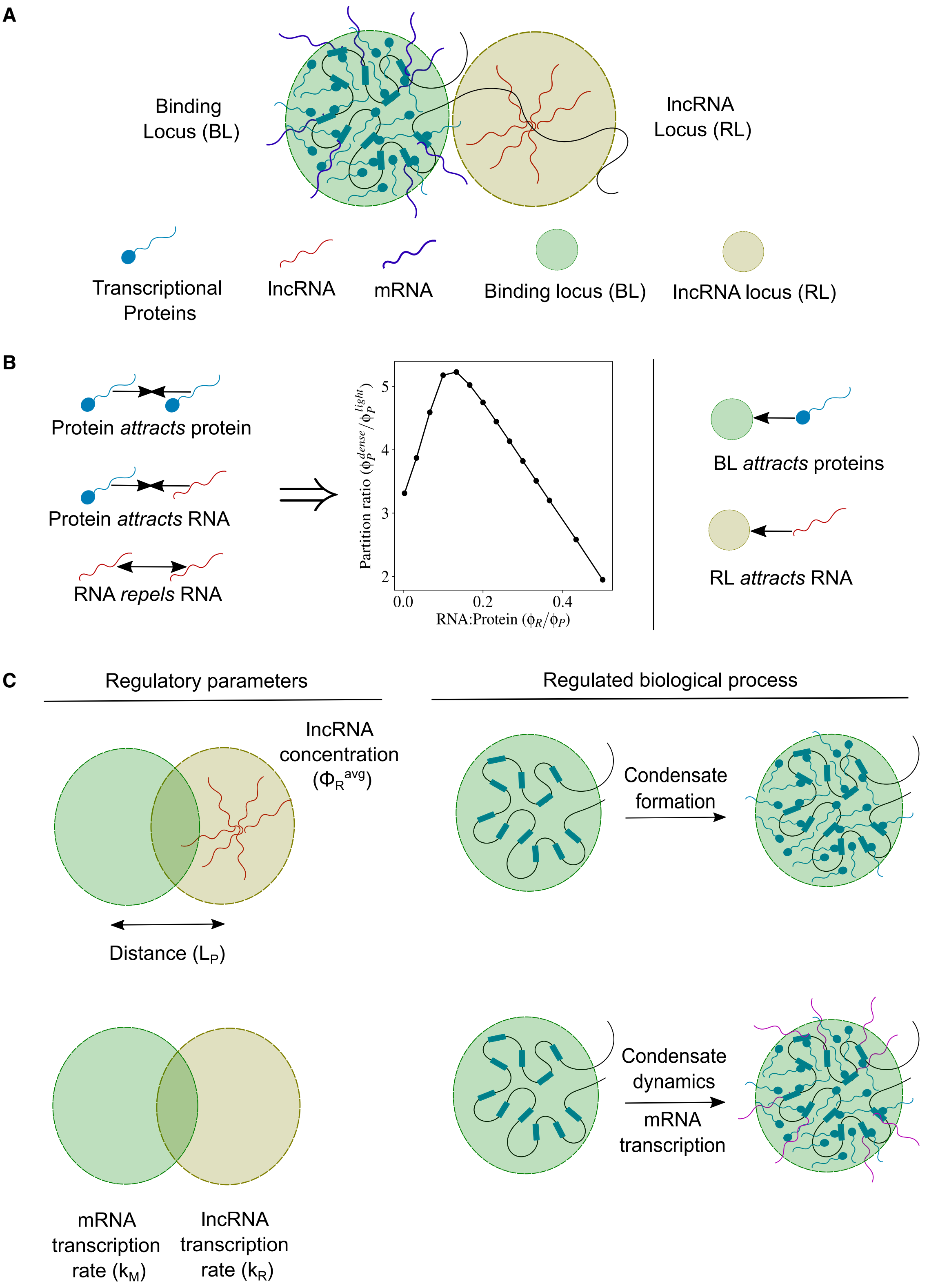

FIGURE 1 (*A*) Cartoon describing the molecular players involved in transcriptional condensate formation. (*B*) Transcriptional proteins attract each other through interactions mediated by their intrinsically disordered domains. RNAs (both lncRNA and mRNA) attract transcriptional proteins through interactions mediated by screened electrostatics or otherwise. RNAs repel each other due to electrostatic repulsion between like-charged polymers. These interactions result in a reentrant phase separation of proteins, where the protein concentration in the protein-dense phase relative to the protein-light phase initially increases and then decreases upon increasing the RNA:Protein concentration ratio. Transcriptional proteins are attracted to binding loci (BL) such as enhancers

(legend continued on next page)

METHODS

Model description

We adopt a continuum phase-field approach to build our model. We have three biomolecular species in our model: the lncRNA, mRNA, and transcriptional proteins (Fig. 1 *A*). We treat the latter as a quasispecies that includes all proteins related to the transcriptional machinery such as transcriptional coactivators and transcription factors. Each of these species is characterized by a concentration field—φ_R (for lncRNA), φ_M (for mRNA), and φ_P (for protein)—that depends on the spatial position. These concentration fields evolve in time governed by partial differential equations that describe 1) the transport of these species in space as a consequence of their interaction with each other and 2) any reactions they might undergo.

To account for the interactions between lncRNA, mRNA, protein, and the chromatin (summarized in Fig. 1 *B*), we write an expression for the free energy of this multicomponent system that comprises the following three terms:

$$F[\varphi_P, \varphi_R, \varphi_M, \vec{r}] = F_{FH}[\varphi_P, \varphi_R, \varphi_M] + F_{BL}[\varphi_P, \vec{r}] + F_{RL}[\varphi_R, \vec{r}] + F_{surf}[\varphi_P] \quad (1)$$

$F_{FH}[\varphi_P, \varphi_R, \varphi_M]$ is a Flory-Huggins free energy that captures the self and cross interactions between transcriptional proteins, lncRNA, and the mRNA. A detailed expression for this free energy is given in supporting material, section 2.1. In brief, the parameters χ_P, χ_R, and χ_{PR} in the equations of supporting material, section 2.1 correspond to the Flory-Huggins parameters that capture the mean-field pairwise interactions strength between protein-protein, RNA-RNA, and protein-RNA species, respectively. This free energy captures the following three biologically relevant interactions: 1) attractive protein-protein interactions, 2) repulsive RNA-RNA interactions, and 3) attractive protein-RNA interactions. We assign the protein-protein interactions to be attractive (i.e., $\chi_P < 0$) motivated by the observation that many transcriptional proteins contain intrinsically disordered regions (IDRs) that promote the formation of transcriptional condensates (19,21). The attractive interactions between IDRs arise from various interactions at the amino acid level such as electrostatic (35,36), pi-pi (37), cation-pi (38), and hydrophobic (35) interactions. Interactions between all the RNA species in our model are chosen to be repulsive (i.e., $\chi_R > 0$) motivated by the fact that lncRNA and mRNA species are both negatively charged polymers that can interact via screened electrostatic repulsion. Finally, the protein-mRNA and protein-lncRNA interactions are attractive (i.e., $\chi_{PR} < 0$) in our model, motivated by the observation that many transcriptional coactivators contain positively charged IDRs (29) and transcription factors contain positively charged RNA-binding regions (39) that can bind to negatively charged RNA.

The term $F_{BL}[\varphi_P, \vec{r}]$ captures the interaction free energy of transcriptional proteins with regions of attractive chromatin that promote condensate formation (21). We call these regions of attractive chromatin such as specific enhancers, superenhancers, or promoters the binding locus, or BL. We can write the free energy between transcriptional protein concentration field $\varphi_P(\vec{r})$ at position $\vec{r}$ and its BL located at position $\vec{r}_{BL}$ using a Gaussian function that has a spatial extent of σ_{BL} and a net strength of attraction c_P as shown in Eq. 2.

$$F_{BL}[\varphi_P, \vec{r}] = -c_P e^{-\frac{|\vec{r}-\vec{r}_{BL}|^2}{\sigma_{BL}^2}\varphi_P} \quad (2)$$

If the BL is compact in space, σ_{BL} is small, and if the BL is more spread out in space, then σ_{BL} is large. c_P is a coarse-grained parameter that captures the free energy of binding per protein molecule. Given the spatial extent of the BL, a larger number of protein-binding sites within the BL or stronger protein binding to each protein binding site would result in a higher c_P. While beyond the scope of this work, given a polymer model of the BL at the appropriate scale, it should be possible to derive the specific dependence of the coarse-grained parameters σ_{BL} and c_P on the length of the BL, the density of binding sites, and the binding strength of individual proteins to binding sites on the BL. If we have additional nonspecific DNA elements in the vicinity of the BL, they can compete with the BL to recruit protein. However, we have not considered this effect in our model.

Finally, there is also emerging evidence that many lncRNAs localize in close proximity to their genomic loci (18). We refer to the genomic loci that code for lncRNAs as the lncRNA locus, or RL, for the rest of this paper. There are many mechanisms that could facilitate attractive interactions between lncRNAs and their RL—these include tethering by transcription factors such as YY1 (40,41) or by RNA polymerase (42). Irrespective of the mechanism, we can write a free energy between the lncRNA concentration field $\varphi_R(\vec{r})$ at position $\vec{r}$ and its RL located at position $\vec{r}_{RL}$ using a Gaussian function that has a range σ_{RL} and strength of attraction c_R:

$$F_{RL}[\varphi_R, \vec{r}] = -c_R e^{-\frac{|\vec{r}-\vec{r}_{RL}|^2}{\sigma_{RL}^2}\varphi_R} \quad (3)$$

The term $F_{surf}[\varphi_P] = \frac{\kappa}{2}|\nabla\varphi_P|^2$ is a surface tension term that penalizes sharp gradients in protein concentration, with κ being the strength of this energy penalty. This term is not particularly important for our results but ensures that any phase separation is accompanied by smooth boundaries between phases.

The rationale behind the choices of parameters associated with the different free energy terms of Eq. 1 are described in detail in supporting material, sections 2.1.1, 2.1.2, and 2.1.3, and are summarized in Table S1. The purpose of the model is to qualitatively understand the emergent consequences of the interplay between phase separation of proteins caused by protein-protein and protein-BL interactions, the reentrant phase diagram that arises due to protein-protein and protein-RNA interactions, and the localization of lncRNA species. The model parameters have not been chosen to make quantitative predictions about any particular system, but have been chosen to respect the constraints imposed by previous biophysical measurements. These chosen parameters qualitatively match experimentally measured phase diagrams of transcriptional proteins with DNA that form the BL (21) as shown in Fig. S2 and with RNA species (29) as shown in Fig. S5.

Using this model, we hope to answer the following two questions: 1) How does a lncRNA localized near a BL affect the formation of transcriptional condensates? 2) How does an actively transcribed lncRNA affect mRNA transcription from a nearby BL? Specifically, we look at how the amount of lncRNA ($\varphi_R^{avg} = \int \varphi_R(\vec{r})dV$), the distance between the BL and the RL ($L_P = |\vec{r}_{BL} - \vec{r}_{RL}|$), and the rate of lncRNA transcription at the RL (k_R) relative to the mRNA (k_M) affect the above processes (Fig. 1 *C*).

Dynamics of condensate formation

In this section, we develop a model to answer the first question: How does a lncRNA localized near a BL affect the formation of transcriptional

and promoters. lncRNAs can localize near their genomic loci, which we call the lncRNA locus (RL). (*C*) Cartoon describing the different regulatory parameters investigated in this study along with the biological process that they regulate. The amount of lncRNA (as measured by the average lncRNA concentration φ_R^{avg}) and the distance (L_P) between the RL and the BL can affect condensate formation at the BL. The relative magnitudes of the mRNA transcription rate constant (k_M) at the BL and the lncRNA transcription rate (k_L) at the lncRNA locus affect the dynamics of the protein condensate and therefore mRNA transcription from the BL.

condensates? To do this, we consider a situation where there is a uniform concentration of transcriptional proteins everywhere in space at time $t = 0$. The lncRNA is spatially localized at the RL according to the concentration profile $\varphi_R^{eq}(\vec{r})$ described in supporting material, section 2.1.3. There is no active transcription of mRNA happening at the BL and the free energy, in this case, does not depend on φ_M, i.e., $F[\varphi_P, \varphi_R, \vec{r}] = F[\varphi_P, \varphi_R, \varphi_M = 0, \vec{r}]$. As time progress, the protein starts to accumulate at the BL driven by the attractive protein-protein and protein-BL interactions. We define a condensate as a region in space where the protein concentration is above a threshold value, which is set by the free energy parameters (refer to Table S1 for details on this threshold value). The lncRNA localized at the RL can perturb the dynamics of condensate formation at the BL depending on its amount (φ_R^{avg}) and how far away it is (L_P).

Condensate formation happens over the timescale of a few minutes (24), which is much shorter compared with the half-lives of most lncRNAs (43) and proteins (44), which can span hours. Therefore, we assume that the protein and lncRNA are stable over our simulation of condensate formation. For conserved species, the spatiotemporal dynamics of concentrations are such that the molecules move down gradients in chemical potential. The coupled dynamics of the concentrations $\varphi_P(\vec{r})$ and $\varphi_R(\vec{r})$ can be captured using the following model B equations (45), with D_P and D_R representing the protein and lncRNA diffusivities, respectively:

$$\frac{\partial \varphi_P(\vec{r})}{\partial t} = \vec{\nabla}.(D_P \varphi_P(\vec{\nabla}\mu_P)) \tag{4}$$

$$\frac{\partial \varphi_R(\vec{r})}{\partial t} = \vec{\nabla}.(D_R \varphi_R(\vec{\nabla}\mu_R)) \tag{5}$$

where the chemical potentials for the protein and the RNA species are given by $\mu_P = \frac{\delta \int F[\varphi_P, \varphi_R, \vec{r}]dV}{\delta \varphi_P}$ and $\mu_R = \frac{\delta \int F[\varphi_P, \varphi_R, \vec{r}]dV}{\delta \varphi_R}$, respectively.

Dynamics of transcription

In this section, we develop a model to answer the second question: How does an actively transcribed lncRNA affect mRNA transcription from a nearby BL? BLs with active mRNA transcription are often not isolated but located in neighborhoods that contain other actively transcribing RNAs including lncRNAs. Transcription of neighboring lncRNAs can potentially couple to the dynamics of mRNA transcription specifically by modulating protein recruitment to the BL and transcriptional condensate formation, thereby regulating gene expression.

Active transcription and depletion of RNAs that consume ATP can alter the local RNA concentrations and push the system far out of equilibrium. The rate of mRNA transcription must depend on both the local concentration of transcriptional proteins and the coding DNA. We take into account the local coding-DNA concentration through an effective rate constant ($k_M(\vec{r})$ in Eq. 7) that is a Gaussian function in space centered at the BL, reflecting the concentration of these genes at the BL. In addition to the spatially varying rate constant, the mRNA transcription rate has a simple first-order dependence on φ_P (Eq. 7), reflecting the activating effect of transcriptional proteins. To be general, we assume that lncRNA transcription is not controlled by the same transcriptional proteins and its rate is independent of φ_P (Eq. 8). The lncRNA transcription rate ($k_R(\vec{r})$ in Eq. 8) is also modeled as a Gaussian function in space centered at the RL to reflect its transcription from its coding DNA, which is localized at RL. Using this function for both the coding-DNA concentrations is a simple approximation if we assume the genomic region to be a Gaussian polymer. The values σ_R and σ_M reflect the spatial extents of the DNA that code for the lncRNA and the mRNA respectively. In addition to the spatially varying production rates of the species, we also have a simple first-order decay of the mRNA and lncRNA species throughout space with rate constants of k_{dM} (Eq. 7) and k_{dR} (Eq. 8), respectively. Using these arguments, we construct the following model where the reaction-diffusion dynamics of the lncRNA affects mRNA transcription by perturbing the dynamics of the protein field $\varphi_P(\vec{r}, t)$:

$$\frac{\partial \varphi_P(\vec{r},t)}{\partial t} = \vec{\nabla}.(D_P \varphi_P(\vec{\nabla}\mu_P)) \tag{6}$$

$$\frac{\partial \varphi_M(\vec{r},t)}{\partial t} = D_M \nabla^2 \varphi_M + \underbrace{k_M e^{-\frac{|\vec{r}-\vec{r}_{BL}|^2}{\sigma_{BL}^2}}}_{k_M(\vec{r})} \varphi_P - k_{dM}\varphi_M \tag{7}$$

$$\frac{\partial \varphi_R(\vec{r},t)}{\partial t} = D_R \nabla^2 \varphi_R + \underbrace{k_R e^{-\frac{|\vec{r}-\vec{r}_{RL}|^2}{\sigma_{RL}^2}}}_{k_R(\vec{r})} - k_{dR}\varphi_R \tag{8}$$

For this study, we vary the magnitudes of the lncRNA production rate (k_R) and mRNA transcription rate constant (k_M), and investigate how that affects condensate dynamics and mRNA expression. Table S2 summarizes the diffusivities and rate constants used in simulations and supporting material, section 2.2.3 rationalizes the choice of these parameters.

A difference between the model described by Eqs. 4, 5, 6, 7, and 8 is the mechanism of lncRNA localization. In this model, the lncRNA production rate is peaked at the RL. Therefore, the lncRNA concentration is highest at the RL and decreases with distance due to diffusion and degradation. Another important difference is that Eqs. 6–8 define processes far out of equilibrium, and not dynamics down a free energy gradient.

Numerical simulation of model equations

The above partial differential equations were numerically solved using a custom Python code, available here. The Zenodo-generated DOI for the same is https://doi.org/10.5281/zenodo.8032346. This code uses the finite volume solver Fipy developed by the National Institute of Standards and Technology (46). All simulations in this paper were done in a 2D circular domain of radius 15 units, with a circular discrete mesh. The spatially discretized partial differential equations were solved for each incremental time step with adaptive time stepping to pick smaller or larger time steps depending on how quickly or slowly the concentration fields change. A grid size of $\Delta r = 0.1$ and a typical time step size on the scale of $\Delta t = 0.2$ worked well for the simulations. Simulations were run for a duration of 2000 time steps, which was sufficient for the system to reach a steady state.

For the dynamics of condensate formation with localized lncRNA, the equilibrium concentration profile of lncRNA was obtained as described in supporting material, section 2.1.3, which was then used as the initial condition for simulating the dynamics. For all simulations, a uniform protein concentration profile was used as the initial condition, with a value of $\varphi_P^{avg} = 0.04$ unless stated otherwise. This corresponds to a regime where the protein does not form a condensate by itself and requires lncRNA for condensate formation and this value was chosen to illustrate the effects of lncRNAs more sharply. The initial concentration of mRNA everywhere was set to $\varphi_M = 0$. The no-flux Neumann boundary condition was applied to all species at the domain boundaries.

Analyses

Numerical simulations yield the full concentration profiles of the protein $\varphi_P(\vec{r}, t)$, lncRNA $\varphi_R(\vec{r}, t)$, and mRNA $\varphi_M(\vec{r}, t)$ at all times t. Once we have these data, we can calculate quantities such as the concentration of a species at the BL, the partition ratio of species at the BL, the average concentration of the species in the system, and the chemical potential of the species. The precise formula for each of these quantities is described in supporting material, section 2.3.

RESULTS

Proximal lncRNAs can enhance recruitment of transcriptional proteins to superenhancers and promoters

Condensate formation by transcriptional proteins at BL is driven cooperatively by protein-chromatin binding interactions and attractive protein-protein interactions mediated by their disordered domains (21). When the concentration of transcriptional proteins crosses a threshold, there is a sharp increase in protein concentration at the BL due to phase separation and condensate formation driven by these two interactions.

As the first step, we wanted to understand how lncRNAs localized near a BL can affect condensate formation. The amount of lncRNA (φ_R^{avg}) is an important regulatory parameter that controls the magnitude of this effect. We started with $\varphi_R^{avg} = 0$ (no lncRNA) and progressively increased the amount of lncRNA in the system. We numerically simulated the model described by Eqs. 4 and 5 by varying the protein concentration in the system (φ_P^{avg}) and quantified the protein partitioning to the BL at steady state (Fig. 2 *A*). We find that vicinally localized lncRNAs consistently enhance protein partitioning to the BL compared with the base case where there is no lncRNA (Fig. 2 *B*). Protein partitioning to the BL increases sharply upon increasing the protein concentration before reaching a plateau. This sharp increase is due to the phase separation of the proteins, and we can define a threshold value of protein concentration for which a condensate, i.e., a dense phase of protein (with concentration $\geq \varphi_P^{light}$), starts to appear at the BL. We find that lncRNAs localized near the BL can reduce the transcriptional protein concentration thresholds that are required for phase separation and condensate formation (Fig. 2 *B*). Thus, attractive interactions between transcriptional proteins and lncRNAs localized in the vicinity mediated by screened electrostatic interactions or otherwise can add an additional layer of cooperativity along with protein-chromatin and protein-protein interactions to aid condensate formation.

There exists a regime of protein concentrations for which lncRNA is necessary for condensate formation (Fig. 2 *B*) and a condensate does not form in the absence of lncRNAs (Fig. 2 *C*). In this regime, the additional layer of cooperativity added by the lncRNA-protein attractive interactions is necessary for condensate formation. This observation can explain why knocking down lncRNAs can sometimes have a dramatic effect on mRNA transcription from neighboring genes (5). A transcriptional condensate simply does not form to initiate transcription. At large protein concentrations where condensate formation happens even in the absence of lncRNAs, the presence of lncRNAs in the vicinity can still enhance protein recruitment to the BL (Fig. 2 *B*). In all cases, protein partitioning to the BL directly correlates with the lncRNA concentration at the BL (Fig. 2 *D*).

The dynamics of protein recruitment to the BL dictates the speed of cellular response to an external stimulus by activating gene expression. Therefore, we wanted to understand how different amounts of proximally localized lncRNA (φ_R^{avg}) affect the dynamics of protein recruitment to the BL. We graphed the evolution of protein concentration at the BL with time (Fig. 2 *E*) and find that increasing the amount of lncRNA has two distinct effects, which point to two distinct regulatory roles: 1) higher amounts of lncRNA can increase the initial rate of protein recruitment to the BL (Fig. 2 *F*), speeding up the response time between the cells receiving a stimulus and forming transcriptional condensates; and 2) higher amounts of lncRNA can increase the protein concentration at the BL at steady state (Fig. 2 *E*), increasing the strength of response to the stimulus. In this way, a cell can regulate the speed and magnitude of protein recruitment to the BL by using the amounts of proximally localized lncRNAs as a tunable knob.

To shed light on the mechanistic basis of these effects, we graphed the chemical potential profiles of the protein at initial times (Fig. 2 *G*). The chemical potential at initial times has a shape of a Gaussian well, which is what we would expect based on the attractive protein-chromatin interactions at the BL described by Eq. 2. Increasing the amount of lncRNA (φ_R^{avg}) in the vicinity of the BL has two effects: it makes the well deeper and broader. The presence of lncRNAs near the BL and their attractive interactions with the protein provides a free energy benefit in addition to the protein-chromatin interactions, which translates to a deeper chemical potential well. A deeper well means that the chemical potential gradients are steeper, resulting in higher fluxes of the protein and a faster speed of protein recruitment to the BL. Spatial overlap between the BL and the localized lncRNA results in a broader effective region in space that attracts the protein. A broader well leads to increased overall protein recruitment to the BL, because a broader well can hold more overall amount of protein.

In summary, the two ingredients—1) localization of lncRNA near BL and 2) attractive interactions between lncRNAs and proteins, possibly due to complementary charges and the resultant screened electrostatic interaction—can enhance the magnitude and dynamics of protein recruitment to the BL.

Proximal lncRNAs migrate to the BL to help recruit transcriptional proteins while distal lncRNAs compete with the BL for these proteins

Since lncRNAs localize at the RL, their concentration profile is peaked at the center of the RL and decays over a length scale of $\sigma_{RL} = \sigma$ (Fig. S3 B). The distance (L_P) between the BL and the RL is an important regulatory parameter that can affect local lncRNA concentration at the BL and therefore affect protein recruitment (Fig. 3 *A*). Therefore, we looked at how the distance (L_P) affects the dynamics of protein recruitment to the BL and condensate formation. It is also important to note that the lncRNA concentration

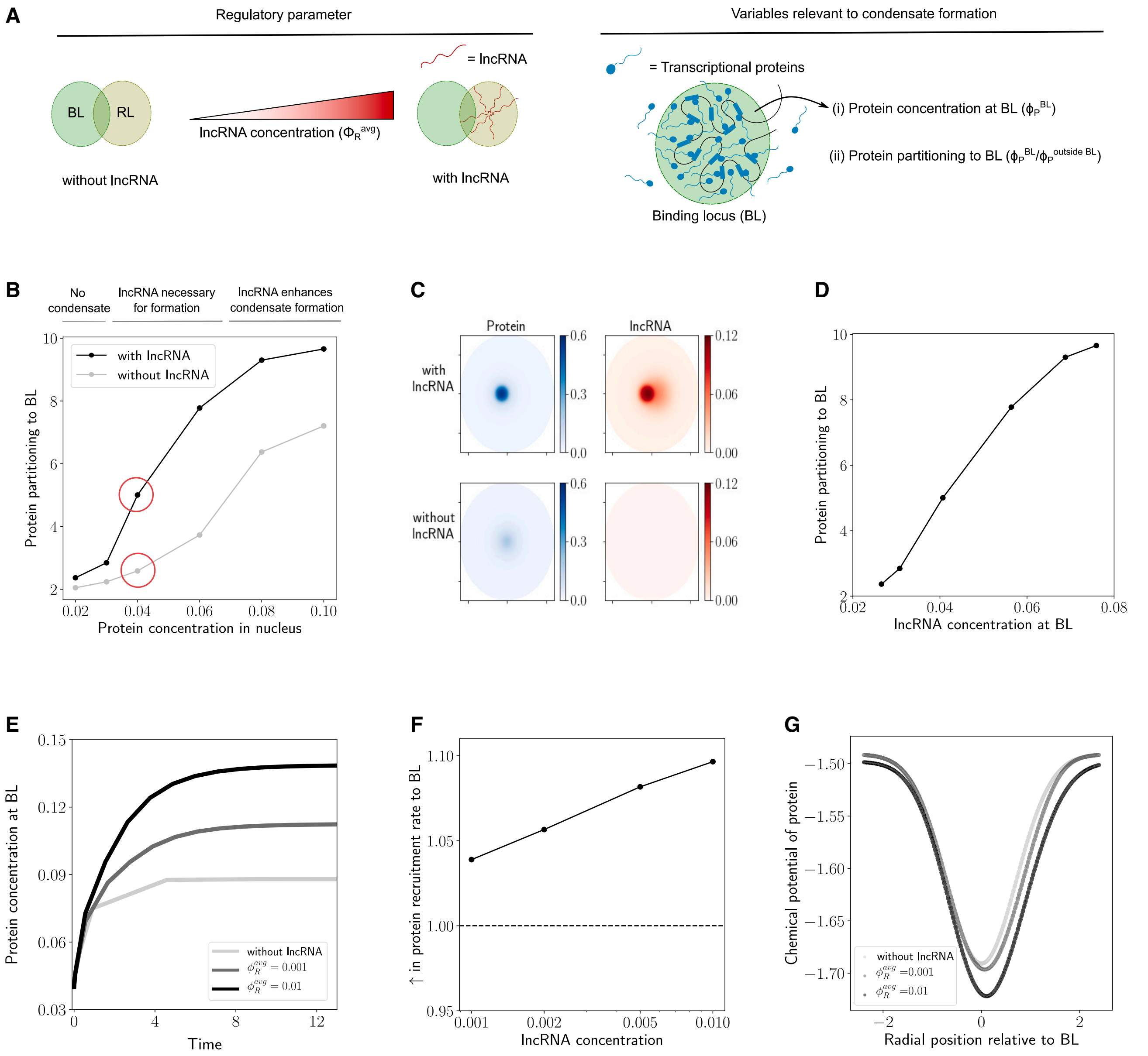

FIGURE 2 (*A*) In this figure, results are shown for what happens when we increase the lncRNA concentration (φ_R^{avg}) starting from a case without lncRNA ($\varphi_R^{avg} = 0$). We quantify protein recruitment to the BL using the following two metrics: the protein concentration (φ_P^{BL}) in the BL and the protein partitioning to the BL ($\varphi_P^{BL}/\varphi_P^{out}$). The distance between the loci was set to $L_P = 0.8\sigma$. (*B*) Change in protein partitioning to the BL upon increasing the amount of protein in the nucleus. A protein condensate is formed when there is a sharp increase in protein partitioning to the BL. The gray curve corresponds to the case without lncRNA and the black curve corresponds to a case with a lncRNA amount of $\varphi_R^{avg} = 0.01$. The concentration profiles of protein and lncRNA in space are depicted for the circled data points in (*C*). (*C*) The protein and lncRNA concentration profiles are illustrated for the case with and without lncRNA. The average protein concentration in the nucleus for both cases is $\varphi_P^{avg} = 0.04$. (*D*) The relationship between protein partitioning to the BL and the average lncRNA concentration in the BL for different amounts of protein in the nucleus. (*E*) Dynamics of protein recruitment: protein concentration in the BL versus time for different amounts of lncRNA. The time (t) is reported in dimensionless units as tD_P/R^2. D_P is the diffusion coefficient of the protein and R is the radius of the nucleus. (*F*) The initial rate of protein recruitment to the BL for different amounts of lncRNA. The initial rate of protein recruitment is the slope of the graphs in (*E*) at $t = 0$. They are reported in this figure as a ratio relative to the case with no lncRNA ($\varphi_R^{avg} = 0$). (*G*) Chemical potential of protein versus radial position at $t = 0$ for different amounts of lncRNA. The radial position is measured relative to the center of the BL, with the origin being the center.

profile can dynamically change due to protein accumulation at the BL, leading to interesting and nontrivial dynamics. We numerically simulated the dynamics described by Eqs. 4 and 5 by varying the distance $L_P = |\vec{r}_{BL} - \vec{r}_{RL}|$ between the loci. We then quantified the protein partitioning to the BL at equilibrium.

Simulations using different values of σ reveal that the protein partitioning to the BL primarily depends upon the

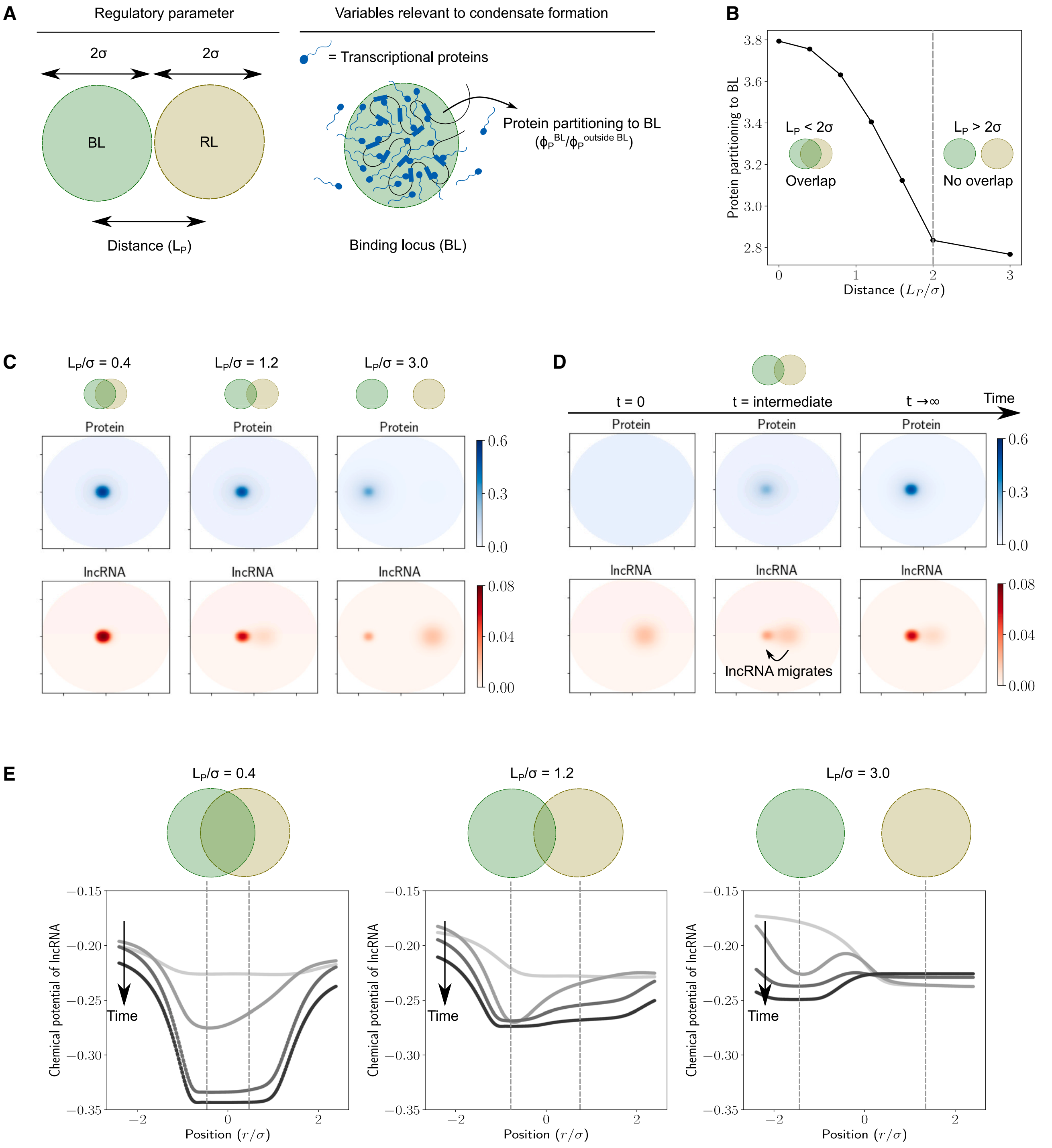

FIGURE 3 (*A*) In this figure, we change the distance (L_P) between BL and RL and quantify the protein partitioning to the BL ($\varphi_P^{BL}/\varphi_P^{out}$). The amount of lncRNA was set to $\varphi_R^{avg} = 0.001$ and the average protein concentration to $\varphi_R^{avg} = 0.04$. (*B*) Condensate formation: protein partitioning to the BL upon changing the distance between BL and RL. The distance is reported as the normalized value L_P/σ. When $L_P < 2\sigma$, there is some overlap between the BL and the RL. When $L_P > 2\sigma$, there is no appreciable overlap between the BL and the RL. (*C*) Concentration profiles of protein and lncRNA at equilibrium for different values of the normalized distance L_P/σ. (*D*) Dynamics of protein recruitment: snapshots of protein and lncRNA concentration profiles at different times. At $t = 0$, the protein is present at a uniform constant concentration everywhere, while the lncRNA has a concentration profile peaked at the center of the RL. The distance between the RL and BL is $L_P = 1.2\sigma$, which corresponds to the case with partial overlap. (*E*) The chemical potential of lncRNA versus radial position at $t = 0$ for different amounts of lncRNA. The radial position is measured relative to the midpoint of the line connecting the BL and RL, with the origin being the midpoint.

normalized distance L_P/σ (Fig. S7). Protein partitioning to the BL sharply decreases upon increasing the normalized distance L_P/σ (Fig. 3 *B*). When the BL and the RL are in close proximity (small L_P/σ), the protein concentrations at the BL are large enough to form a condensate. At intermediate distances ($L_P/\sigma = 2$), which corresponds to the BL and the RL just touching each other, the protein partitioning to the BL begins to decline sharply to a lower value. When the BL and the RL are far away ($L_P/\ \sigma >$ 2), the protein partitioning to the BL does not change much and stays at the same low value, which is not enough to form a condensate. This sharp decline in protein partitioning to the BL can be understood in the following way: when the lncRNA localized at the RL is close to the BL (i.e., small L_P/σ), it cooperatively helps recruit more protein to the BL due to protein-lncRNA attractive interactions and increases protein partitioning (Fig. S8). When the RL is far away from the BL, the lncRNA localized at the RL can attract protein to the RL due to attractive protein-lncRNA interactions. In this way, the RL competes with the BL to recruit proteins resulting in a sharp decline in protein partitioning to the BL (Fig. S8). In summary, we predict that lncRNAs have a local effect on protein partitioning and condensate formation. The RL transitions from *cooperating* with the BL to *competing* with the BL to recruit proteins beyond a distance of 2σ resulting in a sharp drop in protein recruitment to the BL.

The dynamics of this process reveals something interesting. Since the initial lncRNA concentration profile is peaked at the RL and decays with distance, the distance between the BL and the RL affects the initial lncRNA concentration at the BL, and therefore the dynamics of protein recruitment to the BL. To understand this effect, we graphed the concentration profiles of protein and lncRNA for three different values of the scaled distance L_P/σ. At small distances (Fig. 3 *C*, *left panel*), the RL and the BL are close enough that they almost overlap. The initial lncRNA concentrations at the BL are high because of their proximity to the RL. This helps start a positive feedback cycle, where high lncRNA concentrations at the BL help recruit more protein due to attractive protein-lncRNA interactions, which in turn recruits more lncRNA. This cycle continues until an equilibrium is reached. When the RL and the BL are quite far away (Fig. 3 *C*, *right panel*), the initial lncRNA concentration at the BL is quite low. In this case, only a small amount of lncRNA migrates from the RL to the BL. Since condensates form only beyond a threshold protein concentration (Fig. 2 *B*), the protein recruited to the BL due to this small amount of lncRNA may not be sufficient to help form a condensate despite the feedback cycle (Fig. 3 *C*). At intermediate distances (Fig. 3 *C*, *middle panel*), something interesting happens at equilibrium: the lncRNA concentration at the BL seems to be much higher than the RL even though initial lncRNA concentrations at the RL were higher. The time evolution of protein and lncRNA concentration profiles sheds light on this observation (Fig. 3 *D*). At intermediate times, we find that the lncRNA migrates from the RL to the BL. Once this happens, the lncRNA concentration at the BL increases and the positive feedback cycle is initiated, resulting in more protein recruitment.

To understand the mechanistic origin of lncRNA migration, we graphed the chemical potential profile of the lncRNA for different distances (Fig. 3 *E*). This profile dynamically evolves with time. As time progress, the protein accumulates at the BL because of the attractive well described by Eq. 2. Since proteins attract lncRNAs, increasing protein concentration at the BL makes it an attractive well for the lncRNA, which gets deeper with time as proteins accumulate the BL. At short distances (Fig. 3 *E*, *left panel*), the loci overlap and this well forms essentially at the same location as the RL. Therefore, there is an influx of lncRNA into this region that contains both the RL and the BL. When the distance between the loci is large (Fig. 3 *E*, *right panel*), not much protein accumulates at the BL initially due to low local lncRNA concentrations. This results in a shallower chemical potential well at the BL for the lncRNA with a chemical potential barrier between the BL and the RL at intermediate times, resulting in a lower migration of lncRNA to the BL. At intermediate distances, there is a partial overlap between the loci (Fig. 3 *E*, *middle panel*) and the chemical potential for the lncRNA at the BL starts decreasing with protein accumulation at the BL. This leads to a flux of lncRNA away from the RL and into the BL, which is what we see as lncRNA migration.

Given the contrasting effects of the two regulatory parameters—the amount of lncRNA (φ_R^{avg}) and normalized distance between loci (L_P/σ)—on protein recruitment to the BL, we wanted to understand their impact in conjunction (Fig. S6). In this figure, the contours correspond to combinations of lncRNA amount and distance that result in the same protein partitioning to the BL. We found that the effect of distance and lncRNA amounts can compensate for each other, resulting in the same value of protein partitioning to the BL for different combinations of these regulatory parameters.

Nonequilibrium effects can lead to both enhancement and repression of gene expression due to transcription of proximal lncRNAs

The transcription of neighboring lncRNAs can interfere with mRNA transcription by affecting protein concentrations and condensate formation at the BL. Therefore, we next wanted to understand how localized lncRNA transcription from the RL affects mRNA transcription from neighboring genes at the BL.

Supporting material, section 3, investigates the impact of just mRNA transcription in shaping condensate dynamics in the absence of lncRNA transcription (i.e., $k_R = 0$) to get some baseline expectations. We varied the mRNA

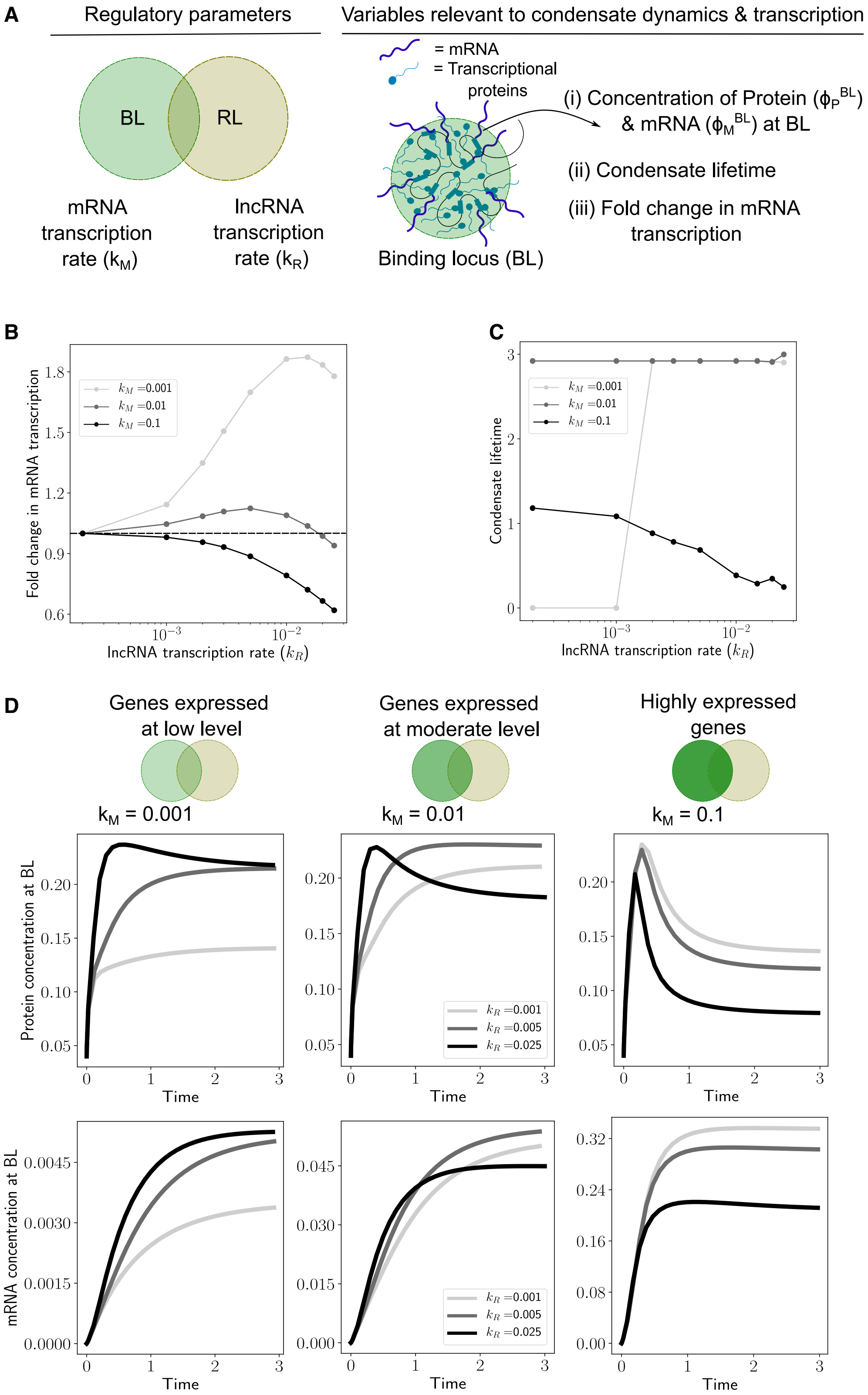

FIGURE 4 (*A*) In this figure, we change the transcription rate of the lncRNA (k_R) and study how that impacts condensate dynamics and mRNA transcription for three different regimes of gene expression—(*i*) genes expressed at low level ($k_M = 0.001$), (*ii*) genes expressed at a moderate level ($k_M = 0.01$), and (*iii*) highly expressed genes ($k_M = 0.1$). For each case, we quantified the fold change in mRNA transcription at steady state, condensate lifetime, and the dynamics of protein concentration (φ_P^{BL}) and mRNA concentration (φ_M^{BL}) at the BL. For all simulations results in this figure, the distance between the loci was

(legend continued on next page)

transcription rate constant k_M and studied the resultant phenomena. Simulations were done using low protein concentrations where the protein does not phase separate in the absence of mRNA transcription. As mRNA is transcribed at the BL, it attracts more protein to the BL, which in turn results in more mRNA transcription since the mRNA transcription rate is coupled to local protein concentration. For a gene expressed at a low level (low k_M), there is not enough mRNA transcription for this positive feedback cycle to recruit enough protein and form a condensate (Fig. S9 *B* and *C*). For moderately expressed genes (moderate k_M), there is enough transcription of mRNA and the positive feedback cycle results in a stable condensate at steady state (Fig. S9 *B* and *C*) with a long lifetime (Fig. S9 *D*). For highly expressed genes (large k_M), there is enough mRNA transcription to form a condensate (Fig. S9 *C*). But as mRNA accumulates, the entropic penalty of confining proteins and mRNAs into a dense phase reduces protein concentrations and results in a dissolved condensate at steady state (Fig. S9 *B*) with a short lifetime (Fig. S9 *D*). These results recapitulate the findings of previous related work in literature (29).

To study the impact of lncRNA transcription on mRNA transcription from the BL, we performed numerical simulations of the model described by Eqs. 6, 7, and 8. The lncRNA transcription rate k_R is an important regulatory parameter here. We increased the lncRNA transcription rate and quantified metrics related to condensate dynamics and gene expression for three different cases: genes expressed at low level, i.e., low k_M, genes expressed at moderate level, i.e., moderate k_M, and highly expressed genes, i.e., high k_M (Fig. 4 *A*).

For genes expressed at a low level (low k_M), we predict that active transcription of lncRNA at the RL enhances mRNA transcription (Fig. 4 *B*). In this regime, increasing the lncRNA transcription rate leads to an increase in mRNA transcription. This enhancement is accompanied by a corresponding sharp increase in condensate lifetime (Fig. 4 *C*), suggesting that proximal lncRNA transcription enhances protein recruitment to the BL through attractive interactions to form a condensate. This is consistent with the large increase in the protein concentration at the BL at steady state (Fig. 4 *D*, *top panel*) observed upon increasing the lncRNA transcription rate (k_R) from 0.001 to 0.005. Since the mRNA transcription rate is coupled to protein concentration (Eq. 7), this results in a higher rate of mRNA transcription and therefore higher gene expression, as measured by the steady-state concentration of mRNA (Fig. 4 *D*, *bottom panel*). However, there are limits to this enhancement in gene expression. Upon further increasing the lncRNA transcription rate k_R, the fold change in mRNA transcription reaches a peak and then reduces (Fig. 4 *B*, $k_M = 0.001$). This is a consequence of the reentrant effect of lncRNA concentration on protein condensation. The lncRNA concentration at the BL crosses over from a regime where lncRNA enhances protein recruitment to BL via attractive protein-RNA interactions, to a regime where the lncRNA hinders protein recruitment to the BL due to the entropic costs of confining the proteins and RNAs into a dense phase (Fig. 4 *B*). Transcription of proximal lncRNAs also speeds up response times for gene expression by increasing the initial rate of mRNA transcription (Fig. 4 *D*, *bottom panel*). The mRNA accumulates more quickly for higher values of k_R, and this is a nonequilibrium effect caused by active lncRNA transcription.

For genes expressed at a moderate level (moderate k_M), active transcription of lncRNA at the RL only has a mild effect on mRNA transcription (Fig. 4 *B*). In this regime, the condensate lifetime is predominantly determined by the dynamics of mRNA transcription and it does not change with increasing k_R (Fig. 4 *C*). The fold change in mRNA transcription has a nonmonotonic trend (Fig. 4 *B*). The dynamics of protein and mRNA concentrations at the BL sheds some light on this (Fig. 4 *D*, *middle panel*). The protein concentration at BL at steady state initially increases and then decreases with k_R. This is again a consequence of switching over to a regime where RNA-RNA repulsion and entropic costs of confining the RNAs and proteins dissolve the condensate. The dynamics (Fig. 4 *D*, *middle panel*) again reveals that transcription of proximal lncRNAs speeds up response times for gene expression.

For highly expressed genes (moderate k_M), active transcription of lncRNA at the RL has a largely repressive effect on gene expression as the fold change in mRNA transcription monotonically decreases with k_R (Fig. 4 *B*). In this regime, the high k_M already leads to condensate dissolution (Fig. 4 *D*, *right panel*). lncRNA transcription at the RL further destabilizes condensates as the condensate lifetime decreases with k_R (Fig. 4 *C*). Since increasing k_R reduces the protein concentration at the BL at steady state

$L_P = 0.8\sigma$ and the protein amount was $\varphi_P^{avg} = 0.04$. (*B*) Gene expression: fold change in mRNA transcription upon changing the lncRNA transcription rate for the three different gene expression regimes. The fold change in mRNA transcription is calculated as $= (\varphi_M^{BL}$ when lncRNA is being transcribed at rate $k_R)/(\varphi_M^{BL}$ when there is no lncRNA transcription, i.e., $k_R = 0)$. The dotted horizontal line corresponds to a fold change value of 1, which means that the lncRNA transcription neither enhances nor represses mRNA transcription. (*C*) Condensate lifetime: the dependence of condensate lifetime on lncRNA transcription rate for the three different regimes of gene expression. The condensate lifetime is also reported in the dimensionless units ($k_d t$), and is defined as the duration of time for which protein concentration at the BL is "appreciable." We chose a cutoff $\varphi_P^{BL} > 0.15$ to define appreciable protein concentration at the BL. Note that this specific numerical choice of the cutoff value does not change the qualitative nature of the trends or results. (*D*) Dynamics of condensate and gene expression: dynamics of protein and mRNA concentration at the BL. Each vertical panel corresponds to a different regime of gene expression. The top panel plots track the protein concentration at the BL with time upon increasing the lncRNA transcription rate (k_R). The bottom panel plots track the mRNA concentration at the BL with time. The time is reported in dimensionless units ($k_d t$), where k_d is the degradation rate of the mRNA.

(Fig. 4 *D*, *right panel*), this results in slower rates of mRNA transcription and therefore lower gene expression.

In summary, we find that lncRNA transcription has contrasting effects on mRNA transcription from genes expressed at a low level and highly expressed genes. Transcription of proximal lncRNAs increases transcription from the former and represses transcription from the latter. This follows directly from a nonequilibrium model where active lncRNA transcription affects condensate formation at the BL. lncRNA transcription in proximity can alter local RNA concentrations at the BL, which in turn has consequences for protein condensation, and therefore mRNA transcription.

DISCUSSION

In this study, we propose a simple physical mechanism by which lncRNAs can regulate transcriptional activation and transcription—via attractive interactions with transcriptional proteins that form condensates. Attractive interactions between transcriptional proteins and RNA could arise due to screened electrostatic attraction between oppositely charged polymers (29), which makes this a sequence-agnostic mechanism. At low RNA concentrations, these interactions promote condensation of proteins while high RNA concentrations lead to reentrant dissolution (Fig. 1 *B*). When coupled with equilibrium mechanisms (e.g., binding) or nonequilibrium mechanisms (e.g., spatially local transcription) that alter their local concentrations, lncRNAs can act as rheostats to fine-tune transcription from neighboring PCGs by regulating transcriptional condensates.

While there has been some experimental work investigating gene regulation by lncRNAs through transcriptional condensates (47), much remains to be understood. Our model makes specific predictions about how different regulatory parameters affect condensate formation, dynamics, and gene expression (Fig. 5), and it serves as a useful conceptual framework to understand many puzzling observations in the literature.

First, we predict that the presence of a proximal lncRNA near a BL such as a superenhancer, enhancer, or promoter can reduce threshold protein concentrations required for transcriptional condensate formation, enhance protein partitioning to these loci, and speed up the response time between a stimulus and transcriptional activation (Fig. 5 *A*).

Second, we predict that the lncRNAs have a spatially local effect on condensate formation (Fig. 5 *B*), which imposes physical constraints on the spatial and genomic organization of BLs and the lncRNAs that regulate them. This observation can provide a possible explanation for the origin of some known biological facts about lncRNAs. If lncRNAs function by recruiting transcriptional proteins to enhancers and promoters present locally, this can explain why many PCGs are preferentially surrounded by lncRNA-coding loci in their genomic neighborhood (4,10,11). Another puzzling fact about lncRNAs is that they have conserved synteny across vertebrates—their genomic positions relative to other genes are conserved rather than their sequence (16). If this local effect of lncRNAs is under evolutionary selection, the effect we predict imposes constraints on the spatial distance between lncRNA-coding genes and promoters. This, together with the observation that syntenic regions in mammals have evolutionarily conserved preferences for spatial contacts (48), can provide a mechanistic explanation for syntenic conservation of lncRNAs across vertebrates (16).

Finally, we predict that proximal transcription of lncRNAs represses gene expression from highly transcribed genes while enhancing gene expression from those expressed at a low level. This is also correlated with condensate stability—transcription of proximal lncRNAs enhances gene expression by stabilizing condensates and represses gene expression by destabilizing condensates, depending on the transcription rates of the lncRNA and the mRNA. Experiments that perturb lncRNA amounts and transcription and image condensates and measure gene expression can be used to test this model of whether lncRNAs regulate proximal BLs via interactions with components of transcriptional condensates. This observation also provides a useful framework to understand some conflicting findings in the literature. Studies of transcription regulation by lncRNAs show that they enhance transcription from neighboring PCGs in some cases and inhibit transcription in others (4,5). Fig. 5 *C* gives us a unifying principle that can help reconcile both these observations. For highly expressed genes, transcription of proximal lncRNAs predominantly has a repressive effect as the locally high mRNA concentrations at the BL disfavor condensate formation due to entropic penalties. For genes expressed at low levels, transcription of proximal lncRNAs predominantly enhances gene expression as lncRNAs help attract more protein to the BL via enthalpically favored interactions.

Rather than focus on a particular lncRNA or transcriptional protein, we use qualitative features of the nexus of interactions between proteins, RNA species, and chromatin such as the reentrant transition exhibited by RNA-protein phase diagrams (29), and the chromatin-assisted phase separation of transcriptional proteins (21) to understand their consequences for transcriptional condensates and transcription. Since our free energy expression captures phase diagrams of these interacting species qualitatively, our model parameters in principle can be inferred for specific systems by fitting to phase diagrams generated by either experiment (29), or through computational simulations such as those in (49). In this way, our model can be extended to work with experiments or simulations of specific RNA-protein systems.

In addition to the regulatory parameters studied in this paper, there is also emerging evidence that RNA secondary structure plays an important role in regulating the formation of biomolecular condensates (50). While we do not explicitly study this effect, our model could be extended to account for this. If we have a description of secondary

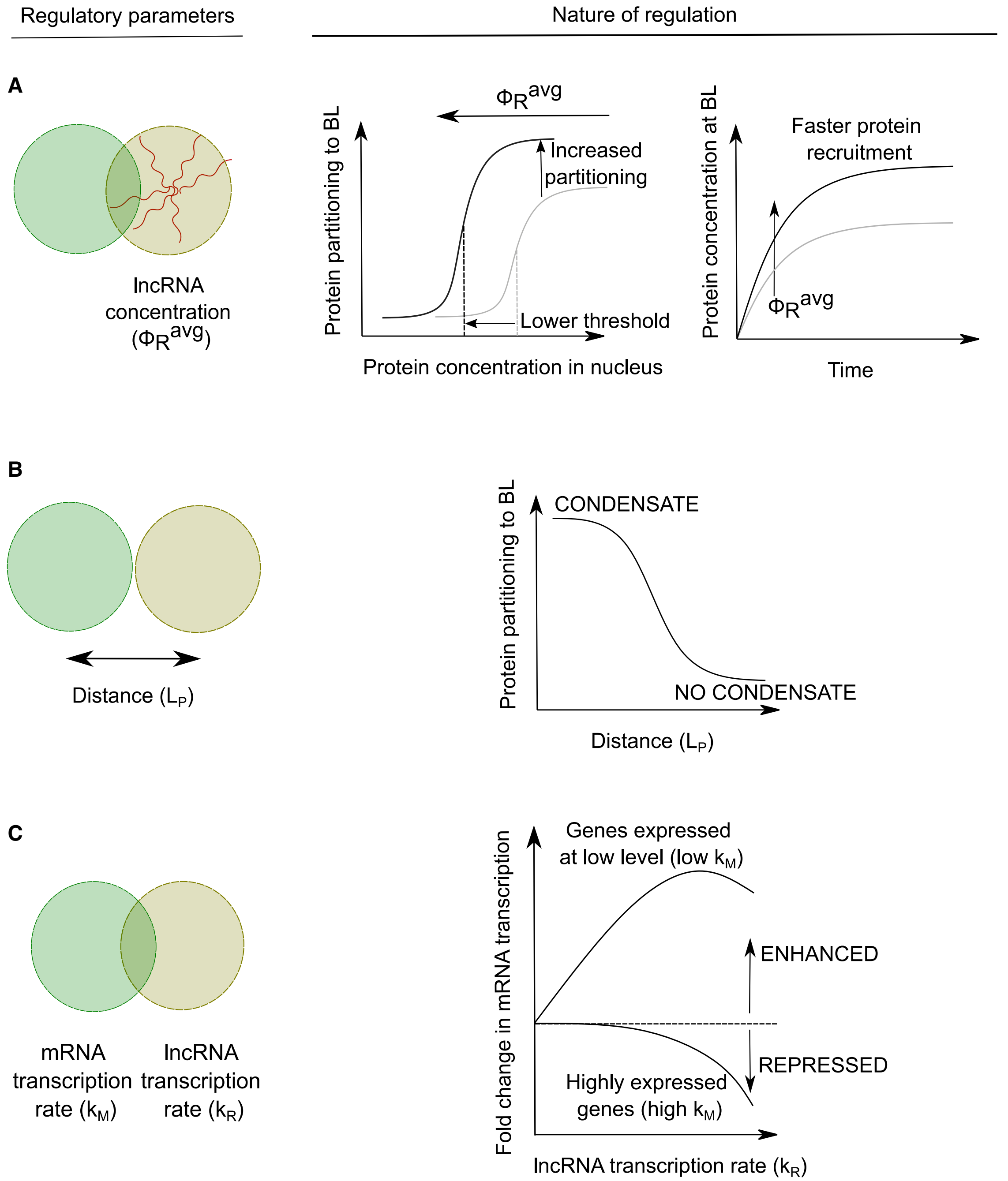

FIGURE 5 Proximal lncRNAs can regulate condensate formation and mRNA transcription in different ways depending on the regulatory parameter. (*A*) Increasing the concentration of proximal lncRNAs localized near a BL can bring down the concentration thresholds of transcriptional proteins required for condensate formation, enhancing the partitioning of these proteins into the condensate, and speed up protein recruitment to the BL. (*B*) lncRNAs exert a local effect in enhancing protein partitioning to the BL, and this effect sharply falls off with distance. This local effect can drive condensate formation, with the distance determining whether a condensate will form at the BL or not. (*C*) Transcription of proximal lncRNAs can increase mRNA transcription from genes expressed at low levels. For highly expressed genes, transcription of proximal lncRNAs represses gene expression.

structures of particular RNA species from experimental techniques such as SHAPE-MAP (51) and if we also know the specific transcriptional proteins this RNA interacts with, we could in principle perform molecular simulations to extract the RNA-protein interaction strength. This can then be an input into our model to perform simulations.

The model itself is agnostic to the identity of RNA species and the principles we identify in this study can be equally applied to understand gene regulation by other kinds of RNA species beyond lncRNAs. For example, this model can be used to understand how an actively transcribing mRNA can lead to transcriptional cross talk and affect the

transcription of neighboring mRNAs. Also, there are several RNA species that can be localized or transcribed near transcriptional condensates including lncRNAs, eRNAs, and divergently transcribed RNAs. These RNAs are often present in low copy numbers in cells (29,52). Even if the effect of a single locus is mild, several of these RNA loci can act cooperatively to regulate condensate formation and transcription. For example, it is well known that the chromatin is organized into topologically associating domains or TADs (53), which are characterized by a high contact probability of loci within the TAD. Therefore, lncRNAs could cooperatively regulate gene expression within the TAD. Investigating the nature of this cooperative regulation could be an interesting future direction of research.

Our model due to its simplified nature does have several limitations. First, our model is a mean-field description that ignores any stochastic effects that arise due to concentration fluctuations of the protein and RNA species. These fluctuations can be quite important for condensate nucleation and gene expression, and taking them into account can help make additional predictions about how lncRNAs can fine-tune the cell-cell heterogeneity of these phenotypes. Second, our model assumes that the protein concentrations follow model B dynamics based on a free energy that can be written in terms of the concentration fields. It is quite possible that the dynamics of transcriptional proteins within the dense milieu of condensates with many interacting species can be quite nontrivial and requires other model descriptions. Molecular simulations that model the dynamics of these interacting polymeric species will be required to test whether and when the approximations made in our simplified model break down. Finally, our model assumes that the RL and BL do not move much in transcription timescales, which are usually a few minutes for most RNAs. While this is a reasonable approximation given the low diffusivity of the chromatin loci (54), the dynamics of chromatin can couple with the dynamics of transcription and give rise to rich emergent physical phenomena that can provide insights into how transcription shapes genome organization and vice versa. This will be another interesting avenue for future research.

SUPPORTING MATERIAL

Supporting material can be found online at https://doi.org/10.1016/j.bpj.2023.05.032.

AUTHOR CONTRIBUTIONS

P.N. and A.K.C. conceived the project. P.N. and A.K.C. developed the model. K.S. provided initial code for simulations, which was further developed by P.N. P.N. ran numerical simulations, analyzed data, wrote the first draft of the manuscript, and designed the figures. K.S. provided helpful guidance throughout the project. All authors contributed to editing and revising the manuscript.

ACKNOWLEDGMENTS

P.N. and A.K.C. acknowledge support from NSF #MCB2044895. K.S. acknowledges support from the NSF–Simons Center for Mathematical and Statistical Analysis of Biology at Harvard (NSF #1764269) and the Harvard Faculty of Arts and Sciences Quantitative Biology Initiative. We thank Jonathan Henninger, Ozgur Oksuz, Kalon Overholt, Richard Young, and Phillip Sharp for several useful discussions.

DECLARATION OF INTERESTS

A.K.C. is a consultant (titled Academic Partner) for Flagship Pioneering and also serves as consultant and Strategic Oversight Board member of its affiliated company, Apriori Bio, and is a consultant and SAB member of another affiliated company, Metaphore, previously called FL77. He also owns equity in Dewpoint Therapeutics because he previously served on the SAB of this company.

SUPPORTING CITATIONS

References (55–63) appear in the supporting material.

REFERENCES

1. Zhao, L., H. Zheng, …, S. He. 2021. NONCODEV6: an updated database dedicated to long non-coding RNA annotation in both animals and plants. *J. Phys. Chem. A.* 125:165–174.
2. Uszczynska-Ratajczak, B., J. Lagarde, …, R. Johnson. 2018. Towards a complete map of the human long non-coding RNA transcriptome. *Nat. Rev. Genet.* 19:535–548.
3. Statello, L., C. J. Guo, …, M. Huarte. 2021. Gene regulation by long non-coding RNAs and its biological functions. *Nat. Rev. Mol. Cell Biol.* 22:96–118.
4. Luo, S., J. Y. Lu, …, X. Shen. 2016. Divergent lncRNAs regulate gene expression and lineage differentiation in pluripotent cells. *Cell Stem Cell.* 18:637–652.
5. Engreitz, J. M., J. E. Haines, …, E. S. Lander. 2016. Local regulation of gene expression by lncRNA promoters, transcription and splicing. *Nature.* 539:452–455.
6. Gil, N., and I. Ulitsky. 2020. Regulation of gene expression by cis-acting long non-coding RNAs. *Nat. Rev. Genet.* 21:102–117.
7. Fanucchi, S., E. T. Fok, …, M. M. Mhlanga. 2019. Immune genes are primed for robust transcription by proximal long noncoding RNAs located in nuclear compartments. *Nat. Genet.* 51:138–150.
8. Xiang, J. F., Q. F. Yin, …, L. L. Chen. 2014. Human colorectal cancer-specific CCAT1-L lncRNA regulates long-range chromatin interactions at the MYC locus. *Cell Res.* 24:513–531.
9. Lai, F., U. A. Orom, …, R. Shiekhattar. 2013. Activating RNAs associate with Mediator to enhance chromatin architecture and transcription. *Nature.* 494:497–501.
10. Ponjavic, J., P. L. Oliver, …, C. P. Ponting. 2009. Genomic and transcriptional co-localization of protein-coding and long non-coding RNA pairs in the developing brain. *PLoS Genet.* 5, e1000617.
11. Herriges, M. J., D. T. Swarr, …, E. E. Morrisey. 2014. Long noncoding RNAs are spatially correlated with transcription factors and regulate lung development. *Genes Dev.* 28:1363–1379.
12. Ørom, U. A., T. Derrien, …, R. Shiekhattar. 2010. Long noncoding RNAs with enhancer-like function in human cells. *Cell.* 143:46–58.
13. Anderson, K. M., D. M. Anderson, …, E. N. Olson. 2016. Transcription of the non-coding RNA UPPERHAND controls *Hand2* expression and heart development. *Nature.* 539:433–436.

14. Iyer, M. K., Y. S. Niknafs, …, A. M. Chinnaiyan. 2015. The landscape of long noncoding RNAs in the human transcriptome. *Nat. Genet.* 47:199–208.

15. Haerty, W., and C. P. Ponting. 2013. Mutations within lncRNAs are effectively selected against in fruitfly but not in human. *Genome Biol.* 14:R49-R16.

16. Hezroni, H., D. Koppstein, …, I. Ulitsky. 2015. Principles of long noncoding RNA evolution derived from direct comparison of transcriptomes in 17 species. *Cell Rep.* 11:1110–1122.

17. Ulitsky, I., A. Shkumatava, …, D. P. Bartel. 2011. Conserved function of lincRNAs in vertebrate embryonic development despite rapid sequence evolution. *Cell.* 147:1537–1550.

18. Quinodoz, S. A., J. W. Jachowicz, …, M. Guttman. 2021. RNA promotes the formation of spatial compartments in the nucleus. *Cell.* 184:5775–5790.e30.

19. Boija, A., I. A. Klein, …, R. A. Young. 2018. Transcription factors activate genes through the phase-separation capacity of their activation domains. *Cell.* 175:1842–1855.e16.

20. Sabari, B. R., A. Dall'Agnese, …, R. A. Young. 2018. Coactivator condensation at super-enhancers links phase separation and gene control. *Science.* 361, eaar3958.

21. Shrinivas, K., B. R. Sabari, …, A. K. Chakraborty. 2019. Enhancer features that drive formation of transcriptional condensates. *Mol. Cell.* 75:549–561.e7.

22. Cho, W. K., N. Jayanth, …, I. I. Cisse. 2016. RNA Polymerase II cluster dynamics predict mRNA output in living cells. *Elife.* 5:e13617–e13631.

23. Cho, W. K., J. H. Spille, …, I. I. Cisse. 2018. Mediator and RNA polymerase II clusters associate in transcription-dependent condensates. *Science.* 361:412–415.

24. Wei, M. T., Y. C. Chang, …, C. P. Brangwynne. 2020. Nucleated transcriptional condensates amplify gene expression. *Nat. Cell Biol.* 22:1187–1196.

25. Wu, J., B. Chen, Y. Lin…, 2022. Modulating gene regulation function by chemically controlled transcription factor clustering. *Nat. Commun.* 13:2663–2677.

26. Hnisz, D., K. Shrinivas, …, P. A. Sharp. 2017. A phase separation model for transcriptional control. *Cell.* 169:13–23.

27. Kim, T. K., M. Hemberg, …, M. E. Greenberg. 2010. Widespread transcription at neuronal activity-regulated enhancers. *Nature.* 465:182–187.

28. Li, W., D. Notani, and M. G. Rosenfeld. 2016. Enhancers as non-coding RNA transcription units: recent insights and future perspectives. *Nat. Rev. Genet.* 17:207–223.

29. Henninger, J. E., O. Oksuz, …, R. A. Young. 2021. RNA-mediated feedback control of transcriptional condensates. *Cell.* 184:207–225.e24.

30. Srivastava, S., and M. Tirrell. 2016. Polyelectrolyte complexation. *Adv. Chem. Phys.* 161:499–544.

31. Lin, Y. H., J. P. Brady, …, H. S. Chan. 2017. Charge pattern matching as a "fuzzy" mode of molecular recognition for the functional phase separations of intrinsically disordered proteins. *New J. Phys.* 19, 115003.

32. Lin, Y., J. McCarty, S. Han…, 2019. Narrow equilibrium window for complex coacervation of tau and RNA under cellular conditions. *Elife.* 8:e42571–e42601.

33. Banerjee, P. R., A. N. Milin, …, A. A. Deniz. 2017. Reentrant phase transition drives dynamic substructure formation in ribonucleoprotein droplets. *Angew Chem. Int. Ed. Engl.* 56:11354–11359.

34. Milin, A. N., and A. A. Deniz. 2018. Reentrant phase transitions and non-equilibrium dynamics in membraneless organelles. *Biochemistry.* 57:2470–2477.

35. Pak, C. W., M. Kosno, …, M. K. Rosen. 2016. Sequence determinants of intracellular phase separation by complex coacervation of a disordered protein. *Mol. Cell.* 63:72–85.

36. Nott, T. J., E. Petsalaki, …, A. J. Baldwin. 2015. Phase transition of a disordered nuage protein generates environmentally responsive membraneless organelles. *Mol. Cell.* 57:936–947.

37. Vernon, R. M., P. A. Chong, J. D. Forman-Kay…, 2018. Pi-Pi contacts are an overlooked protein feature relevant to phase separation. *Elife.* 7:e31486–e31533.

38. Wang, J., J. M. Choi, …, A. A. Hyman. 2018. A molecular grammar governing the driving forces for phase separation of prion-like RNA binding proteins. *Cell.* 174:688–699.e16.

39. Hou, L., Y. Wei, …, R. Jauch. 2020. Concurrent binding to DNA and RNA facilitates the pluripotency reprogramming activity of SO2. *Nucleic Acids Res.* 48:3869–3887.

40. Sigova, A. A., B. J. Abraham, …, R. A. Young. 2015. Transcription factor trapping by RNA in gene regulatory elements. *Science.* 350:978–981.

41. Jeon, Y., and J. T. Lee. 2011. YY1 tethers XIST RNA to the inactive X nucleation center. *Cell.* 146:119–133.

42. Werner, M. S., and A. J. Ruthenburg. 2015. Nuclear fractionation reveals thousands of chromatin-tethered noncoding RNAs adjacent to active genes. *Cell Rep.* 12:1089–1098.

43. Shi, K., T. Liu, …, X. Zheng. 2021. Genome-wide analysis of lncRNA stability in human. *PLoS Comput. Biol.* 17:e1008918–e1008925.

44. Chen, W., J. M. Smeekens, and R. Wu. 2016. Systematic study of the dynamics and half-lives of newly synthesized proteins in human cells. *Chem. Sci.* 7:1393–1400.

45. Hohenberg, P. C., and B. I. Halperin. 1977. Theory of dynamic critical phenomena. *Rev. Mod. Phys.* 49:435–479.

46. Guyer, J. E., D. Wheeler, and J. A. Warren. 2009. FiPy: partial differential equations with Python. *Comput. Sci. Eng.* 11:6–15.

47. Daneshvar, K., M. B. Ardehali, …, A. C. Mullen. 2020. lncRNA DIGIT and BRD3 protein form phase-separated condensates to regulate endoderm differentiation. *Nat. Cell Biol.* 22:1211–1222.

48. Imakaev, M., G. Fudenberg, …, L. A. Mirny. 2012. Iterative correction of Hi-C data reveals hallmarks of chromosome organization. *Nat. Methods.* 9:999–1003.

49. Regy, R. M., G. L. Dignon, …, J. Mittal. 2020. Sequence dependent phase separation of protein-polynucleotide mixtures elucidated using molecular simulations. *Nucleic Acids Res.* 48:12593–12603.

50. Langdon, E. M., Y. Qiu, …, A. S. Gladfelter. 2018. mRNA structure determines specificity of a polyQ-driven phase separation. *Science.* 360:922–927.

51. Langdon, E. M., and A. S. Gladfelter. 2018. Probing RNA structure in liquid–liquid phase separation using SHAPE-MaP. *Methods Enzymol.* 611:67–79.

52. Cabili, M. N., M. C. Dunagin, …, A. Raj. 2015. Localization and abundance analysis of human lncRNAs at single-cell and single-molecule resolution. *Genome Biol.* 16:20.

53. Nora, E. P., J. Dekker, and E. Heard. 2013. Segmental folding of chromosomes: a basis for structural and regulatory chromosomal neighborhoods? *Bioessays.* 35:818–828.

54. Gu, B., T. Swigut, …, J. Wysocka. 2018. Transcription-coupled changes in nuclear mobility of mammalian cis-regulatory elements. *Science.* 359:1050–1055.

55. Cai, Z., C. Cao, …, Y. Xue. 2020. RIC-seq for global in situ profiling of RNA–RNA spatial interactions. *Nature.* 582:432–437.

56. Beltran, B., D. Kannan, …, A. J. Spakowitz. 2019. Geometrical heterogeneity dominates thermal fluctuations in facilitating chromatin contacts. *Phys. Rev. Lett.* 123:208103–208104.

57. Valouev, A., S. M. Johnson, …, A. Sidow. 2011. Determinants of nucleosome organization in primary human cells. *Nature.* 474:516–520.

58. Derrien, T., R. Johnson, …, R. Guigó. 2012. The GENCODE v7 catalog of human long noncoding RNAs: analysis of their gene structure, evolution, and expression. *Genome Res.* 22:1775–1789.

59. Knauss, J. L., N. Miao, T. Sun…, 2018. Long noncoding RNA SOX2-ot and transcription factor YY1 co-regulate the differentiation of

cortical neural progenitors by repressing SOX2. *Cell Death Dis.* 9:799–811.

60. Schede, H. H., P. Natarajan, …, K. Shrinivas. 2022. Organization and regulation of nuclear condensates by gene activity. Preprint at bioRxiv. https://doi.org/10.1101/2022.09.19.508534.

61. Jung, C., P. Bandilla, …, U. Gaul. 2018. True equilibrium measurement of transcription factor-DNA binding affinities using automated polarization microscopy. *Nat. Commun.* 9:1605–1611.

62. Wu, L., P. Murat, …, S. Balasubramanian. 2013. Binding interactions between long noncoding RNA HOTAIR and PRC2 proteins. *Biochemistry.* 52:9519–9527.

63. Muskovic, W., E. Slavich, …, M. Kavallaris. 2022. High temporal resolution RNA-seq time course data reveals widespread synchronous activation between mammalian lncRNAs and neighboring protein-coding genes. *Genome Res.* 32:1463–1473.

Biophysical Journal, Volume *122*

Supplemental information

A model for *cis*-regulation of transcriptional condensates and gene expression by proximal lncRNAs

Pradeep Natarajan, Krishna Shrinivas, and Arup K. Chakraborty

A model for *cis*-regulation of transcriptional condensates and gene expression by proximal lncRNAs

Pradeep Natarajan[1], Krishna Shrinivas[2], and Arup K. Chakraborty[1,3,4,5,6,*]

[1]Department of Chemical Engineering, Massachusetts Institute of Technology, Cambridge MA 02139, USA
[2]NSF-Simons Center, Harvard University, Cambridge MA 02139, USA
[3]Department of Physics, Massachusetts Institute of Technology, Cambridge MA 02139, USA
[4]Institute of Medical Engineering and Science, Massachusetts Institute of Technology, Cambridge MA 02139, USA
[5]Ragon Institute of Massachusetts General Hospital, Massachusetts Institute of Technology and Harvard University, Cambridge MA 02139, USA
[6]Department of Chemistry, Massachusetts Institute of Technology, Cambridge MA 02139, USA
[*]Correspondence: arupc@mit.edu

Supporting Material

CONTENTS

S1 Typical distance between lncRNA loci (RL) and binding loci (BL) 1

S2 Model description 2
S2.1 Free-energy of interactions . . . 2
S2.1.1 Protein-Protein and Protein-BL interactions . . . 3
S2.1.2 Protein-RNA interactions . . . 4
S2.1.3 lncRNA-lncRNA and lncRNA-RL interactions . . . 6
S2.1.4 Parameters associated with the free energy . . . 7
S2.2 Dynamical equations . . . 7
S2.2.1 Dynamics of condensate formation . . . 7
S2.2.2 Dynamics of active transcription . . . 8
S2.2.3 Parameters associated with dynamics . . . 9
S2.3 Formulae to calculate different quantities to analyze simulation results . . . 9

S3 Condensate dynamics and mRNA transcription in the absence of actively transcribing lncRNAs 10

S4 Supplemental figures 11

S1 TYPICAL DISTANCE BETWEEN LNCRNA LOCI (RL) AND BINDING LOCI (BL)

lncRNA-coding loci are usually within a genomic distance of 100 kb from the promoter of protein-coding genes (1). Super-enhancers are typically located at a distance of around 250 kb from the promoters (2). Therefore, lncRNA-coding loci are usually located within a genomic distance of 350 kb of promoters, enhancers, and super-enhancers which we collectively call the binding loci (BL). To translate this genomic distance into a spatial distance, we use the worm-like chain model of chromatin described by Beltran et al. (3) that takes into account how nucleosome heterogeneity and different linker DNA lengths. The typical length of linker DNA in euchromatin of human cells is around 50 bp (4), and the Kuhn length of chromatin with this linker length is 37.8 nm (3).

Using the size of a nucleotide as 0.34 nm, the number of base pairs present in this Kuhn segment = 37.8 nm/0.34 nm × (146 bp in nucleosome + 50 bp in linker DNA)/50 bp linker DNA = 436 bp.

Therefore, a 1 kb separation along the genome corresponds to ≈ 2 Kuhn segments. Similarly, 350 kb corresponds to ≈ 800 Kuhn segments. Using a random polymer model as a crude approximation, end-end distance = (# of Kuhn segments)$^{1/2}$×Kuhn length, the average spatial distance between lncRNA-coding loci and the BL comes out to be ≈ 1 μm. Super-resolution microscopy studies reveal that transcriptional condensates that form at super-enhancers have a diameter in the range of 200-600 nm (5). Thus, the spatial distance between lncRNA-coding loci and promoters/super-enhancers is of the same order as the size of transcriptional condensates.

S2 MODEL DESCRIPTION

As mentioned in the paper, the model contains two components: the free energy of interactions between the species and the dynamical equations. We take a deeper look at the free-energy expression and its associated parameters in section S2.1. We take a deeper look at the dynamical equations and their associated parameters in section S2.2

S2.1 Free-energy of interactions

The free energy of interactions between the different species is given by the below expression:

$$F[\phi_P, \phi_R, \phi_M, \vec{r}] = F_{FH}[\phi_P, \phi_R, \phi_M] + F_{BL}[\phi_P, \vec{r}] + F_{RL}[\phi_R, \vec{r}] + F_{surf}[\phi_P] \tag{1}$$

- Here, $F_{FH}[\phi_P, \phi_R, \phi_M]$ is a Flory-Huggins free energy that captures the self and cross interactions between transcriptional proteins, lncRNA, and the mRNA.

$$F_{FH}[\phi_P, \phi_R, \phi_M] = \underbrace{\frac{\phi_P}{N_P}\log\phi_P + \frac{\phi_R}{N_R}\log\phi_R + \frac{\phi_M}{N_M}\log\phi_M + (1-\phi_P-\phi_R-\phi_M)\log(1-\phi_P-\phi_R-\phi_M)}_{\text{Entropy}}$$
$$- \underbrace{\chi_P\phi_P^2}_{\text{Protein-Protein}} - \underbrace{\chi_{PR}\phi_P(\phi_R+\phi_M)}_{\text{Protein-RNA}} + \underbrace{\chi_R(\phi_R+\phi_M)^2}_{\text{RNA-RNA}} \tag{2}$$

Here, ϕ_P, ϕ_R, and ϕ_M are the volume fractions of protein, lncRNA, and mRNA species in the solution. N_P, N_R, and N_M are the lengths of the protein, lncRNA, and mRNA species in units of solvent volume. The parameters χ_P, χ_R, and χ_{PR} correspond to the Flory-Huggins χ parameters that capture the mean-field pairwise interaction strength between protein-protein, RNA-RNA, and protein-RNA species respectively. We assume that the lncRNA and mRNA species interact with the same strength χ_{PR} with the proteins and χ_R with each other, as they have similar lengths and charges that are not too different (6). Since the volume fraction of the proteins can be converted to protein concentrations by a multiplicative scaling factor, we will be using volume fractions and concentrations as semantically equivalent while keeping this distinction in mind.

- The term $F_{BL}[\phi_P, \vec{r}]$ captures the interaction free energy of transcriptional proteins with the BL.

$$F_{BL}[\phi_P, \vec{r}] = -c_P e^{-\frac{|\vec{r}-\vec{r}_{BL}|^2}{\sigma_{BL}^2}}\phi_P \tag{3}$$

Here, c_P is a strength of interaction between the BL and transcriptional proteins, σ_{BL} represents the spatial extent of these interactions, $\vec{r}_{BL}$ is the position of the BL in space, and $\vec{r}$ is the position vector.

- The term $F_{RL}[\phi_R, \vec{r}]$ captures the interaction free energy of lncRNAs with the RL.

$$F_{RL}[\phi_R, \vec{r}] = -c_R e^{-\frac{|\vec{r}-\vec{r}_{RL}|^2}{\sigma_{RL}^2}}\phi_R \tag{4}$$

Here, c_R is a strength of interaction between the RL and lncRNAs, σ_{RL} represents the spatial extent of these interactions, and $\vec{r}_{RL}$ is the position of the RL in space.

- Finally, the term $F_{surf}[\phi_P] = \frac{\kappa}{2}|\phi_P|^2$ is a surface tension term that penalizes sharp gradients in protein concentration, with κ being the strength of this energy penalty.

We take a deeper look at the cooperative effects that can arise when these interactions are combined together in the subsequent sections. Specifically, section S2.1.1 looks at the cooperative effects of protein-protein and protein-BL interactions and maps out parameter regimes where the protein phase separates. The choice of parameters χ_P and c_P used in this study shows qualitatively similar behavior to experiments (Figure S2). The section S2.1.3 describes how the free energy $F_{RL}[\phi_R, \vec{r}]$ leads to the localization of lncRNA near the RL. Section S2.1.2 describes the RNA-Protein phase diagram that arises due to the Flory-Huggins interaction free energy between these species. The parameters χ_R and χ_{PR} used in this study qualitatively match the re-entrant phase diagram observed in experiments S5.

S2.1.1 Protein-Protein and Protein-BL interactions

Interactions between intrinsically disordered regions of transcriptional proteins can promote phase separation (7, 8). These protein-protein interactions that favor phase separation can be qualitatively captured using a mean-field free energy expression from the Flory-Huggins theory of polymer solutions:

$$F_{FH}[\phi_P] = \frac{\phi_P}{N_P}\log\phi_P + (1-\phi_P)\log(1-\phi_P) - \chi_P\phi_P^2 \tag{5}$$

Here, ϕ_P is the volume fraction of protein in the solution, N_P is the length of the protein in units of solvent volume, and χ_P is the Flory-Huggins interaction parameter that captures the magnitude of attractive interactions between protein molecules. Since the volume fraction of the proteins can be converted to protein concentrations by a multiplicative scaling factor, we will be using volume fractions and concentrations as semantically equivalent while keeping this distinction in mind.

For this study, we coarse-grained the proteins as having $N_P = 5$ beads. To study the phase separation of this protein, the interaction strength χ_P has to be large enough to support phase separation into two phases for some range of concentrations. According to the Flory-Huggins theory, for a polymer having N beads, $\chi > 0.5 + 1/\sqrt{N}$ results in phase separation into a dense and light phase. We chose a value of $\chi_P = 1.1$ (which is $> 0.5 + 1/\sqrt{5}$) for this study. The corresponding plot of chemical potential with the spinodal and binodal boundaries marked are depicted in figure S1A. Other than this, the particular numerical values of χ_P and N_P do not really affect the qualitative phase diagram. They only affect the concentration thresholds of the binodal and spinodal boundaries.

In addition to protein-protein interactions, there is also a surface tension associated with the protein molecules, and the protein molecules are attracted to BL through the free energy $F_{BL}[\phi_P, \vec{r}]$. The combined effect of surface tension, protein-protein, and protein-BL interactions is captured by the below free energy expression:

$$\begin{aligned} F_P[\phi_P, \vec{r}] = F_{FH}[\phi_P] + F_{BL}[\phi_P, \vec{r}] + F_{surf}[\phi_P] = \\ \phi_P\log\phi_P + (1-\phi_P)\log(1-\phi_P) - \chi_P\phi_P^2 - c_P e^{-|\vec{r}|^2/\sigma^2}\phi_P + \frac{\kappa}{2}|\nabla\phi_P|^2 \end{aligned} \tag{6}$$

In the above, we have set $\vec{r}_{BL} = 0$ meaning that the BL is at the origin. We set the value of the surface tension parameter to be small ($\kappa << 1$), enough to ensure that the condensate stays spherical while F_{FH} and F_{BL} dominate the free energy expression and dictate the phase diagrams. The chemical potential associated with this free energy is:

$$\mu_P[\phi_P, \vec{r}] = \frac{\delta\int F_P dV}{\delta\phi_P} = \frac{1+\log\phi_P}{N_P} - (1+\log(1-\phi_P)) - 2\chi_P\phi_p - c_P e^{-|\vec{r}|^2/\sigma^2} \tag{7}$$

Figure S1A plots this chemical potential as a function of ϕ_P for different values of distance r from the center of the region in space containing DNA binding sites. When the average protein concentration in the system ϕ_P is within the spinodal boundary, the free energy F_P is concave function of ϕ_P with $\frac{\partial^2 F_P}{\partial\phi_P^2} = \frac{\partial\mu_P}{\phi_P} < 0$. The system is unstable and its free energy can be minimized by splitting into a dense phase rich in protein and a light phase depleted in protein with their respective compositions ϕ_P given by the binodal boundary.

We obtained the equilibrium profiles $\phi_P(r)$ as a function of the radial position r in a circular domain by starting with a spatially uniform protein concentration and simulating the relaxation using Model B dynamics till steady-state (9):

$$\frac{\partial\phi_P(\vec{r},t)}{\partial t} = \vec{\nabla}.\left(D_P\phi_P\left(\vec{\nabla}\mu_P\right)\right) \tag{8}$$

The steady-state solution of the above equations is also the solution to the equation $\mu_p[\phi_P^{eq}, \vec{r}] =$ constant, which is the criterion for chemical equilibrium. The constant is specified if we fix the average protein concentration in the system to a fixed value i.e. $1/V\int\phi_P^{eq}(\vec{r})dV = \phi_P^{avg}$. Expanding out the equation:

$$\mu_P[\phi_P^{eq}, \vec{r}] = \frac{1+\log\phi_P^{eq}}{N_P} - (1+\log(1-\phi_P^{eq})) - 2\chi_P\phi_P^{eq} - c_P e^{-|\vec{r}|^2/\sigma^2} = \text{constant} \tag{9}$$

S1B and S1C depict the profiles $\phi_p(r)$ as a function of the radial position r for different values of ϕ_P^{avg} and c_P. Consider the system having average protein concentration ϕ_P^{avg} well outside the binodal region. The spatially varying free energy benefit $F_{BL}[\phi_P, \vec{r}]$ conferred by the protein-BL interactions leads to an accumulation of protein at the BL. Beyond a threshold value of ϕ_P^{avg} or c_P, the local protein concentration at the BL $\phi_P^{eq}(r=0)$ can cross the spinodal boundary and start forming a dense

phase of protein at $r = 0$. In figure S1C, we observe that a small increase in c_P from 0.05 to 0.1 results in a large increase in $\phi_P^{eq}(r = 0)$. In the same way, for every value of ϕ_P^{avg}, sufficiently strong protein-DNA interactions with $c_P > c_P^*(\phi_P^{avg})$ can result in the formation of a dense phase of protein at much lower protein concentrations (ϕ_P^{avg}) well below the spinodal boundary. $c_P^*(\phi_P^{avg})$ is defined graphically in figure S1A.

Figure S2A plots contours of the condensate area as we vary ϕ_P^{avg} and c_P in the simulations. We define a transcriptional condensate as a region in space having a protein concentration $\phi_P > 0.3$, which represents the center of the spinodal region. When we have a sufficient amount of protein ϕ_P^{avg} in the system or sufficiently strong magnitude c_P of the protein-DNA interactions, a condensate of non-zero area is nucleated. The white dotted line represents the curve $c_P = c_P^*(\phi_P^{avg})$. The simulations agree with our theoretical prediction that a condensate nucleates when $c_P > c_P^*(\phi_P^{avg})$. We note that the phase diagram in figure S2B is qualitatively similar to the experimentally measured phase diagrams S2B reported in (7).

We chose the protein concentration in the system $\phi_P^{avg} = 0.04$, which is well below the binodal boundary shown in figure S1A. This was chosen to be consistent with the biological observation that *in vivo* transcriptional protein concentrations are much lower than the binodal concentrations (7). Simulations for results in the main manuscript are done with a c_P value close to the critical boundary $c_P = c_P^*(\phi_P^{avg})$, to illustrate how the presence of other RNA species can alter the condensate formation process. Therefore, simulation results in the main manuscript are done for the parameters $\phi_P^{avg} = 0.04, c_p = c_P^*(\phi_P^{avg} = 0.04) \approx 0.2$ unless stated otherwise.

S2.1.2 Protein-RNA interactions

The disordered regions of many transcriptional proteins contain a net positive charge. They can attract negatively charged RNAs via screened electrostatic interactions (10). Prior studies have shown that the qualitative features of phase diagrams of charged polymers in solutions interacting via screened electrostatic interactions can be qualitatively captured via a mean-field Flory Huggins free energy expression (11). For a Protein-RNA solution, the free energy expression can be written down to be:

$$F_{FH}[\phi_P, \phi_R] = \frac{\phi_P}{N_P} \log \phi_P + \frac{\phi_R}{N_R} \log \phi_R + (1 - \phi_P - \phi_R) \log(1 - \phi_P - \phi_R) - \chi_P \phi_P^2 - \chi_{PR} \phi_P \phi_R + \chi_R \phi_R^2 \tag{10}$$

Here, χ_P captures the magnitude of protein-protein attractive interactions, χ_{PR} captures the RNA-protein attractive interactions and χ_R captures the magnitude of the screened RNA-RNA electrostatic repulsion. N_R is the coarse-grained length of the RNA species. The typical length of disordered regions of transcriptional proteins is not more than 1000 amino acids (10). On the other hand, lncRNAs and mRNAs have a length of the order of magnitude $\sim$ 10000 base pairs, around 10 times longer than proteins(6). Therefore, we set the length of the RNA polymers as $N_R = N_M = 50$ beads, 10 times the size of the protein polymer $N_P = 5$.

The solvent entropy term in equation 10 i.e. $(1 - \phi_P - \phi_R) \log(1 - \phi_P - \phi_R)$ can be rearranged as:

$$\begin{aligned}(1 - \phi_P - \phi_R) \log(1 - \phi_P - \phi_R) &= (1 - \phi_P - \phi_R)\Big[\log(1 - \phi_P) + \log\Big(1 - \frac{\phi_R}{1 - \phi_P}\Big)\Big] \\ &= (1 - \phi_P) \log(1 - \phi_P) + (1 - \phi_P) \log\Big(1 - \frac{\phi_R}{1 - \phi_P}\Big) - \phi_R \log(1 - \phi_P) - \phi_R \log\Big(1 - \frac{\phi_R}{1 - \phi_P}\Big)\end{aligned} \tag{11}$$

At dilute RNA and Protein concentrations $\phi_P << 1$ and $\phi_R << 1$, using the expansions $\log(1 - x) = -x - x^2/2 + ...$ and $1/(1 - x) = 1 + x + x^2 + ...$, the above terms can be expanded to yield the following expression for solvent entropy:

$$(1 - \phi_P - \phi_R) \log(1 - \phi_P - \phi_R) = (1 - \phi_P) \log(1 - \phi_P) + \phi_R \phi_P + \frac{\phi_R^2}{2} + \frac{\phi_R^2 \phi_P}{2} + \frac{\phi_R \phi_P^2}{2} + \frac{\phi_R^2 \phi_P^2}{2} + ... \tag{12}$$

Under this approximation, the free energy in equation 10 gets modified as:

$$\begin{aligned}F_{FH,mod}[\phi_P, \phi_R] = {} & \frac{\phi_P}{N_P} \log \phi_P + (1 - \phi_P) \log(1 - \phi_P) - \chi_P \phi_P^2 \\ & + \frac{\phi_R}{N_R} \log \phi_R + (1 - \chi_{PR}) \phi_P \phi_R + \Big(\chi_R + \frac{1}{2}\Big) \phi_R^2 + \frac{\phi_R^2 \phi_P + \phi_R \phi_P^2 + \phi_R^2 \phi_P^2}{2}\end{aligned} \tag{13}$$

The above free energy expression has nicer numerical properties and leads to lower numerical errors compared to the free energy expression 10 when simulating the dynamical equations in sections S2.2.1 and S2.2.2. Therefore, we will use this free energy expression for the rest of the study.

Before jumping to this, let us first establish that both F_{FH} and $F_{FH,mod}$ do not lead to any qualitative difference in the equilibrium RNA-protein phase diagram due to the approximations introduced. From the free energy expressions 10 and 13, we can generate the phase diagram by analyzing the Jacobian matrix of $F_{FH}[\phi_P, \phi_R]$ and $F_{FH,mod}[\phi_P, \phi_R]$ and with respect to the variables ϕ_P and ϕ_R:

$$J_{FH} = \begin{bmatrix} \frac{\partial^2 F_{FH}}{\partial \phi_P^2} & \frac{\partial^2 F_{FH}}{\partial \phi_P \partial \phi_R} \\ \frac{\partial^2 F_{FH}}{\partial \phi_P \partial \phi_R} & \frac{\partial^2 F_{FH}}{\partial \phi_R^2} \end{bmatrix} = \begin{bmatrix} \frac{1}{N_P \phi_P} + \frac{1}{1-\phi_P-\phi_R} - 2\chi_P & \frac{1}{1-\phi_P-\phi_R} - \chi_{PR} \\ \frac{1}{1-\phi_P-\phi_R} - \chi_{PR} & \frac{1}{N_R \phi_R} + \frac{1}{1-\phi_P-\phi_R} + 2\chi_R \end{bmatrix} \tag{14}$$

$$J_{FH,mod} = \begin{bmatrix} \frac{\partial^2 F_{FH,mod}}{\partial \phi_P^2} & \frac{\partial^2 F_{FH,mod}}{\partial \phi_P \partial \phi_R} \\ \frac{\partial^2 F_{FH,mod}}{\partial \phi_P \partial \phi_R} & \frac{\partial^2 F_{FH,mod}}{\partial \phi_R^2} \end{bmatrix} = \begin{bmatrix} \frac{1}{N_P \phi_P} + \frac{1}{1-\phi_P} - 2\chi_P + \phi_R + \phi_R^2 & 1 - \chi_{PR} + \phi_R + \phi_R + 2\phi_P\phi_R \\ 1 - \chi_{PR} + \phi_R + \phi_R + 2\phi_P\phi_R & \frac{1}{N_R \phi_R} + (2\chi_R + 1) + \phi_P + \phi_P^2 \end{bmatrix} \tag{15}$$

The system is unstable and can undergo phase separation in the regions in the $\phi_P - \phi_R$ space where the Jacobian has at least one negative eigenvalue, which corresponds to a region of concavity of the free energy. The brown regions in figure S4A and S4B correspond to this region of spinodal instability. We can see from these figures that the shapes of the spinodal regions are qualitatively similar for both the free energy expression F_{FH} and $F_{FH,mod}$.

When ϕ_P and ϕ_R are within this region, the system splits into two phases: a dense phase rich in RNA and protein and a light phase poor in both RNA and protein. The coexistence compositions $(\phi_P^{dense}, \phi_R^{dense})$ and $(\phi_P^{light}, \phi_R^{light})$ are obtained by solving the equations for equality of chemical potentials and osmotic pressures in the two phases, which form the criteria for multiphase equilibrium:

$$\mu_P(\phi_P^{dense}, \phi_R^{dense}) = \mu_P(\phi_P^{light}, \phi_R^{light}) \tag{16}$$

$$\mu_R(\phi_P^{dense}, \phi_R^{dense}) = \mu_R(\phi_P^{light}, \phi_R^{light}) \tag{17}$$

$$\Pi(\phi_P^{dense}, \phi_R^{dense}) = \Pi(\phi_P^{light}, \phi_R^{light}) \tag{18}$$

where the chemical potentials of protein and RNA are respectively $\mu_P = \frac{\partial F_{FH}}{\partial \phi_P}$, $\mu_R = \frac{\partial F_{FH}}{\partial \phi_R}$, and the osmotic pressure $\Pi = k_B T / V_{solution}\Big(F_{FH} - \mu_P \phi_P - \mu_R \phi_R\Big)$. In addition, the total amount of protein (ϕ_P) and RNA (ϕ_R) constrain the dense and light phase compositions in the following way:

$$\nu \phi_P^{dense} + (1 - \nu)\phi_P^{light} = \phi_P \tag{19}$$

$$\nu \phi_R^{dense} + (1 - \nu)\phi_R^{light} = \phi_R \tag{20}$$

The system of 5 equations 16- 20, need to be solved to get the variables ϕ_P^{dense}, ϕ_P^{light}, ϕ_R^{dense}, ϕ_R^{light}, and ν. Here, ν is the volume fraction of the dense phase.

We can obtain the full phase diagram upon varying ϕ_P and ϕ_R for the two different free energy expressions 10 and 13 using the procedure described above, which are shown in figures S4A and S4B respectively. The dotted lines in figures S4A and S4B show the tie-lines connecting coexistence concentrations between the dense and light phases of protein. The brown regions correspond to the regions of spinodal instability. We can see that the shapes of the spinodal boundaries and the tie-lines are qualitatively the same for both F_{FH} and $F_{FH,mod}$. Therefore, we will use $F_{FH,mod}$ for the rest of the study.

Experimentally measured phase diagrams of RNA with transcriptional proteins exhibits a re-entrant behavior, where the protein partitioning to the dense phase initially increases and then decreases upon increasing the RNA concentration as shown in figure S5B (10). To qualitatively capture this phenomenon, we require χ_{PR} to be attractive so that there is an increase in protein partitioning to the dense phase in the presence of RNA. A value of $\chi_{PR} = 1.2$ worked well for our model. We also require the RNA-RNA repulsion strength to be stronger than the RNA-protein attraction to result in the dissolution of the dense phase of protein at high RNA concentrations. We chose a value of $\chi_R = 2.0 > \chi_{PR} = 1.2$. For this choice of parameters i.e. $\chi_{PR} = 1.2, \chi_R = 2.0$ used in this study, the partition ratio $(\phi_P^{dense}/\phi_P^{light})$ calculated by solving equations 16-20 and varying RNA concentration ϕ_R (figure S5A) qualitatively matches very well with experimentally measured re-entrant phase diagrams for transcriptional coactivators with RNA species as shown in S5B.

We can also gain more insight from the full phase diagram in S4B. As we increase the RNA concentration ϕ_R in the system for a constant $\phi_P = 0.3$, we observe that the horizontal width of the coexistence curve initially increases and then decreases S4B. RNA promotes the partitioning of the protein into the dense phase at low ϕ_R by virtue of its attractive interactions with the protein. At high ϕ_R, the RNA-RNA repulsive interactions and the entropic penalty of excluding the solvent from the dense phase results make the partitioning of protein into a dense phase unfavorable. When the RNA concentrations are high, phase separation is completely suppressed. We can see that beyond a critical value of the RNA concentration i.e. $\phi_R > \phi_R^c = 0.17$, there is no phase separation and formation of a two-phase region (figure S4B).

S2.1.3 lncRNA-lncRNA and lncRNA-RL interactions

Recent studies show that long-non coding RNAs (lncRNAs) tend to be present in locally high concentrations near their coding loci (12), which we term in this study as the RL. Although the mechanisms that cause this localization is poorly understood, it could be a consequence of equilibrium effects such as tethering of lncRNAs to their DNA loci by proteins such as Polymerase II (13) and YY1 (14), or non-equilibrium effects such as localized production of lncRNAs coupled with diffusion (15).

In this section, we will model equilibrium mechanisms that keep lncRNAs bound to their DNA coding loci using similar arguments as section S2.1.1. The free energy of binding of lncRNAs to their RL as described before is:

$$F_{RL}(\phi_R, \vec{r}) = -c_R e^{-|\vec{r}|^2/\sigma^2} \phi_R \tag{21}$$

When we combine this with a Flory-Huggins expression for the lncRNA species that takes into account lncRNA-lncRNA repulsion and entropy of the lncRNA polymer and solvent, we get the below expression for the total free energy of the lncRNA-RL system:

$$F_R[\phi_R, \vec{r}] = \frac{\phi_R}{N_R} \log \phi_R + (1-\phi_R)\log(1-\phi_R) + \chi_R \phi_R^2 - c_R e^{-|\vec{r}|^2/\sigma^2} \phi_R \tag{22}$$

Here, N_R is the length of the RNA polymer, and χ_R is the magnitude of the repulsive strength between the RNA species, whose values are set to $N_R = 50$ and $\chi_R = 2.0$ as justified in the section S2.1.2.

Transcription factors bind to the DNA with a binding affinity in the 1-100 nM range (16) while the binding affinity of lncRNAs with chromatin binding proteins is slightly weaker, around 100-1000 nM (17). Assuming a similar density of binding sites for lncRNA and tethering proteins on the DNA, we expect c_R to be typically much lower than c_P. The strength of the lncRNA-RL interactions was set to a similar value as the protein i.e. $c_R = c_P = 0.2$ for this study, with the understanding that this probably represents an upper limit on the strength of the lncRNA-RL interactions.

The chemical potential associated with this free energy in equation 22:

$$\mu_R[\phi_R, \vec{r}] = \frac{\delta \int F_R dV}{\delta \phi_R} = \frac{1 + \log \phi_R}{N_R} - (1 + \log(1-\phi_R)) + 2\chi_R \phi_R - c_R e^{-|\vec{r}|^2/\sigma^2} \tag{23}$$

We obtained the equilibrium profiles $\phi_R(r)$ in a circular domain by starting with a spatially uniform RNA concentration ϕ_R^{avg} and simulating the Model B dynamics (9) until steady-state:

$$\frac{\partial \phi_R(\vec{r})}{\partial t} = \vec{\nabla}.\left(D_R \phi_R \left(\vec{\nabla} \mu_R\right)\right) \tag{24}$$

The steady-state solution of the above equations is also the solution to the equation $\mu_R[\phi_R^{eq}, \vec{r}] =$ constant, which is the criterion for chemical equilibrium. The constant is chosen in such a way that $1/V \int \phi_R^{eq}(\vec{r}) dV = \phi_R^{avg}$. Expanding out the equation:

$$\mu_R[\phi_R^{eq}, \vec{r}] = \frac{1 + \log \phi_R^{eq}}{N_R} - (1 + \log(1-\phi_R^{eq})) + 2\chi_R \phi_R^{eq} - c_R e^{-|\vec{r}|^2/\sigma^2} = \text{constant} \tag{25}$$

Figure S3A plots this chemical potential as a function of ϕ_R for different values of distance r from the center of the RL. Figure S3B depicts the equilibrium profiles $\phi_R(r)$ in for different values of ϕ_R^{avg}.

For low values of ϕ_R, the dominant ϕ_R-dependent term in the chemical potential is $\frac{\log \phi_R}{N_R}$, which comes from the entropy of RNA in solution. The equilibrium concentration profile is shaped by the balance between lncRNA-RL interactions that attract the lncRNA to the RL and thermal fluctuations that tend to equalize concentrations everywhere. In this regime, the equilibrium profile $\phi_R^{eq}(r)$ is obtained by solving the equation:

$$\mu_R[\phi_R^{eq}, \vec{r}] \approx \frac{1 + \log \phi_R^{eq}}{N_R} - c_R e^{-|\vec{r}|^2/\sigma^2} = \text{constant} \tag{26}$$

For intermediate values of ϕ_R, the dominant ϕ_R-dependent term in the chemical potential is $2\chi_R\phi_R$, which comes from the lncRNA-lncRNA repulsive interactions. The equilibrium concentration profile is shaped by the balance between lncRNA-RL interactions that attract the lncRNA to the RL and RNA-RNA repulsions that tend to equalize concentrations everywhere. In this regime, the equilibrium profile $\phi_R^{eq}(r)$ is obtained by solving the equation:

$$\mu_R[\phi_R^{eq}, \vec{r}] \approx 2\chi_R\phi_R^{eq} - c_R e^{-|\vec{r}|^2/\sigma^2} = \text{constant} \tag{27}$$

In this regime, the free energy penalty imposed by lncRNA-lncRNA repulsion linearly scales with ϕ_R. Therefore, the RL gets saturated with a fixed amount of lncRNA and any additional lncRNA added will get uniformly distributed across the system. This is the reason that the profiles $\phi_R^{eq}(r)$ in figure S3B for intermediate values of $\phi_R^{avg} = 0.005$ and $\phi_R^{avg} = 0.01$ maintain their Gaussian shape while just being shifted up by some constant amount.

S2.1.4 Parameters associated with the free energy

Parameter	Value	Description	Rationalization
N_P	5.0	Length of coarse-grained protein sequence	Section S2.1.1
χ_P	1.1	Protein-protein attraction strength	Section S2.1.1
c_P	0.2	Protein-BL interaction strength	Section S2.1.1
ϕ_P^c	0.3	Protein concentration threshold to determine dense phase/condensate	Figure S4B
N_R	50.0	Length of coarse-grained lncRNA sequence	Section S2.1.3
N_M	50.0	Length of coarse-grained mRNA sequence	Section S2.1.3
χ_R	2.0	RNA-RNA repulsion strength	Section S2.1.2
χ_{PR}	1.2	RNA-protein interaction strength	Section S2.1.2
c_R	0.2	lncRNA-RL interaction strength	Section S2.1.3

Table S1: Table of parameters associated with the free energy expression

S2.2 Dynamical equations

In this section, we will look at how the dynamical equations for the concentration fields of the RNA and protein species are written for the two different cases studied in the paper: (i) condensate formation and (ii) active transcription

S2.2.1 Dynamics of condensate formation

The dynamics of transcriptional condensates happen over time scales of minutes (18). At these time scales, RNAs are not being turned over (19) and the total amount of RNAs in the system can be considered a conserved parameter. The same applies to proteins which are also quite stable over time scales of minutes. Taking into account all the relevant interactions between the proteins, lncRNAs, BL, and RL as described in section S2.1 the overall free energy of this system is:

$$F[\phi_P, \phi_R] = \underbrace{\frac{\phi_P}{N_P}\log\phi_P + \frac{\phi_R}{N_R}\log\phi_R + (1-\phi_P-\phi_R)\log(1-\phi_P-\phi_R)}_{\text{Entropy}} - \underbrace{\chi_P\phi_P^2}_{\text{Protein-Protein}} - \underbrace{\chi_{PR}\phi_P\phi_R}_{\text{Protein-RNA}} + \underbrace{\chi_R\phi_R^2}_{\text{RNA-RNA}}$$
$$- \underbrace{c_P e^{-|\vec{r}-\vec{r}_{BL}|^2/\sigma^2}\phi_P}_{\text{Protein-chromatin}} - \underbrace{c_R e^{-|\vec{r}-\vec{r}_{RL}|^2/\sigma^2}\phi_R}_{\text{RNA-DNA}} + \underbrace{\frac{\kappa}{2}|\nabla\phi_P|^2}_{\text{Surface Tension}} \tag{28}$$

Using the approximation for the solvent entropy (equation 12), the free energy can be rewritten as:

$$F[\phi_P, \phi_R] = \frac{\phi_P}{N_P}\log\phi_P + (1-\phi_P)\log(1-\phi_P) - \chi_P\phi_P^2 + \frac{\phi_R}{N_R}\log\phi_R + (1-\chi_{PR})\phi_P\phi_R + \left(\chi_R + \frac{1}{2}\right)\phi_R^2 + \frac{\phi_R^2\phi_P + \phi_R\phi_P^2 + \phi_R^2\phi_P^2}{2} - c_P e^{-|\vec{r}-\vec{r}_{BL}|^2/\sigma^2}\phi_P - c_R e^{-|\vec{r}-\vec{r}_{RL}|^2/\sigma^2}\phi_R + \frac{\kappa}{2}|\nabla\phi_P|^2 \tag{29}$$

Initially, the lncRNA species are localized at the RL with a concentration profile $\phi_R(\vec{r}, t=0) = \phi_R^{eq}(\vec{r})$, where $\phi_R^{eq}(\vec{r})$ is described in section S2.1.3. The protein concentrations are uniform throughout the domain i.e. $\phi_P(\vec{r}, t=0) = \phi_P^{avg} = constant$. The concentration profiles of the proteins and lncRNA relax to a new equilibrium. The coupled dynamics of relaxation to equilibrium can be captured by simulating the following Model B equations (9) until steady-state:

$$\frac{\partial\phi_P(\vec{r})}{\partial t} = \vec{\nabla}.\left(D_P\phi_P\left(\vec{\nabla}\mu_P\right)\right) \tag{30}$$

$$\frac{\partial\phi_R(\vec{r})}{\partial t} = \vec{\nabla}.\left(D_R\phi_R\left(\vec{\nabla}\mu_R\right)\right) \tag{31}$$

Where $\mu_P = \frac{\delta\int FdV}{\delta\phi_P}$ and $\mu_R = \frac{\delta\int FdV}{\delta\phi_R}$. The steady state of these equations is the new equilibrium profiles of the protein and lncRNA species, which can also be obtained by solving $\mu_P[\phi_P^{eq}, \phi_R^{eq}, \vec{r}] = \text{constant}$ and $\mu_R[\phi_P^{eq}, \phi_R^{eq}, \vec{r}] = \text{constant}$. The data in figures 2 and 3 of the main text are generated using these equilibrium protein and lncRNA concentration profiles, except for the plots of the dynamics.

S2.2.2 Dynamics of active transcription

Active transcription and depletion of RNAs can change the RNA concentrations and provide a driving force that pushes the system out of equilibrium. In our model, we make a distinction between two kinds of RNAs - (i) mRNAs which are transcribed from BLs such as promoters of protein-coding genes. The production rates of mRNAs are coupled to the local protein concentrations ϕ_P and (ii) lncRNAS, which are transcribed from nearby DNA present in the vicinity of BL. The production rates of these RNAs in general do not depend on the concentration of transcriptional proteins and their transcription rate is independent of ϕ_P. This generality can be broken if there is reason to believe that the same transcriptional proteins regulate the transcription of both the mRNAs and lncRNA species for specific systems.

To first understand the effect of localized mRNA transcription on the dynamics of transcriptional condensates, we model the dynamics of $\phi_P(\vec{r}, t)$ using Model B dynamics (9) and couple this to a reaction-diffusion model for the dynamics of the concentration field that corresponds to mRNA (ϕ_M):

$$\frac{\partial\phi_P(\vec{r}, t)}{\partial t} = \vec{\nabla}.\left(D_P\phi_P\left(\vec{\nabla}\mu_P\right)\right) \tag{32}$$

$$\frac{\partial\phi_M(\vec{r}, t)}{\partial t} = D_M\nabla^2\phi_M + \underbrace{k_M e^{\frac{-|\vec{r}-\vec{r}_{BL}|^2}{\sigma^2}}}_{k_M(\vec{r})}\phi_P - k_d\phi_M \tag{33}$$

The mRNA transcription rate constant $k_M(\vec{r}) = k_M e^{\frac{-|\vec{r}-\vec{r}_{BL}|^2}{\sigma^2}}$ is assumed to be a Gaussian function, peaked around the BL, motivated by the fact that mRNA transcription starts at the promoter of the protein-coding gene and decays as we progressively move away in space. A Gaussian function models this phenomenon well and captures the relevant biology (15).

From the above equations: (i) the dynamics of the field $\phi_P(\vec{r}, t)$ is coupled to the dynamics of the field $\phi_M(\vec{r}, t)$ via the protein-RNA interactions captured by the free energy F (ii) the dynamics of the field $\phi_M(\vec{r}, t)$ is coupled to the dynamics of the field $\phi_P(\vec{r}, t)$ as the rate of production of the RNA depends on $\phi_P(\vec{r}, t)$. These couplings result in RNA production acting as feedback on the protein transport, resulting in different non-equilibrium steady states depending on the system parameters. We investigate the consequences of these equations in detail in section S3.

Next, we would like to understand how the transcriptional dynamics of lncRNAs produced near transcriptional condensates interferes and affects the dynamics of $\phi_P(\vec{r}, t)$. To study this, we compare the dynamics described by equations 32- 33 with the

below equations in the presence of the second RNA species i.e. lncRNAs, by progressively increasing the lncRNA transcription rate k_M:

$$\frac{\partial \phi_P(\vec{r},t)}{\partial t} = \vec{\nabla}.\left(D_P \phi_P\left(\vec{\nabla}\frac{\delta F}{\delta \phi_P}\right)\right) \tag{34}$$

$$\frac{\partial \phi_M(\vec{r},t)}{\partial t} = D_M \nabla^2 \phi_M + \underbrace{k_M e^{\frac{-|\vec{r}-\vec{r}_{BL}|^2}{\sigma^2}}}_{k_M(\vec{r})} \phi_P - k_d \phi_M \tag{35}$$

$$\frac{\partial \phi_R(\vec{r},t)}{\partial t} = D_R \nabla^2 \phi_R + \underbrace{k_R e^{\frac{-|\vec{r}-\vec{r}_{RL}|^2}{\sigma^2}}}_{k_R(\vec{r})} - k_d \phi_R \tag{36}$$

Here, the rate constants $k_M(\vec{r})$ and $k_R(\vec{r})$ are modeled as spatially dependent Gaussians centered around the BL and the RL. The peak values of these Gaussians are k_M and k_R and their widths are both fixed to be the same value σ for simplicity.

In the above model, the transcription of the proximal lncRNAs can perturb the dynamics of the base system described by equations 32- 33. The extent of this perturbation will depend on the parameter k_R.

S2.2.3 Parameters associated with dynamics

In the model equations D_P, D_R and D_M are the diffusivities of the transcriptional proteins, lncRNAs, and mRNAs respectively in a dilute solution. For lncRNAs and mRNAs that are being actively transcribed, we assume that they are strongly tethered to the chromatin by RNA Polymerase II, and their diffusivity is the same as the diffusivity of the BL or the RL (10). Diffusivity of actively transcribed chromatin loci are of the order $10^{-3} - 10^{-2}\mu m^2/s$ (20) which is about 10-1000 times smaller than the diffusivity of transcriptional proteins which are of the order of $0.1 - 1\mu m^2/s$ (21). Therefore, we set $D_P = 100$ and $D_R = D_M = 0.1$ for our simulations.

k_{dR} and k_{dM} are the first-order degradation rates of the lncRNA and the protein respectively. The half-lives of RNAs span a range of time scales from minutes to hours. However, the median half-lives are not that different and are of the same order of magnitude for both mRNAs and lncRNAs – both being a few hours (19). Therefore, we set both degradation rate constants to the same value i.e. $k_{dM} = k_{dR}$. We chose a value of $k_{dM} = k_{dR} = k_d = 0.02$ for our simulations. This value was chosen such that the half-life of the RNA species $\ln 2/k_d \approx 35$ is an order of magnitude larger than the protein diffusion time scale $\tau_D = r^2/D_P = 2.25$. This is consistent with biological reality where transcriptional proteins diffuse much faster (21) than RNA half-lives (19).

The parameters k_M and k_R quantify the magnitude of mRNA and lncRNA transcription rates and σ_M and σ_R refer to the spatial extent of these molecules. Since the mean lengths of lncRNAs and mRNAs in the human genome are of the same order of magnitude (22) – around 10 kb, we set $\sigma_M = \sigma_R = \sigma = 5$.

Parameter	Value	Description
D_P	100	Protein diffusivity
D_R	0.1	lncRNA diffusivity
D_M	0.1	mRNA diffusivity
σ_R	5	Spatial extent of lncRNA locus
σ_M	5	Spatial extent of mRNA locus
k_{dR}	0.02	lncRNA degradation rate
k_{dM}	0.02	mRNA degradation rate
‘		

Table S2: Table of parameters associated with dynamical equations

S2.3 Formulae to calculate different quantities to analyze simulation results

In the below expressions, the species s could refer to transcriptional proteins, lncRNA, or mRNA.

- Concentration of a species s at the BL = $\phi_s^{BL} = \frac{\int_{|\vec{r}-\vec{r}_{BL}|<\sigma} \phi_s(\vec{r},t)dV}{\int_{|\vec{r}-\vec{r}_{BL}|<\sigma} dV}$

- Concentration of a species s outside the BL = $\phi_s^{out} = \frac{\int_{|\vec{r}-\vec{r}_{BL}|>\sigma} \phi_s(\vec{r},t) dV}{\int_{|\vec{r}-\vec{r}_{BL}|>\sigma} dV}$
- Average concentration of a species s in the system = $\phi_s^{avg} = \frac{\int \phi_s(\vec{r},t) dV}{\int dV}$
- Protein partitioning to the BL = $\frac{\phi_s^{BL}}{\phi_s^{out}}$
- Chemical potential of species s = $\frac{\delta \int F dV}{\delta \phi_s}$

S3 CONDENSATE DYNAMICS AND MRNA TRANSCRIPTION IN THE ABSENCE OF ACTIVELY TRANSCRIBING LNCRNAS

To get some baseline expectations, we first study the effect of actively transcribing mRNA on the condensate dynamics, in the absence of any active transcription of lncRNAs. We vary the transcription rate constant k_M of mRNAs and map out the nature of the non-equilibrium steady state and the dynamics of approach. This is described in figure S9A.

As we increase the transcription rate constant k_M, the mRNA concentration at the BL locus at steady state increases (Figure S9B). The amount of protein at the BL locus however initially increases and then decreases (Figure S9B). This is consistent with our expectations from the re-entrant phase diagram described in section S2.1.2. At low k_M, the active transcription of mRNA which depends on the local protein concentration $\phi_P(\vec{r})$ couples with the mRNA-protein interactions to result in a positive feedback loop that helps recruit more protein to the BL. At high k_M, a lot of mRNA is produced in the system at steady state and there is a region in space for which it is unfavorable to form a 2-phase system. This corresponds to the case where enough mRNA is produced to locally dissolve the dense phase of protein due to re-entrant transition. From figures S9B and S9C, we can see that the protein recruitment to the BL locus goes down for high $k_M \geq 0.1$ and the dense phase of protein dissolves. It dissolves from the inside-out and the protein in the system accumulates at the periphery of the BL with most of the RNA being present in the center.

The dynamics of protein recruitment to the BL locus again has two regimes (Figure S9C, Figure S9D). At low k_M, increasing k_M increases the recruitment of protein to the BL at steady state and results in the formation of a stable dense phase of protein (Figure S9D). At high k_M, the mRNA concentrations at the BL can cross $\phi_R^{BL} = \phi_R^c = 0.17$, which locally dissolves the dense phase of protein and results in a short-lived condensate (Figure S9D). This is consistent with prior experimental results relating the amount of mRNA transcribed and condensate lifetimes (23).

S4 SUPPLEMENTAL FIGURES

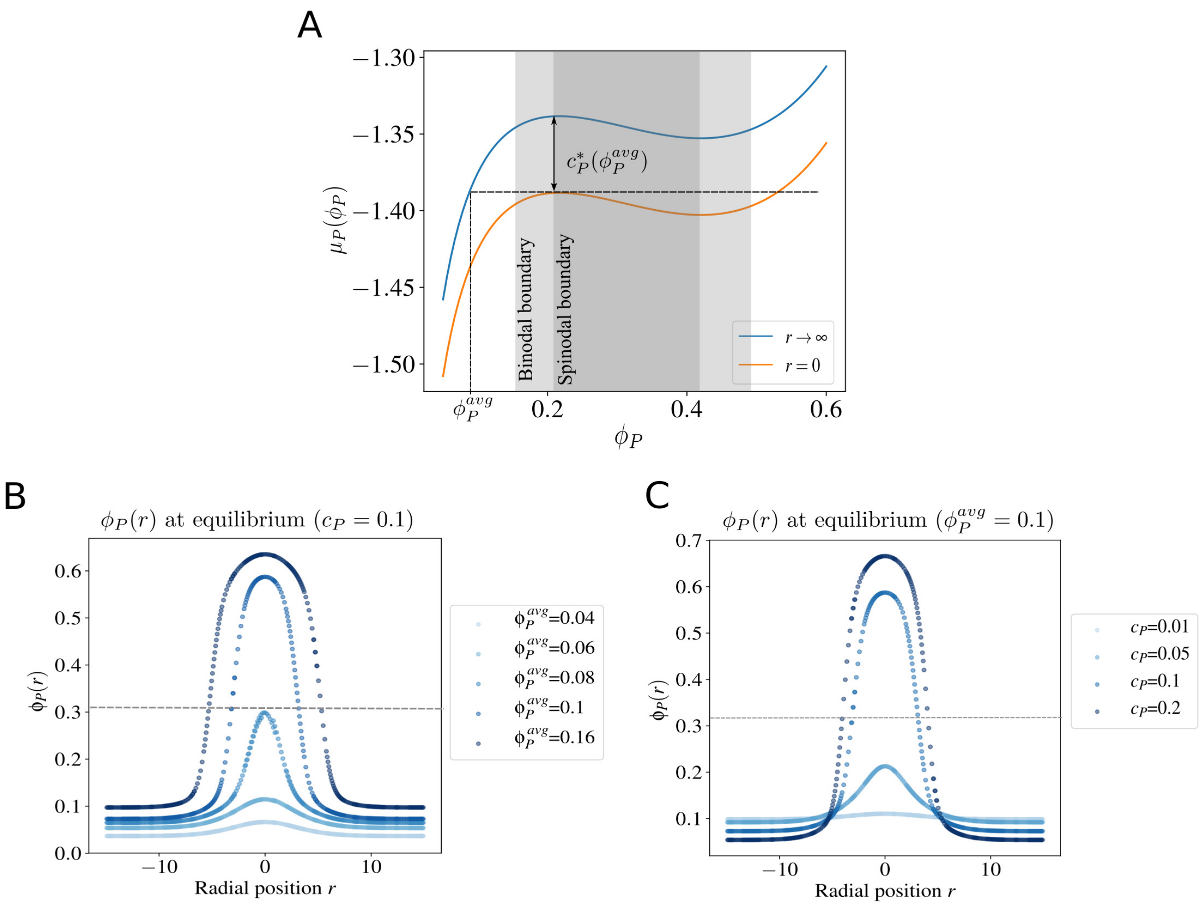

Figure S1: (A) the protein chemical potential μ_P as a function of the protein concentration ϕ_P for $\chi_P = 1.1$, $N_P = 5$ at $r = 0$ and $r \longrightarrow \infty$. The edges of the light gray region represent the values of ϕ_P that correspond to the coexistence concentrations of proteins in the light and dense phase. The dark gray region represents the region of spinodal instability. For a given amount of protein in the system as quantified by ϕ_P^{avg}, $c_P^*(\phi_P^{avg})$ is the depth of the Gaussian chemical potential well required to locally recruit enough protein at the BL at $r = 0$ to cross the spinodal boundary and form a dense phase of protein (B) Protein concentration profile $\phi_P(r)$ along the radial direction for different amounts of protein in the system ϕ_P^{avg} at constant $c_P = 0.1$, obtained by numerically integrating equation 8 till steady state (C) Protein concentration profile of $\phi_P(r)$ along the radial direction at constant $\phi_P^{avg} = 0.1$ for different depths of the chemical potential well c_P, obtained by numerically integrating equation 8 till steady state.

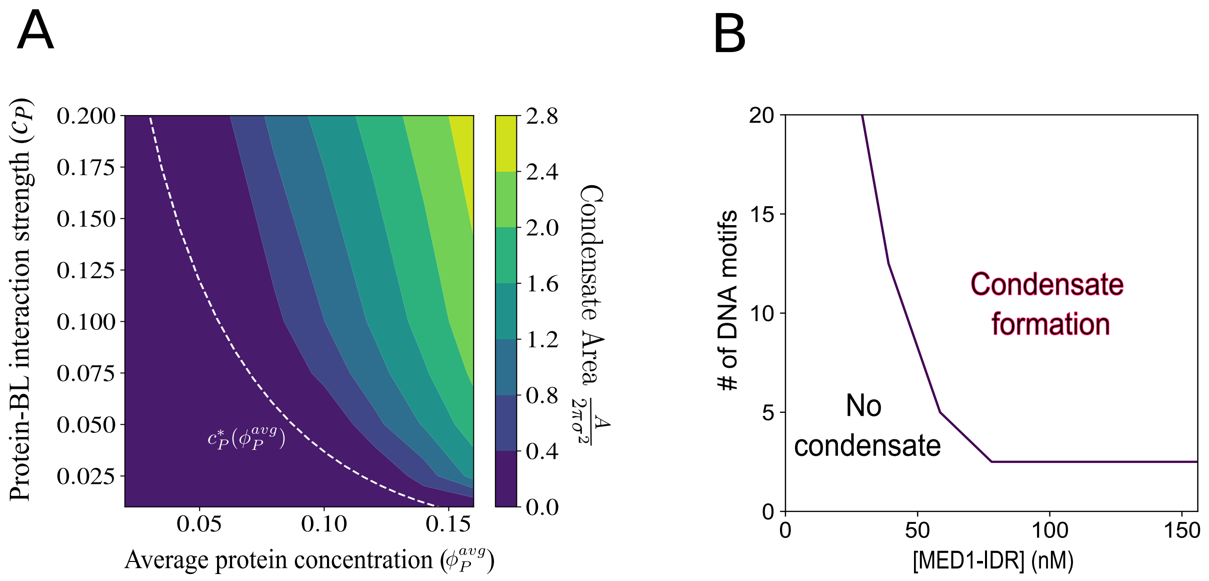

Figure S2: (A) Area of the transcriptional condensate $A/\pi\sigma^2$ at steady state of equation 8 for different amounts of protein in the system (ϕ_P^{avg}) and the strength of protein-BL interaction (c_P). Condensate area A is defined as the area of the region in space where the protein concentration $\phi_P(r) > 0.3$. The free energy parameters used for this plot are $N_P = 5$ and $\chi_P = 1.1$. We can see that the protein does not phase separate for concentrations $\phi_P^{avg} < 0.15$ as these concentrations are below the binodal boundary shown in figure S1A. Increasing c_P promotes condensate formation, resulting in condensate formation for protein concentrations below the binodal boundary. (B) The experimentally measured phase diagram upon varying the number of *Oct4*-DNA motifs and concentration of the transcriptional coactivator Mediator subunit 1 (MED1-IDR) from (7), Figure 4C. The parameters used in this study qualitatively match the experimentally measured phase diagram. MED1-IDR interacts with *Oct4* proteins to form a condensate at regions of chromatin containing *Oct4*-DNA motifs. The concentration of *Oct4* proteins is kept constant for this experiment. Therefore, increasing the number of DNA motifs leads to increased MED1-IDR interaction with the DNA

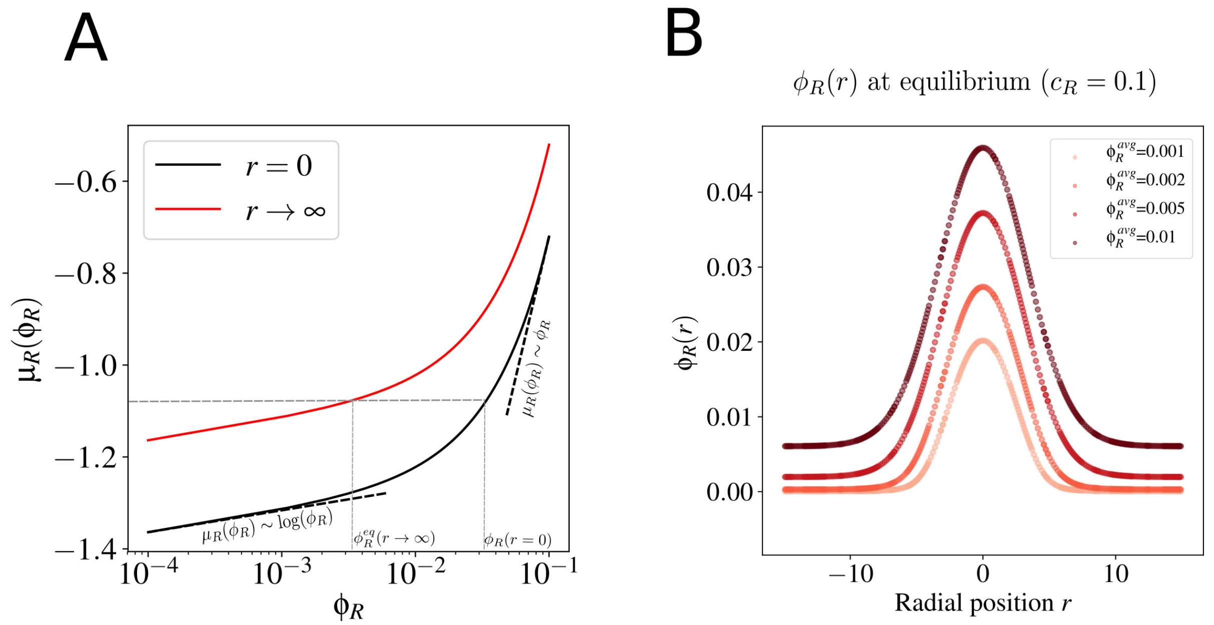

Figure S3: (A) The RNA chemical potential μ_R as a function of the RNA concentration ϕ_R for $c_R = 0.2$, $N_R = 50$, and $\chi_R = 2.0$ at $r = 0$ and $r \to \infty$. The RNA profile at equilibrium $\phi_R^{eq}(r)$ lies within the range $\phi_R^{eq}(r = 0)$ and $\phi_R^{eq}(r \to \infty)$, peaking at $r = 0$ (B) The equilibrium RNA concentration profile $\phi_R^{eq}(r)$ for different average RNA concentrations in the system ϕ_R^{avg} (at fixed $c_R = 0.1$), obtained by numerically integrating equation 24 till steady state

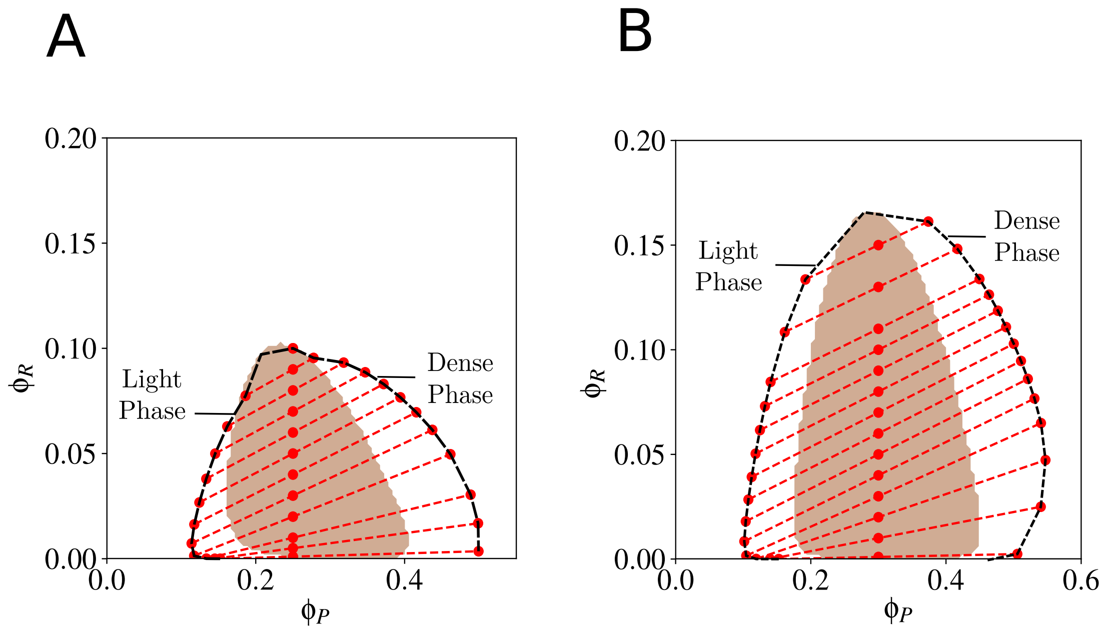

Figure S4: (A) Phase diagram for the Protein-RNA-Solvent ternary system described by $F_{FH}[\phi_P, \phi_R]$ at different protein (ϕ_P) and RNA concentrations (ϕ_R). The parameters used were $N_P = 5, N_R = 50, \chi_P = 1.1, \chi_{PR} = 1.2$ and $\chi_R = 2.0$. The brown region corresponds to the region of spinodal instability. The dotted red lines are coexistence curves that connect a point in the spinodal region to the protein-dense and the protein-light phase compositions (B) Phase diagram for the Protein-RNA-Solvent ternary system described by $F_{FH,mod}[\phi_P, \phi_R]$ using the same set of parameters.

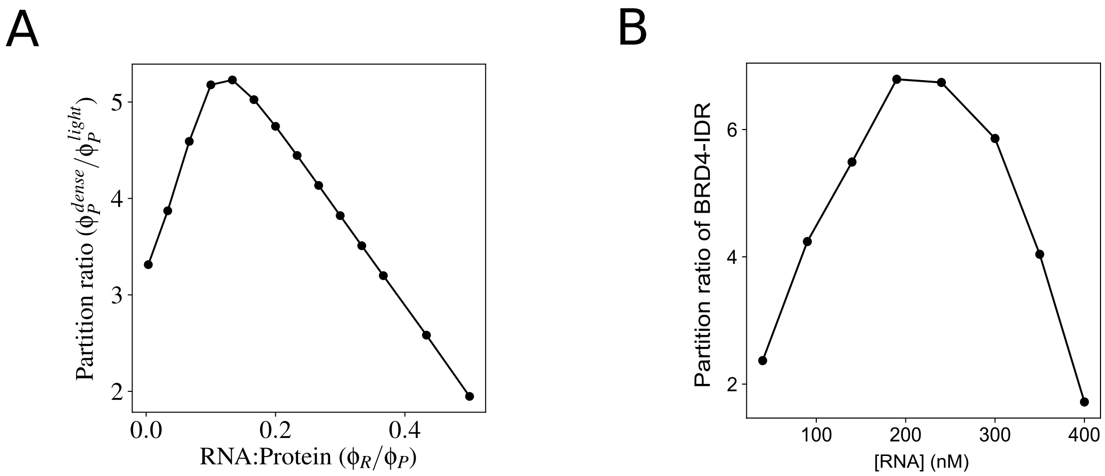

Figure S5: (A) Re-entrant phase diagram representing protein partition ratio $\phi_P^{dense}/\phi_P^{light}$ for a fixed protein concentration ($\phi_P = 0.3$) upon changing the RNA concentration ϕ_R calculated using the free energy $F_{FH,mod}$. The parameters used were $N_P = 5, N_R = 50, \chi_P = 1.1, \chi_{PR} = 1.2$ and $\chi_R = 2.0$. (B) The experimentally measured partition ratio of the transcriptional coactivator BRD4 in the condensate upon varying *Pou5f1* eRNA concentration in (10), figure S2D, exhibiting a re-entrant phase diagram. The parameters used in this study qualitatively match the experimentally measured phase diagram.

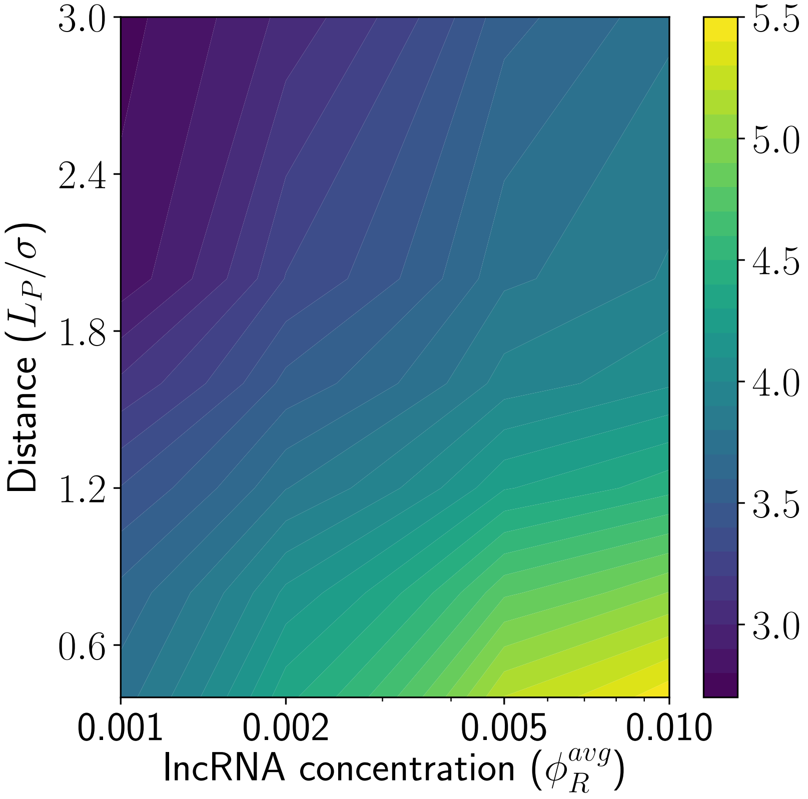

Figure S6: Phase diagram capturing how protein partitioning to the BL varies upon simultaneously varying the distance (L_P/σ) and the amount of lncRNA (ϕ_R^{avg}) in the system. The light white lines indicate contours, where the effects of the lncRNA amounts and the distance can compensate for each other to result in a similar protein partitioning to the BL

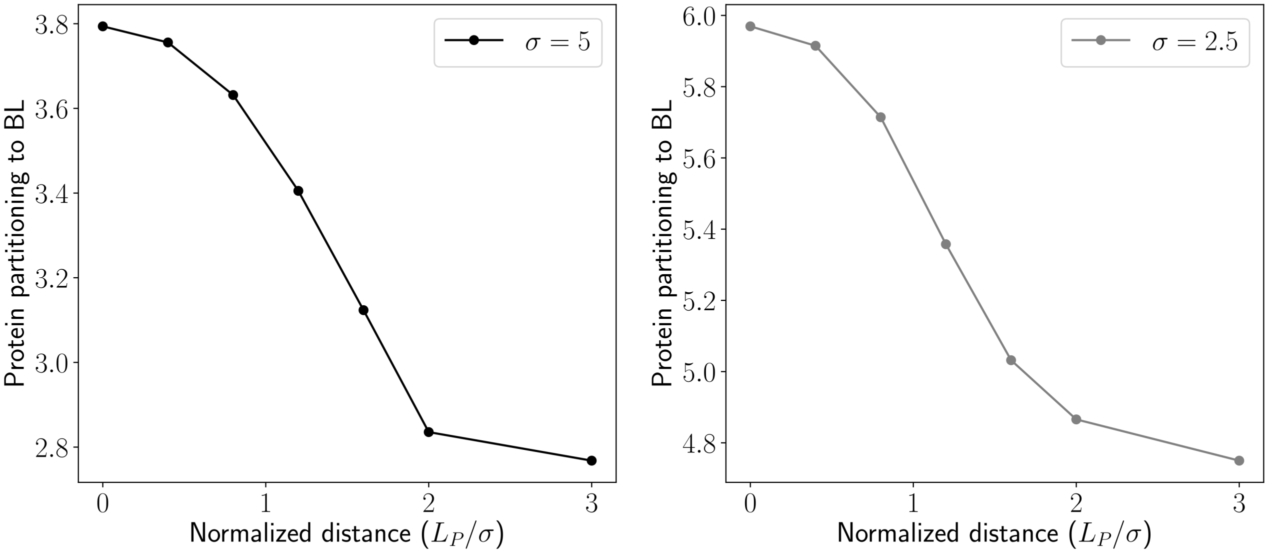

Figure S7: Protein partitioning to the BL upon varying the distance L_P between the BL and RL for two different values of the BL size σ. The trends show a qualitatively similar shape when the distances are graphed as the normalized distance L_P/σ.

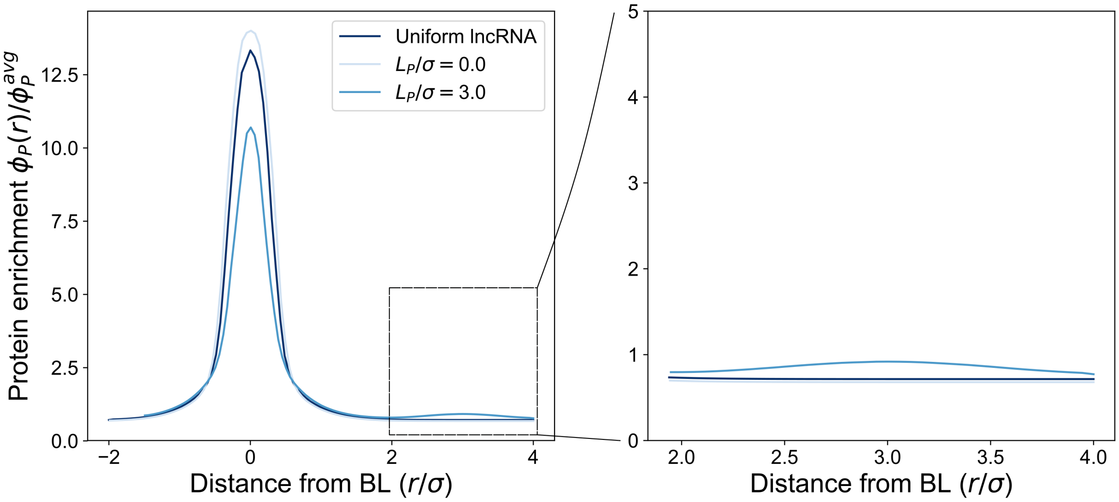

Figure S8: The protein concentration profile along the radial direction in the domain as measured by the protein enrichment ($\phi_P(r)/\phi_P^{avg}$) for three different cases (i) Uniform lncRNA: The are no lncRNA-RL interactions that keep it localized at the RL and it is present initially at a uniform concentration everywhere (ii) lncRNA is localized at the RL which also coincides with the BL (i.e. $L_P/\sigma = 0$) (iii) lncRNA is localized at the RL which is far away from the BL at a distance of $L_P/\sigma = 3$. When the lncRNA is localized at the BL i.e. $L_P/\sigma = 0$, it increases the protein enrichment at the BL compared to the case of uniform lncRNA. However, when the lncRNA at the RL is localized far away from the BL ($L_P/\sigma = 3$), the protein enrichment at the BL is reduced, due to a competition between the BL and the lncRNA accumulated at the RL to recruit the finite amount of available proteins. In this way, lncRNAs present at the RL far away from BLs can act as sponges to sequester away protein.

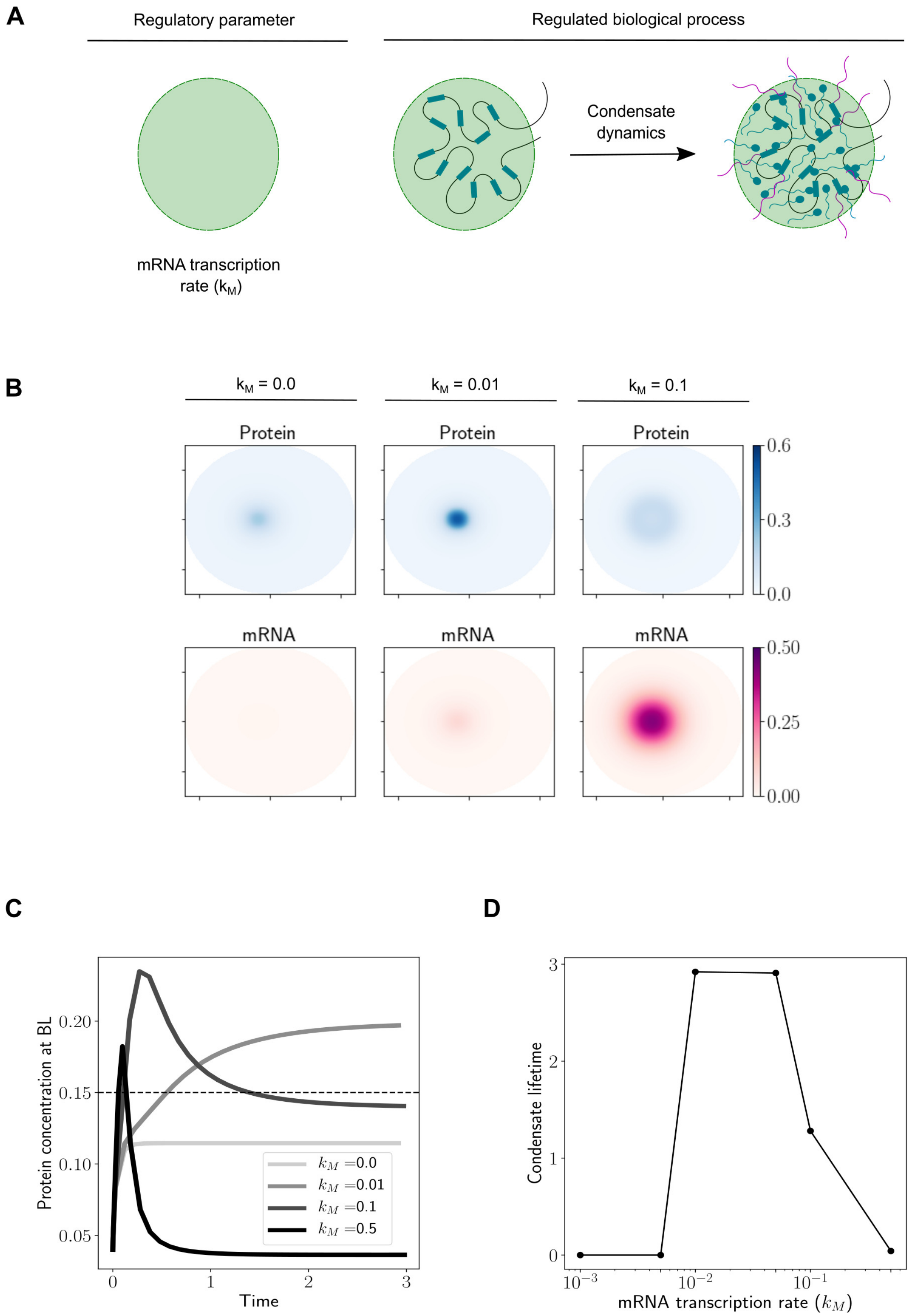

Figure S9: (A) In this figure, we will increase the mRNA transcription rate constant k_M and study how that impacts protein concentration at the BL and condensate lifetimes (B) Steady-state concentration profiles of protein (blue) and RNA (red) at steady state for different values of the transcription rate constant k_M (E) Dynamics of protein concentration at the BL (ϕ_P^{BL}) for different values of k_M. Time is in the dimensionless units of $k_{dM}t$ (D) The dependence of condensate lifetime on k_M. The condensate lifetime is also reported in the dimensionless units $k_{dM}t$, and is defined as the duration of time for which protein concentration at the BL is appreciable i.e. $\phi_P^{BL} > 0.15$.

SUPPORTING REFERENCES

1. Gil, N., and I. Ulitsky, 2020. Regulation of gene expression by cis-acting long non-coding RNAs. *Nature Reviews Genetics* 21:102–117. www.nature.com/nrg.

2. Cai, Z., C. Cao, L. Ji, R. Ye, D. Wang, C. Xia, S. Wang, Z. Du, N. Hu, X. Yu, et al., 2020. RIC-seq for global in situ profiling of RNA–RNA spatial interactions. *Nature* 582:432–437.

3. Beltran, B., D. Kannan, Q. Macpherson, and A. J. Spakowitz, 2019. Geometrical Heterogeneity Dominates Thermal Fluctuations in Facilitating Chromatin Contacts. *Physical Review Letters* 123:208103–208104.

4. Valouev, A., S. M. Johnson, S. D. Boyd, C. L. Smith, A. Z. Fire, and A. Sidow, 2011. Determinants of nucleosome organization in primary human cells. *Nature* 474:516–520.

5. Cho, W. K., J. H. Spille, M. Hecht, C. Lee, C. Li, V. Grube, and I. I. Cisse, 2018. Mediator and RNA polymerase II clusters associate in transcription-dependent condensates. *Science* 361:412–415.

6. Derrien, T., R. Johnson, G. Bussotti, A. Tanzer, S. Djebali, H. Tilgner, G. Guernec, D. Martin, A. Merkel, D. G. Knowles, et al., 2012. The GENCODE v7 catalog of human long noncoding RNAs: analysis of their gene structure, evolution, and expression. *Genome research* 22:1775–1789.

7. Shrinivas, K., B. R. Sabari, E. L. Coffey, I. A. Klein, A. Boija, A. V. Zamudio, J. Schuijers, N. M. Hannett, P. A. Sharp, R. A. Young, and A. K. Chakraborty, 2019. Enhancer Features that Drive Formation of Transcriptional Condensates. *Molecular Cell* 75:549–561.e7. https://doi.org/10.1016/j.molcel.2019.07.009.

8. Boija, A., I. A. Klein, B. R. Sabari, A. Dall'Agnese, E. L. Coffey, A. V. Zamudio, C. H. Li, K. Shrinivas, J. C. Manteiga, N. M. Hannett, B. J. Abraham, L. K. Afeyan, Y. E. Guo, J. K. Rimel, C. B. Fant, J. Schuijers, T. I. Lee, D. J. Taatjes, and R. A. Young, 2018. Transcription Factors Activate Genes through the Phase-Separation Capacity of Their Activation Domains. *Cell* 175:1842–1855.e16. https://doi.org/10.1016/j.cell.2018.10.042.

9. Hohenberg, P. C., and B. I. Halperin, 1977. Theory of dynamic critical phenomena. *Reviews of Modern Physics* 49:435–479.

10. Henninger, J. E., O. Oksuz, K. Shrinivas, I. Sagi, G. LeRoy, M. M. Zheng, J. O. Andrews, A. V. Zamudio, C. Lazaris, N. M. Hannett, et al., 2021. RNA-mediated feedback control of transcriptional condensates. *Cell* 184:207–225.

11. Lin, Y. H., J. P. Brady, J. D. Forman-Kay, and H. S. Chan, 2017. Charge pattern matching as a 'fuzzy' mode of molecular recognition for the functional phase separations of intrinsically disordered proteins. *New Journal of Physics* 19.

12. Quinodoz, S. A., J. W. Jachowicz, P. Bhat, N. Ollikainen, A. K. Banerjee, I. N. Goronzy, M. R. Blanco, P. Chovanec, A. Chow, Y. Markaki, et al., 2021. RNA promotes the formation of spatial compartments in the nucleus. *Cell* 184:5775–5790.

13. Werner, M. S., and A. J. Ruthenburg, 2015. Nuclear Fractionation Reveals Thousands of Chromatin-Tethered Noncoding RNAs Adjacent to Active Genes. *Cell Reports* 12:1089–1098.

14. Knauss, J. L., N. Miao, S.-N. Kim, Y. Nie, Y. Shi, T. Wu, H. B. Pinto, M. E. Donohoe, and T. Sun, 2018. Long noncoding RNA Sox2-ot and transcription factor YY1 co-regulate the differentiation of cortical neural progenitors by repressing Sox2. *Cell death & disease* 9:1–13.

15. Schede, H. H., P. Natarajan, A. K. Chakraborty, and K. Shrinivas, 2022. Organization and regulation of nuclear condensates by gene activity. *bioRxiv* .

16. Jung, C., P. Bandilla, M. von Reutern, M. Schnepf, S. Rieder, U. Unnerstall, and U. Gaul, 2018. True equilibrium measurement of transcription factor-DNA binding affinities using automated polarization microscopy. *Nature communications* 9:1–11.

17. Wu, L., P. Murat, D. Matak-Vinkovic, A. Murrell, and S. Balasubramanian, 2013. Binding interactions between long noncoding RNA HOTAIR and PRC2 proteins. *Biochemistry* 52:9519–9527.

18. Wei, M. T., Y. C. Chang, S. F. Shimobayashi, Y. Shin, A. R. Strom, and C. P. Brangwynne, 2020. Nucleated transcriptional condensates amplify gene expression. *Nature Cell Biology* 22. http://dx.doi.org/10.1038/s41556-020-00578-6.

19. Shi, K., T. Liu, H. Fu, W. Li, and X. Zheng, 2021. Genome-wide analysis of lncRNA stability in human. *PLoS computational biology* 17:e1008918.

20. Gu, B., T. Swigut, A. Spencley, M. R. Bauer, M. Chung, T. Meyer, and J. Wysocka, 2018. Transcription-coupled changes in nuclear mobility of mammalian cis-regulatory elements. *Science* 359:1050–1055.

21. Sabari, B. R., A. Dall'Agnese, A. Boija, I. A. Klein, E. L. Coffey, K. Shrinivas, B. J. Abraham, N. M. Hannett, A. V. Zamudio, J. C. Manteiga, C. H. Li, Y. E. Guo, D. S. Day, J. Schuijers, E. Vasile, S. Malik, D. Hnisz, T. I. Lee, I. I. Cisse, R. G. Roeder, P. A. Sharp, A. K. Chakraborty, and R. A. Young, 2018. Coactivator condensation at super-enhancers links phase separation and gene control. *Science* 361:eaar3958.

22. Muskovic, W., E. Slavich, B. Maslen, D. C. Kaczorowski, J. Cursons, E. Crampin, and M. Kavallaris, 2022. High temporal resolution RNA-seq time course data reveals widespread synchronous activation between mammalian lncRNAs and neighboring protein-coding genes. *Genome Research* 32:1463–1473.

23. Cho, W. K., N. Jayanth, B. P. English, T. Inoue, J. O. Andrews, W. Conway, J. B. Grimm, J. H. Spille, L. D. Lavis, T. Lionnet, and I. I. Cisse, 2016. RNA Polymerase II cluster dynamics predict mRNA output in living cells. *eLife* 5:1–31.
